# Assessing the implementation of physical activity-promoting public policies in the Republic of Ireland: a study using the Physical Activity Environment Policy Index (PA-EPI)

**DOI:** 10.1186/s12961-023-01013-6

**Published:** 2023-06-26

**Authors:** Kevin Volf, Liam Kelly, Aurelie Van Hoye, Enrique García Bengoechea, Ann MacPhail, Catherine B. Woods, The PEN Consortium

**Affiliations:** 1 Physical Activity for Health Research Cluster, Health Research Institute, University of Limerick, Limerick, Ireland; 2 Department of Physical Education and Sport Sciences, University of Limerick, Limerick, Ireland; 3 Research and Innovation Unit, Sport Ireland, Dublin, Ireland; 4 Office of the Vice President Research, University of Limerick, Limerick, Ireland; 5 Physical Education, Physical Activity and Youth Sport (PEPAYS) Ireland Research Centre, University of Limerick, Limerick, Ireland

**Keywords:** Physical activity, Public policy, Implementation science, Benchmarking, Stakeholder participation

## Abstract

**Background:**

Government policy can promote physical activity (PA) as part of a multilevel systems-based approach. The Physical Activity Environment Policy Index (PA-EPI) is a monitoring framework which assesses the implementation of government policy by drawing on the experience of national stakeholders. This study is the first to assess the extent of policy implementation in the Republic of Ireland using the PA-EPI tool, and to provide information on how policy implementation can be improved, with the intention of maximizing its impact on population levels of PA.

**Methods:**

This mixed-methods research study, comprising eight steps, was carried out in 2022. Information documenting the evidence for implementation of PA policy, across all 45 PA-EPI indicators, was collected via systematic document analysis, and validated via survey and interview with government officials. Thirty-two nongovernment stakeholders rated this evidence on a five-point Likert scale. Aggregated scores were reviewed by stakeholders who collectively identified and prioritized critical implementation gaps.

**Results:**

Of the 45 PA-EPI indicators, one received an implementation rating of ‘none/very little’, 25 received a rating of ‘low’ and 19 received a ‘medium’ rating. No indicator was rated as fully implemented. The indicators that received the highest level of implementation related to sustained mass media campaigns promoting PA and PA monitoring. Ten priority recommendations were developed.

**Conclusions:**

This study reveals substantial implementation gaps for PA policy in the Republic of Ireland. It provides recommendations for policy action to address these gaps. In time, studies utilizing the PA-EPI will enable cross-country comparison and benchmarking of PA policy implementation, incentivizing improved PA policy creation and implementation.

**Supplementary Information:**

The online version contains supplementary material available at 10.1186/s12961-023-01013-6.

## Introduction 

Physical activity (PA) contributes to reduced mortality [1], improved mental health outcomes [2, 3] and a lower burden of disease from noncommunicable diseases [4] (NCDs) and from infectious diseases [5]. PA participation has also been linked to social outcomes such as increased happiness [6] and social capital [7]. Hence, enabling people to engage in PA provides an opportunity for people to exercise a greater element of control over their own health and well-being.

In light of these health and social benefits, the WHO has published global targets seeking to promote PA [8]. The Global Action Plan on Physical Activity (GAPPA) aims for a 15% relative reduction in inactivity by 2025 [8]. However, studies of trends in PA levels reveal that inactivity levels have remained stubbornly unchanged thus far in the 21st century [9, 10]. This suggests that if these trends continue, the GAPPA target will not be met [11]. Therefore, new strategies for supporting healthy PA behaviours are required [9].

It has been argued that strategies targeting the so-called ‘upstream’ barriers to PA should be pursued [12]. In essence, this requires a strategy of building and implementing public policy [13, 14] in the domain of PA. Theoretical support for this idea comes from ecological models, widely used in public health research [15], which highlight the importance of policy action (for example, Sallis and colleagues [16]). Empirical support comes from review studies which identify social and environmental factors which influence population PA levels [17–20]. These findings suggest that policy action is necessary to affect changes to these environments to empower people to engage in healthier PA behaviours. Policy actions have been defined by Kelly and colleagues as ‘actual options selected by policy-makers. Public policy actions are specific actions put into place by any level of government or associated agencies to achieve the public health objective. They may be written into broad strategies, action plans, official guidelines/notifications, calls to action, legislation or rules and regulations. A policy action may have its own exclusive policy document or may be part of a larger document’. [21] [iv14] The WHO began to issue PA policy guidance documents in the mid-2000s [22], and the number of policies promoting PA has increased [23] with over 90% of countries globally having a national PA policy, though evidence exists that PA policy is not effectively operationalized [24, 25].

Alongside the rise in national PA policies, there has been a concomitant rise in the number of scientific publications concerning PA policy since the mid-2000s. This indicates the development of PA policy research as a scientific field [23, 26]. The maturing of PA policy research is also evidenced by the development of tools such as the WHO’s Health Enhancing Physical Activity Policy Audit Tool (HEPA PAT) [27], which facilitates comparative policy research, or the Comprehensive Analysis of Policy on Physical Activity (CAPPA) framework which categorizes PA policy research according to purpose of analysis, policy level under analysis, policy sector, type of policy, stage of the policy cycle and scope of the analysis [28].

Rütten and colleagues [26] highlight that there have been relatively few studies into how policy-making processes influence PA policy interventions. An example of how the policy process can influence PA intervention is through the extent of policy implementation (in essence, the processes by which policies are put into effect [29] [p. 12]). Research is needed to examine the extent to which policies that exist on paper are implemented in practice.

The Physical Activity Environment Policy Index (PA-EPI) is a monitoring framework recently developed to assess government policies and actions for creating a healthy PA environment (defined as the ‘context, opportunities and conditions that influence one’s PA choices and behaviours’ [30][p. 4]). The process of developing and validating the PA-EPI framework is described by Woods and colleagues [30]. The PA-EPI is conceptualized as a two-component ‘policy’ and ‘infrastructure support’ framework. The two components comprise eight policy and seven infrastructure support domains. The policy domains are education, transport, urban design, healthcare, public education (including mass media), sport for all, workplaces and community. The infrastructure support domains are leadership, governance, monitoring and intelligence, funding and resources, platforms for interaction, workforce development, and health-in-all policies. Forty-five ‘good practice statements’ (GPS) or indicators of ideal good practice within each domain concludes the PA-EPI. The eight-step process of conducting the PA-EPI will allow countries to identify areas of strength and weakness in the implementation of their national PA promoting policies, and potential actions needed to address critical implementation gaps. The PA-EPI results will provide data and examples of good practice in PA policy implementation, and it is envisaged that, in time, these examples will evolve to benchmarks as countries share knowledge and expertise on effective implementation processes.

The Republic of Ireland is the first country to have the extent of the implementation of its PA policies assessed using the PA-EPI. According to the Global Observatory for Physical Activity, less than half (46%) of the population of the Republic of Ireland engages in sufficient PA to meet health recommendations, and inactivity contributes to 8.4% of all deaths in Ireland [31]. Identifying implementation gaps in the PA policy response is part of the solution to increasing the proportion of the population meeting the PA guidelines, and to reducing the impact of inactivity. This study has two aims: the first is to identify critical implementation gaps by assessing the extent of PA policy implementation in the Republic of Ireland, and the second is to identify and prioritize actions that can strengthen policy implementation in Ireland.

## Methods

### Study design

**Fig. 1:**
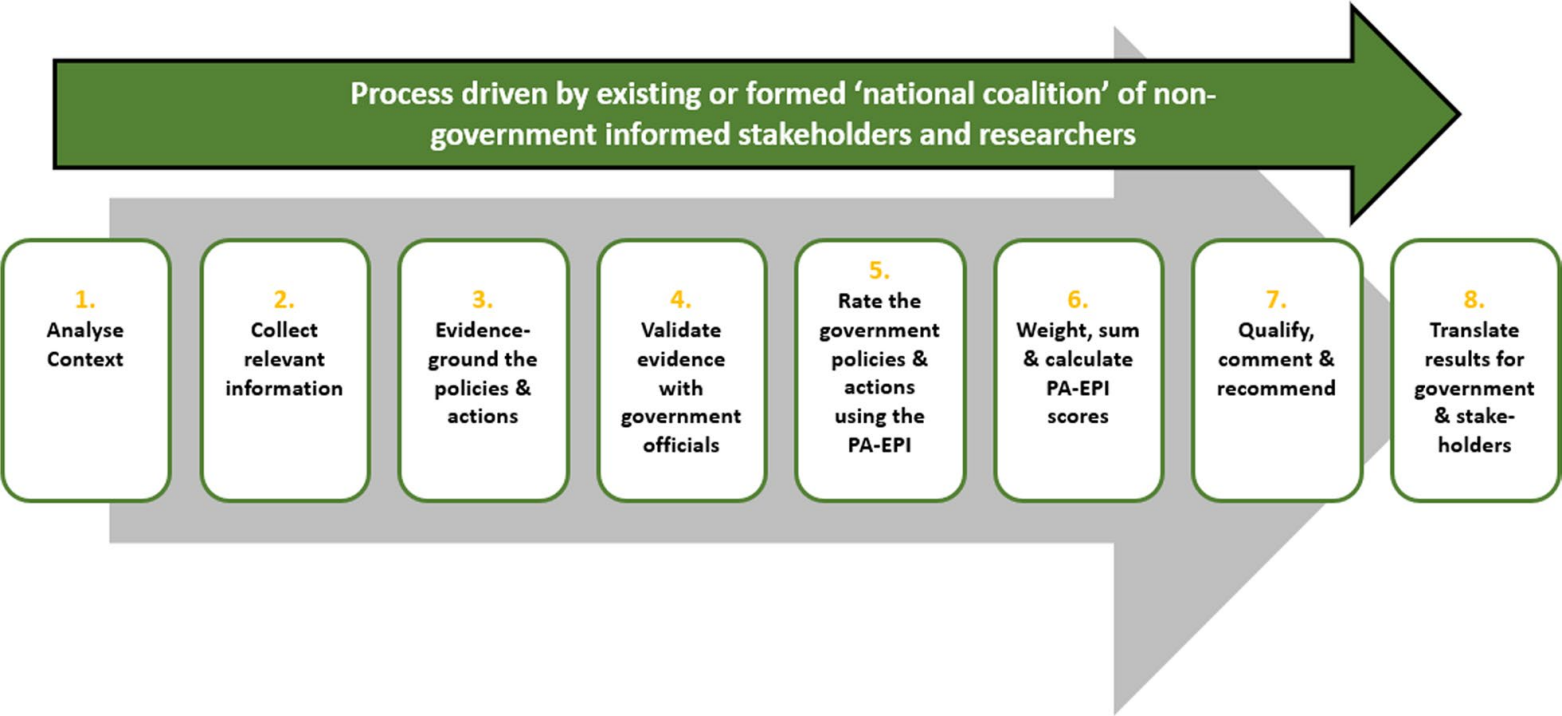
Process for applying the PA-EPI adapted from Swinburn and colleagues, 2013

This study is a sequential process, which combines both qualitative and quantitative methods. The INFORMAS network [32] developed the Food-EPI, on which the PA-EPI is based. They also designed a detailed eight-step process for completion of the Food-EPI, which has been accomplished in 40 countries worldwide [33]. Figure 1 shows this eight-step process, which was adapted for completion of the PA-EPI [30].

In brief, steps one to four involve the creation of an evidence document and its validation with government officials (see details below). Once complete and validated, quantitative data collection aimed at assessing the extent of implementation of the GPSs, using the evidence document, is undertaken by nongovernment PA stakeholders (step 5, see details below). From this ratings data, critical gaps in the implementation of PA policy are identified (step 6). The final two steps involve making recommendations for implementation actions (step 7) and dissemination of the PA-EPI results (step 8). Ethical approval for this study was necessary as steps 4, 5 and 7 required data collection from human subjects. Ethical approval was obtained from the Research Ethics Committee of the Faculty of Education and Health Sciences at the University of Limerick (2022_02_01_EHS).

**Fig. 2:**
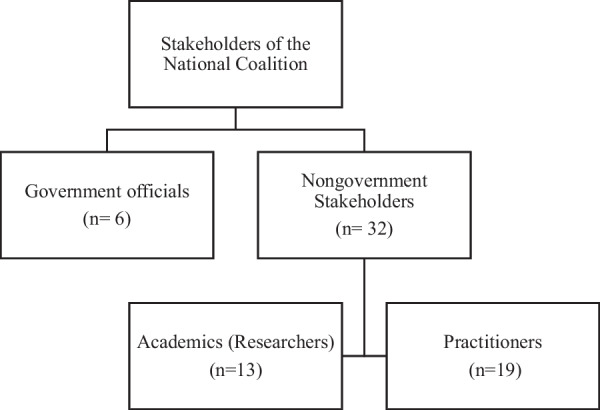
Categorization of stakeholders

The recruitment of a coalition of national stakeholders, two mutually exclusive groups: government officials and a panel of nongovernment PA stakeholders, is an important part of the PA-EPI process. The ‘government officials’ group include civil servants affiliated with governmental departments and high-ranking employees of state agencies. The inclusion of government officials is necessary to ensure that information on PA policy within the PA-EPI evidence document is comprehensive and accurate. The nongovernment PA stakeholders include researchers with knowledge of the PA environment and practitioners working for organizations promoting PA. The inclusion of nongovernment stakeholders supports engagement of civil society with the PA policy process (Fig. 2).

### Study procedure

To conduct the PA-EPI process in the Republic of Ireland, the eight steps briefly described above were followed.

#### Step one: analysing context

The first step of the process is to analyse the context of the country under study and decide which of the GPSs to utilize in the policy assessment and to begin drafting the evidence document. Some indicators of the PA-EPI may not be relevant for jurisdictions where there is substantial decision-making power devolved to subnational levels of government.

The Republic of Ireland is a unitary state with two levels of government, national and local level, established in accordance with Article 28A of the Constitution of Ireland. However, the local level of government has responsibility for a limited number of functions and its autonomy from national government is amongst the most limited in the EU [34]. Due to the level of involvement by national government for all indicators, the full list of PA-EPI indicators was retained without adaptation.

#### Step two: collecting relevant information

Step two involves collecting examples of implemented PA policies for each of the PA-EPI indicators from anywhere in the world. These were sourced from an analysis of WHO documents (for example, [35]), the academic literature and the PA policy experts consulted as part of the PA-EPI development process [30]. These examples are presented in the PA-EPI evidence document as Best Practice Exemplars (BPEs). They allow for comparison to the evidence of implementation to the country being studied, in this case the Republic of Ireland.

To collect national evidence of PA policy implementation in Ireland, several methods were used. Searches for evidence of implementation were undertaken in 2022. The first method was an audit of Ireland’s policy context for PA using the WHO Health Enhancing Physical Activity Policy Audit Tool (HEPA PAT) [36]. The HEPA PAT identified national policies pertinent to the promotion of PA, and provided information on the key policy documents (for example, ‘Get Ireland Active’: Ireland’s National PA Action Plan) and agencies (for example, Sport Ireland, Healthy Ireland) tasked with policy implementation. The second method, supplemented the HEPA PAT evidence, with internet searches of webpages of government departments and state agencies. A third method, which occurred simultaneously with internet searches, was extensive snowballing using the documents already identified. This involved reference checks of the included documents as well as searches using the titles of the documents to identify related documents such as action plans or implementation reports. Details of how the three methods were combined are displayed in Additional file 1.

The following was considered suitable evidence for inclusion in the evidence document: excerpts from formal written policy documents, (including statutes, guidelines and curricula), information from the websites of government departments or state agencies, information from websites identified with initiatives or programmes cited in written documents and academic literature describing PA policy implementation in the Republic of Ireland.

The following evidence was excluded from the evidence document: evidence of policies of local government and policies of nongovernmental bodies unrelated to public policy. Decisions on whether to include evidence in the evidence document were also informed by the wording and scope of the indicators, which is outlined in the evidence document.

#### Step three: evidence-grounding the actions

The third step was to extract information from the policy documents identified to populate the ‘Evidence of implementation in Ireland’ sections of the PA-EPI evidence document. Documents were scanned for lists or tables (for example, lists of actions) and keyword searches were performed within the documents based on the wording of each GPS. This evidence of implementation identified was summarized in short paragraphs and presented as tables for each of the 45 GPSs. As per protocol, draft one of the Irish PA-EPI Evidence Document was reviewed repeatedly by the research team before being prepared for validation by government officials.

#### Step four: validating evidence with government officials 

**Fig. 3:**
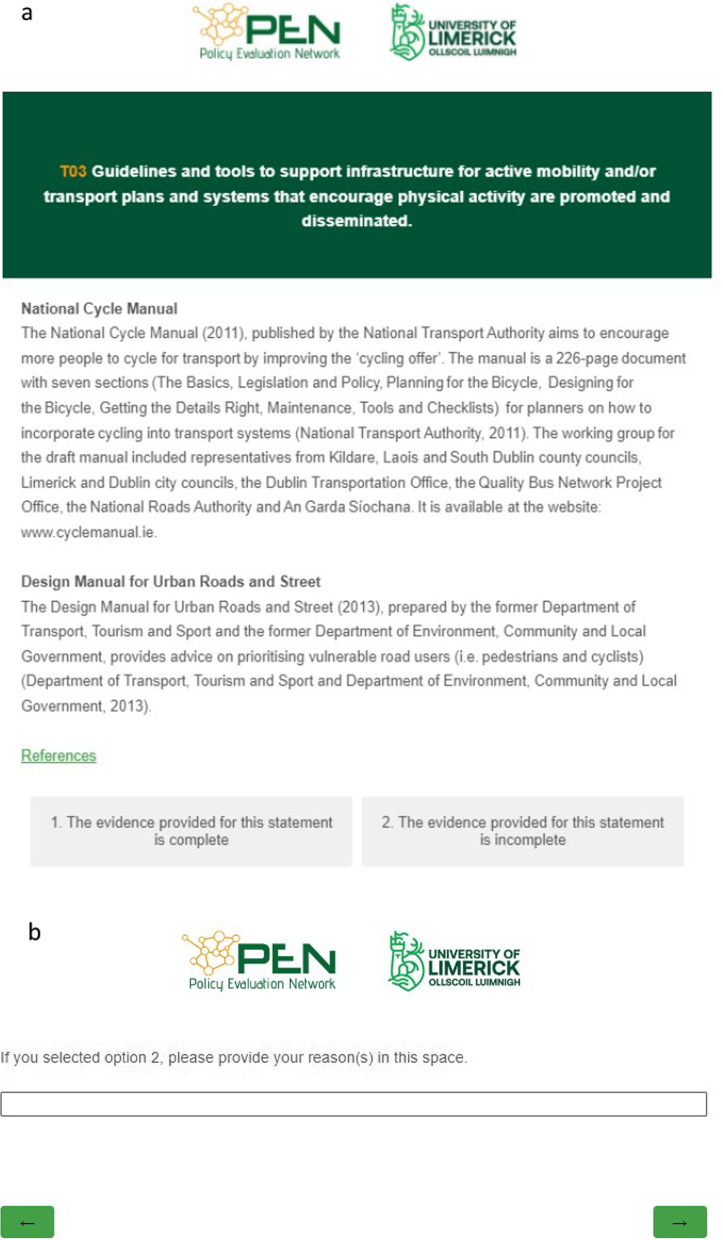
**a** and **b** Example from questionnaire sent to policy-makers

A purposive sample of government officials from different departments and agencies of the civil service was identified based on their roles, and/or prior collaborations with the PA research community in Ireland. The government officials were civil servants who had acted as representatives for their departments and agencies at PA events and whose role was identified from publicly available information. The research team reached out to the government officials via email and asked them to ensure the completeness of the evidence document. The email contained a link to an online questionnaire developed using Qualtrics software. Participants were provided with information about the study by a video embedded on the first page of the questionnaire, followed by a request for informed consent. If participants required further information, researchers answered their questions over a phone conversation. The questionnaire presented the government officials with the 45 GPSs of the PA-EPI, each on separate pages, above the evidence of implementation corresponding to the GPSs. Beneath the evidence of implementation was a questionnaire item which allowed the government officials to indicate amendments that needed to be made to make the evidence of implementation comprehensive. An example of the questionnaire layout is provided in Fig. 3a and b. Six government officials were contacted and four (two male, two female) provided feedback on the evidence document, resulting in 72 individual comments being made. The research team reviewed the comments and any relevant information identified as missing was carefully considered and added to a final draft of the evidence document.

#### Step five: rating the government policies and actions using the PA-EPI

**Fig. 4:**
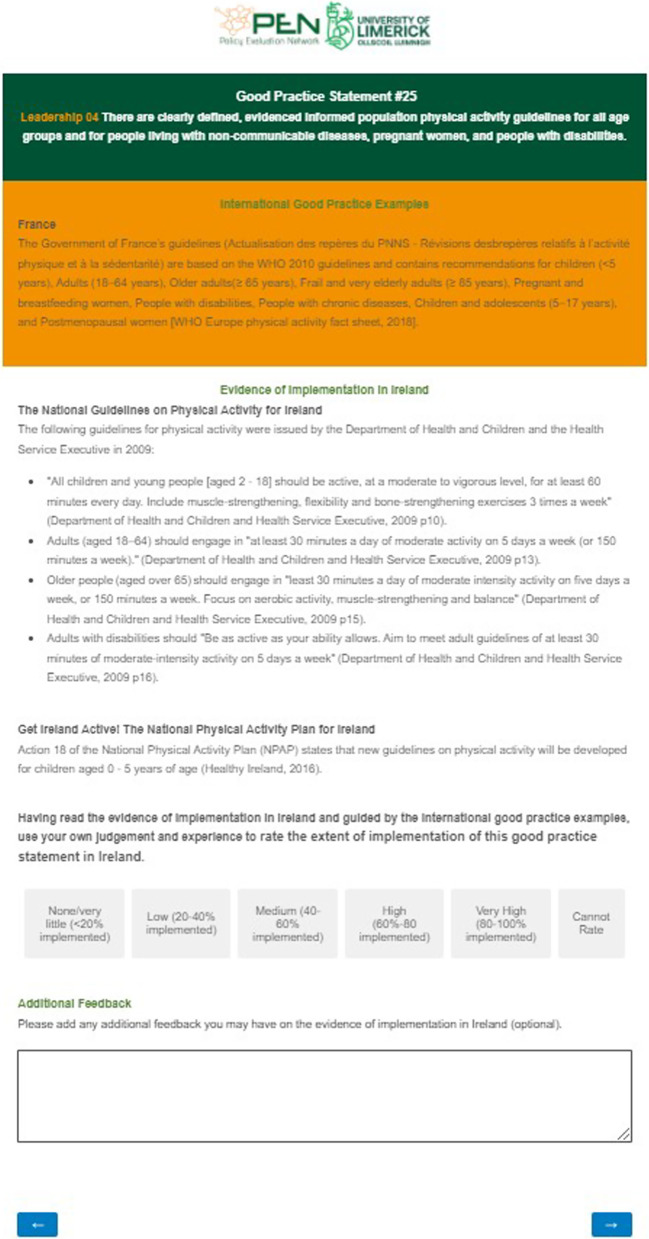
Example from the implementation rating questionnaire sent to nongovernment stakeholders

The fifth step was to assess the extent of implementation of the PA-EPI GPSs in the Republic of Ireland. A similar process to that used in the validation step was utilized for acquiring informed consent from participants in this step. Nongovernment PA stakeholders were identified either from their roles as researchers who have published on the topic PA in the Republic of Ireland or from their roles as PA promoters operating in Ireland. Nongovernment stakeholders were recruited via email and asked to complete an online questionnaire. Thirty-two individuals were contacted: 13 were academics (41%) and 19 of the nongovernment stakeholders were practitioners (59%). Practitioners included persons with a role promoting PA for local government or for nongovernmental organizations. Sixteen nongovernment stakeholders (50%) rated the extent of implementation of the GPSs of the PA-EPI in Ireland. Participants who accessed the questionnaire were asked to rate the evidence of implementation for each of the GPSs on a five-point scale. Participants were also provided with a ‘cannot rate’ option and the opportunity to comment on the implementation of each of the GPSs. An example of the format of the questionnaire is provided in Fig. 4.

#### Step six: weight, sum and calculate rating scores

The ratings scores were downloaded by the research team and the median rating was calculated for every indicator. Median was preferred over the mean as a measure of central tendency. The computed median scores where then utilized to categorize the extent of implementation as ‘very little/none’, ‘low’, ‘medium’ or ‘high’. Interrater Reliability (IRR; Gwet’s AC2 coefficient) was calculated for the implementation ratings using Agreestat software. The IRR for the implementation ratings was 0.554 (95% CI 0.495–0.612; percentage agreement 87%). The comments provided by the nongovernment stakeholders were also downloaded and implementation recommendations were extracted from these comments.

#### Step seven: qualify, comment and recommend

The seventh step involved a 1 day workshop to recommend policy implementation actions. All stakeholders were invited to attend in-person or online through Microsoft Teams. Six nongovernment stakeholders and two government officials participated in the workshop. Attendees were presented with the median rating scores for the implementation of the GPSs in the Republic of Ireland and the implementation recommendations extracted from the comments in the previous phase and asked to contribute further recommendations. Attendees debated the wording of implementation recommendations. Some implementation recommendations were removed and wording of other implementation recommendations was revised by the research team considering attendees’ recommendations. The revised list of implementation recommendations was circulated to all workshop attendees by the research team via email for confirmation. Following the finalization of wording, a questionnaire was sent around to all nongovernment stakeholders asking them to select five implementation recommendations from the policy domains and rank them based on the criteria of importance, achievability and equity. These criteria are an adaptation of the criteria described by Vandevijvere and Swinburn [37] (in a protocol developed to guide researchers on how to use the Food-EPI, mentioned previously). These criteria are displayed in Additional file 3. Participants were also asked to select five implementation recommendations from the infrastructure support domains and rank them based on importance and achievability. Fifteen nongovernment stakeholders (47%) voted on the implementation recommendations generated at the workshop. The scores for importance and achievability were inverted (so the top ranked recommendation from an individual rating received a score of 5 and the fifth ranked recommendation received a score of 1) and summed together. The five implementation recommendations with the highest summed score were selected as the ‘priority’ implementation recommendations. The process of summation was conducted for recommendations on both the ‘policy’ and ‘infrastructure support’ components of the PA-EPI, yielding a total of ten priority implementation recommendations.

#### Step eight: translate results for government and stakeholders

An in-person dissemination workshop was conducted, and all participants were invited to attend. The workshop was a joint event organized in collaboration with other research teams involved in health promotion research in Ireland, including research utilizing the Food-EPI tool. The workshop featured guest speakers with expertise in researching healthy diet and PA promotion and a panel discussion between prominent food and PA policy stakeholders. The research team presented research underpinning the development of the PA-EPI and the implementation and prioritization findings. A dissemination report presenting the findings was published and copies were provided to all workshop attendees. Electronic versions of the dissemination materials were uploaded to the internet on a website associated with the project (www.jpi-pen.eu).

## Results

The process generates three outputs: (i) the evidence document that contains information describing the implementation PA-promoting public policy in Ireland, (ii) an implementation scorecard presenting the rating of the implementation status of PA policy in the Republic of Ireland (according to expert opinion) and (iii) a list of implementation actions for improving the healthiness of the PA environment in the Republic of Ireland. The evidence document is available in the Additional file 2, the results of the implementation rating exercise is described in Sect. 3.1 ‘Level of implementation of physical activity environment policy in Ireland’ and the prioritization exercise is described in 3.2.

### Level of implementation of physical activity environment policy in Ireland

**Fig. 5:**
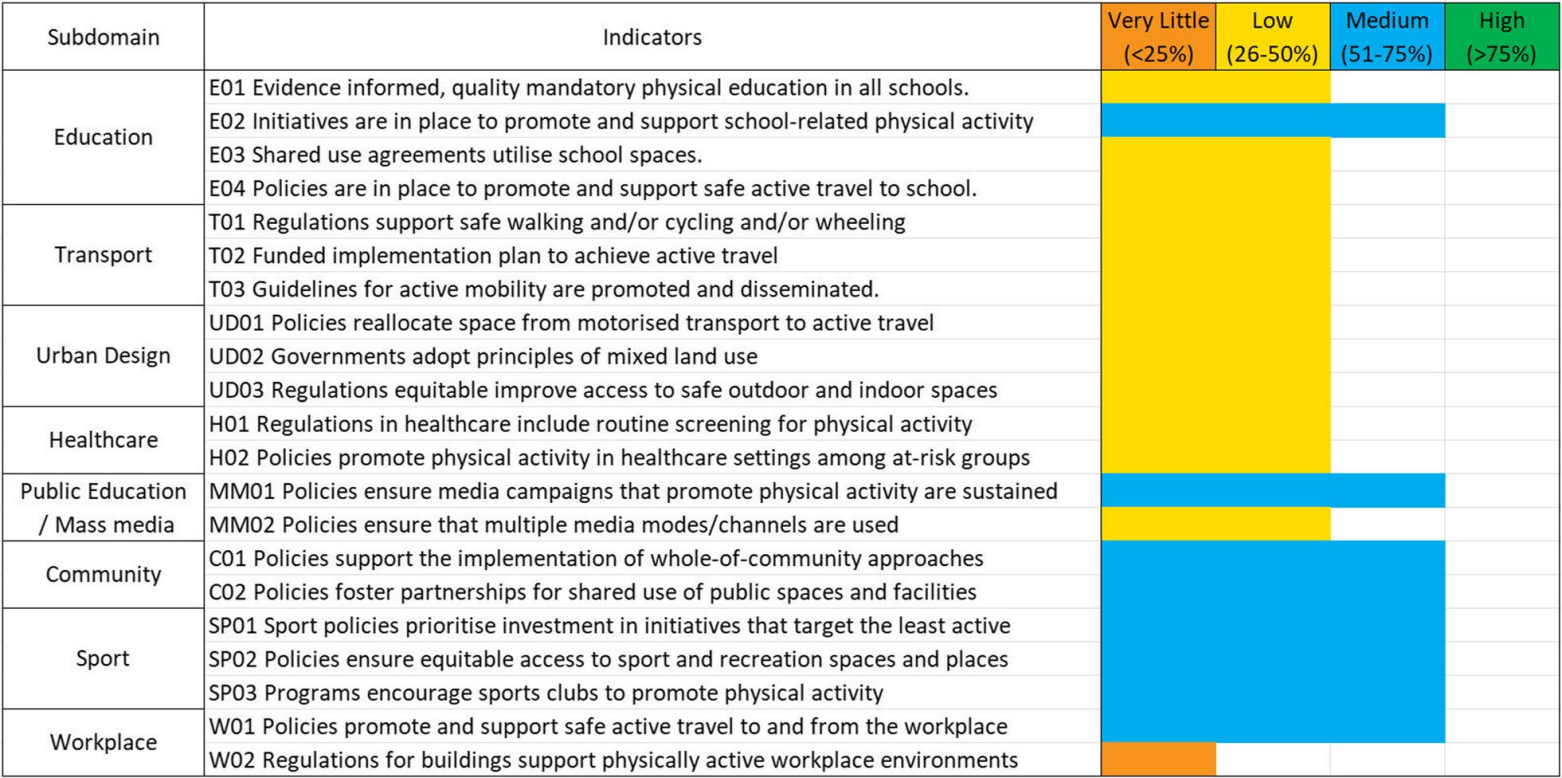
Results of the implementation rating for the policy-related domains of the PA-EPI in Ireland. The wording of each indicator is paraphrased to limit the amount of text within the graphic

The ‘policy’ subdomains of the PA-EPI framework contains 21 of the 45 GPSs. Twelve of the 21 GPSs (57%) received a low implementation score and 8 (38%) received a medium implementation score. One indicator (5%) received a ‘very little/none’ implementation rating from the expert panel. Three of the policy domains, Transport, Urban Design and Healthcare, were rated as having a ‘low’ level of implementation on every indicator. Two of the policy domains, Community and Sport were rated as having a ‘medium’ level of implementation on every indicator. These results are displayed in Fig. 5.

**Fig. 6:**
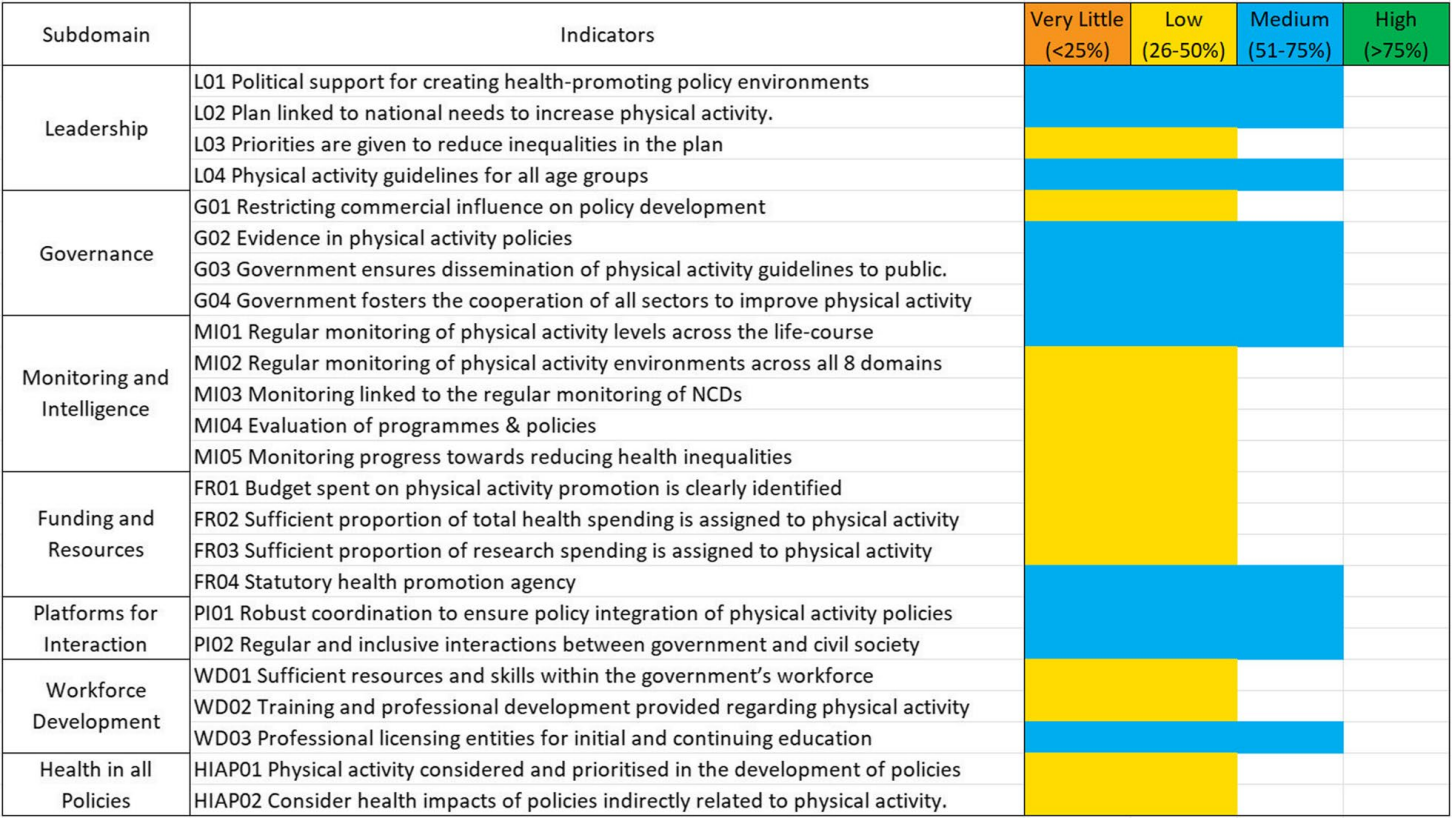
Results of the implementation rating for the infrastructure support-related domains of the PA-EPI in Ireland. The wording of each indicator is paraphrased to limit the amount of text within the graphic

The ‘infrastructure support’ subdomains contain 24 of the 45 GPSs. Thirteen of the GPSs received a low score and 11 received a medium implementation score. One of the infrastructure support domains, Health in all Policies, was rated as having a ‘low’ level of implementation on every indicator and one, Platforms for Interaction was rated as having a ‘medium’ level of implementation on every indicator. These results are displayed in Fig. 6.

None of the indicators received the highest categorization of implementation status. The highest scoring indicator in the policy domains was the first indicator in the ‘Mass Media’ subdomain, which pertains to public policies for sustaining mass media campaigns. The action of promoting PA through media campaigns is mentioned in several policy documents including the National Sports Policy [38] and NPAP [39]. Further, the Republic of Ireland has various media campaigns that promote PA, including the ‘Let’s Get Back’ campaign which encouraged the Irish public to be physically active during the COVID-19 emergency.

The highest scoring indicator in the infrastructure support domains was the first indicator in the ‘Monitoring and Intelligence’ subdomain, which pertains to the monitoring of PA levels across the life course. The Republic of Ireland has several surveys which collect data on PA levels, focusing on different stages of the life course. The Children’s Sport Participation and Physical Activity [40] (CSPPA) study, for example, examines sport and PA participation in children aged 10–19 years while the Irish Longitudinal Study on Ageing [41] (TILDA) includes data collection on PA in an older population. However, the other indicators in the monitoring and intelligence subdomain, (that is, the monitoring of PA environments, the monitoring of links between PA outcomes and NCDs, the monitoring of the outcomes of PA policy and the monitoring of inequality-related determinants of PA) all received a low rating.

The low implementation scores for the indicators related to Transport, Urban Design, Healthcare and Health in all Policies identifies a need for heightened efforts to address the implementation gaps in these domains.

### Prioritization of implementation actions

The top five implementation recommendations for policy and infrastructure support based on importance and achievability are presented in Tables 1 and 2. Regarding policy domains, the expert panel recommended that positions with responsibility for promoting PA be established in school, and health and social care settings. They also recommended increasing the capacity of health and social care staff to promote PA, replacing standalone PA campaigns with a long-term coordinated effort to promote PA opportunities in the media and the establishment of minimum criteria for inclusion before application for the sport capital grant are considered.

**Table 1:** Implementation actions to support healthy physical activity environments relating to the policy domains

*1. Leadership in schools [EDU8]*
Allocate a post of responsibility for a physical activity lead in every school, at both primary and post-primary levels
*2. Coordinated media campaign [MEDI1]*
Foster cross-governmental sustainable resourcing to replace standalone individual physical activity campaigns with a comprehensive, coordinated, multisector long-term multimedia/mode campaign using clear evidence informed consistent messaging over several years
*3. Minimum inclusivity standards [SPOR6]*
Establish a set of minimum inclusion and accessibility standards to be incorporated into the scoring system of the Sports Capital and Equipment Programme
*4. Connected community programmes [COMM2]*
Improve connection between communities and healthcare services in regard to physical activity participation by increasing the resourcing and/or staffing, with a go-to person for physical activity in the community
*5. Capacity of healthcare staff [HEAL2]*
Build capacity of staff across health and social care settings to promote awareness of physical activity benefits and opportunities

**Table 2:** Implementation actions to support healthy physical activity environments relating to the infrastructure support domains

*1. Update guidelines [LEAD1]*
Update the Irish Physical Activity Guidelines in line with revised international guidelines
*2. Representation in decision-making [GOVER3]*
Have representation across the lifespan, genders and socioeconomic backgrounds in the development and decision-making processes related to physical activity policies
*3. Funding for outcome monitoring [FUND1]*
Provide long-term funding for physical activity programmes to support tracking of evidence, outcomes and implementation
*4.Research programme for special populations [GOVER1]*
Implement a physical activity research and monitoring programme specific to special populations, in particular for disabled persons
*5. Dissociate from unhealthy products [GOVER2]*
Dissociate physical activity from unhealthy products and brands promoting unhealthy products

**Fig. 7:**
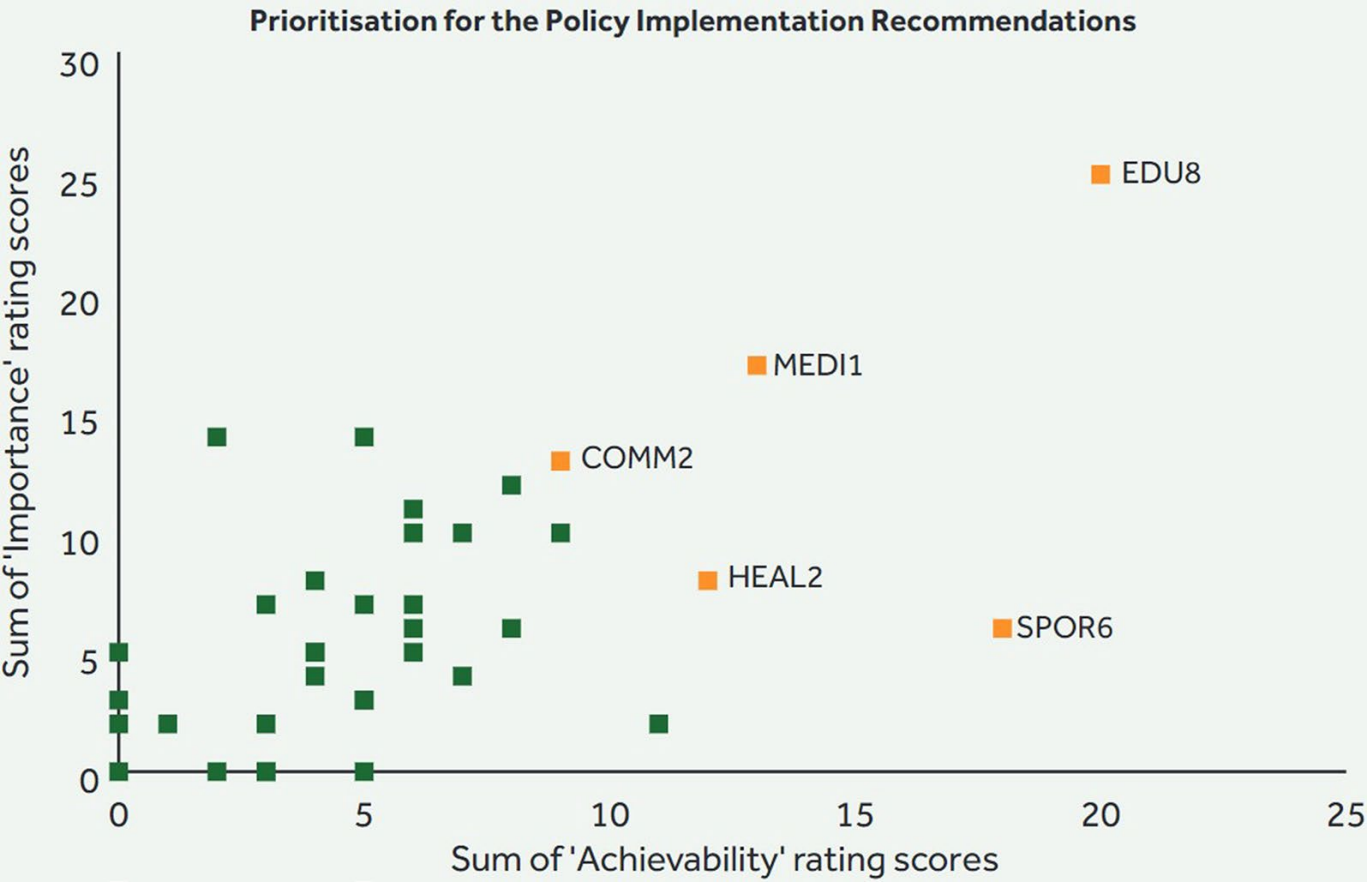
Prioritization of recommendations on the policy-related subdomains

**Fig. 8:**
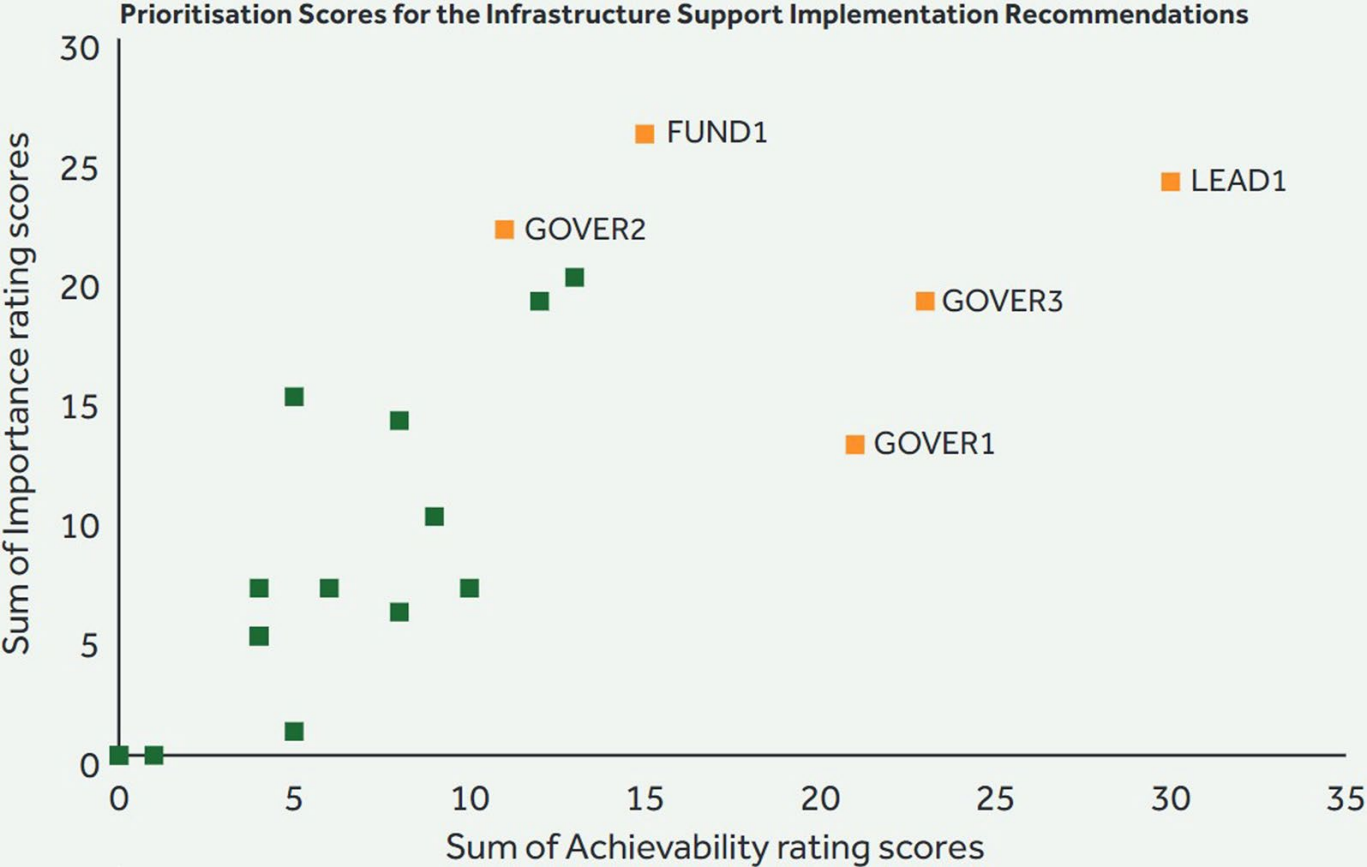
Prioritization of recommendations on the infrastructure support-related subdomains

Regarding the infrastructure support, the panel recommended increased funding for long-term PA projects for the monitoring programme outcomes. They also recommended ensuring representation across lifespan, genders and socioeconomic backgrounds in the decision-making process and to dissociate physical activity from unhealthy brands. The most highly rated recommendation, both in terms of importance and achievability, was to update the Irish PA guidelines to reflect recent advances in PA guideline development (Figs. 7, 8).

## Discussion

This study is the first to assess the extent of implementation of government policy actions which improve the PA environment. The process of assessing government actions generated an evidence document providing an overview of the government actions in place which supported PA, revealed areas of relative strength as well as gaps in implementation, and provided priority recommendations for strengthening PA policy implementation in the future. The evidence document was praised by stakeholders who participated in the study for providing them with an overview of the available policy documents in Ireland, which is an important contribution of the work in and of itself.

### Complementarity of the PA-EPI with other policy research resources

The process of generating the evidence document was supported by previous work using the HEPA PAT. The HEPA PAT has been recommended as a comprehensive tool for performing PA policy analysis [23] and it has been utilized in other European countries to conduct analyses of PA policy. However, reviewers of extant PA policy tools have noted that the PAT is ‘more suitable for an audit than an assessment’ [23] (p. 9) and further, that researchers should look into the possibility of complementary tools. This study highlights the complementarity of the PA-EPI tool with other instruments available to PA policy researchers, such as the HEPA PAT. It also demonstrates the additional benefit of using the PA-EPI for benchmarking and analysing the state of policy implementation. PA-EPI studies can provide unique information on implementation gaps that should be targeted to develop supportive PA environments.

### Implementation strengths and gaps

The results of this study reveal that the infrastructure support domains were judged to be better implemented than the policy domains. This is a nearly universal pattern for studies utilizing the Food-EPI [42, 43]. Further studies will reveal whether a similar pattern emerges for the PA-EPI as well and hopefully provide insight into the dynamics underlying these patterns. The implementation status of the indicators suggest that the Republic of Ireland can build on its relative strengths in the Mass Media and Monitoring and Intelligence domains. However, the results of the study also suggest that there are implementation gaps regarding Transport, Urban Design, Healthcare and Health in all Policies.

The low implementation ratings in the Healthcare domain appears to corroborate previous research on PA promotion by healthcare professionals in Ireland. Cantwell and colleagues [44] reported that most healthcare professionals in Ireland did not provide cancer patients with PA advice that aligned with guidelines, while Cunningham and O’Sullivan [45] report that only 30% of healthcare professionals in Northern Ireland and the Republic report receiving adequate training for prescribing PA to older adults. The Republic of Ireland has a policy for promoting PA, among other lifestyle risk factors in healthcare settings, Making Every Contact Count (MECC). The findings of this study, and others which we have cited above, suggest that the implementation of MECC has not been a success. This may be explained, at least in part, by the fact that an internal report commissioned by the HSE found that the health service lacked organizational readiness for this intervention prior to its enactment, It is unsurprising, therefore, that the expert panel recommended that increasing the capacity of staff across health and social care setting to promote awareness of physical activity and better connecting community PA programmes and healthcare be implemented as a priority.

### Prioritization

The panel of nongovernment experts prioritized actions in the policy and infrastructure support components of the PA-EPI. In the policy domains, the panel recommended implementation actions in the Education, Healthcare, Mass Media, Community and Sport domains. A difference between the PA-EPI and the Food-EPI is that policy domains of the PA-EPI arguably represent a greater number of independent health promoting settings than the Food-EPI. There is a potential equity concern as targeting different settings may have disproportionate benefits for different demographics. A potential method for promoting equity is to limit the number of actions prioritized per domain.

Some of the highest prioritized actions corresponded to indicators that had a relatively strong implementation rating. An implementation recommendation that received a high prioritization rating was the proposal to establish a long-term coordinated effort to promote PA opportunities in the media. It is also noteworthy that stakeholders did not prioritize implementation recommendations in the Urban Design or Transport domains, despite the identified implementation gaps in these domains. Future research may explore apparent discrepancies between identified gaps and prioritized implementation recommendations.

### Strengths and limitations

This study is the first to utilize the PA-EPI tool to generate insight into PA policy and hence addresses a knowledge gap regarding the assessment of government action on the issue of PA. The PA-EPI is a pioneering approach in the domain of PA policy and is based on internationally developed and validated methods used in the domain of food policy. A second strength of the study is the independence of the stakeholders involved in rating and prioritization. The research process engaged government officials to ensure that the evidence document is comprehensive and the rating of implementation was conducted by people who were not incentivized to provide positive findings as government officials tasked with performing a self-assessment. A third strength is that the PA-EPI process promotes capacity building. By engaging with government and nongovernmental stakeholders from across sectors, the PA-EPI process promotes network building around the issue of PA. Further, the evidence document is a valuable resource for policy-makers and nongovernmental PA stakeholders.

This study has some limitations. The workshop component was attended by a small sample of stakeholders (*n* = 7 stakeholders, representing the Education, Sport, Community and Health sectors). Attendance at the workshop may have been affected by scheduling conflicts and the legacy of the COVID-19 pandemic or rates at that time may have affected the willingness of stakeholders to participate in an in-person workshop. The small sample creates the possibility that a particular viewpoint is overrepresented in the output of this exercise. The challenge of potential selection bias has been previously reported by Yamaguchi and colleagues [46], who used the same eight step process in completing the Food-EPI in Japan. Researchers need to consider, in the early stages of the process, how to ensure that the stakeholders involved in the later stages represent a variety of perspectives with differing domains of expertise. A second limitation is that the nongovernment stakeholders involved in the prioritization exercise (*n* = 13) may have been presented with too many implementation recommendations. Further, the implementation recommendations were not evenly distributed across the domains, with many recommendations pertaining to the Education domain, which in turn led to focus on one part of the life course. The number of recommendations presented may have biased the results of the prioritization exercise to favour actions which target children and younger demographics. While the number of recommendations provided to nongovernment stakeholders was reduced as part of the workshop, this process should be made highly rigorous to avoid any concerns. Researchers should consider methods for limiting the number of recommendations presented for prioritization both in total and per domain. A third limitation is the availability of information on best practice exemplars used for comparison in the evidence document. Early studies utilizing the Food-EPI tool noted that policies put forward as BPEs were often not evaluated for real-world impact and hence not ideal ‘gold standards’ [47]. A benefit of conducting further assessments utilizing the PA-EPI is that it will provide concrete examples of good practice for review and replication by other countries to address implementation gaps.

### Recommendations for future studies

A study of the relative contributions of the GPSs and policy subdomains is needed to develop a weightings system for the PA-EPI. The weighting system would assign a relative importance for each of the GPSs for creating healthy PA environments and allow the calculation of a single PA-EPI score for implementation at step six of the progress. This score facilitates a cross-comparison of national PA-EPI implementation ratings and advances the use of the PA-EPI as a PA policy benchmarking tool. Though the ratings provided by the expert panel in this study suggest that there is substantial scope to improve implementation status of PA policy in Ireland, future studies can confirm whether the Republic of Ireland is a pioneer on this issue. The benchmarking feature of the PA-EPI tool addresses a noted gap in the PA policy research literature [48].

Scoping reviews have demonstrated that PA policy research is overwhelmingly conducted in a few high-income countries [26, 49], indicating that the field of PA policy research needs to diversify. Further, inactivity is increasing in developing countries as the dynamics that drive inactivity in developed countries emerge or are adopted [50]. Therefore, testing the PA-EPI process in low- and middle-income countries should be a priority for future research.

## Conclusion

This study is the first to undertake a process of PA policy assessment using the PA-EPI tool. The study had two aims: (i) to assess PA policy implementation in the Republic of Ireland and (ii) to prioritize implementation actions for the future. Regarding the former, the extent of implementation was assessed for each of the 45 indicators of the PA-EPI and the results of these assessments suggests that PA policy in the domains of Transport, Urban Design and Healthcare have a low level of implementation in Ireland. By contrast the domains of Mass Media and Monitoring and Intelligence were perceived by nongovernment PA stakeholders to be better implemented in Ireland. Regarding the latter, priority actions were suggested by prioritization workshop attendees and a short list of recommendations, targeting different domains of the PA-EPI, are highlighted in this article. This study contributes to understanding of why public policy may fail to achieve the environment necessary for sustained improvements in population PA. It also provides a roadmap for improved policy implementation in Ireland. The utilization of nongovernment stakeholders has the potential to increase civil society’s input to the PA policy agenda.

## Supplementary Information

**Additional file 1. **Search Strategies for Evidence Document.

**Additional file 2. **Evidence Document.

**Additional file 3. **Criteria for Prioritisation.

## Data Availability

The datasets used and/or analysed during the current study are available from the corresponding author on reasonable request.
