# Supplementary material for: Assessing the implementation of physical activity-promoting public policies in the Republic of Ireland: a study using the Physical Activity Environment Policy Index (PA-EPI)
Source: Health Res Policy Syst. 2023 Jun 26;21:63. doi: 10.1186/s12961-023-01013-6 (PMC10291784; doi:10.1186/s12961-023-01013-6)
Supplement: Supplementary file 1 — Additional file 1. Search Strategies for Evidence Document. [file 12961_2023_1013_MOESM1_ESM.docx]

Additional file 1: Search Strategies for Evidence Document

Policy Domains

| E01  Evidence informed, quality mandatory physical education that promotes and supports the ideals of equity, diversity and inclusion and adheres to defined standards is part of the curricula in all schools. |
| --- |
| The documents *Get Ireland Active! National Physical Activity Plan for Ireland* (NPAP), *Healthy Ireland: A Framework for Improved Health and Wellbeing 2013 – 2025* (HIF) and *Healthy Ireland Strategic Action Plan 2021–2025* (HI SAP) were identified as important crosscutting policy documents from section three of the HEPA PAT. The internet was searched for these documents, using their titles as search terms, and the documents were downloaded. Several documents labelled as annual implementation reports for NPAP were also identified from this search and downloaded. The downloaded policy documents were checked for every indicator. Sections of NPAP were identified as relevant evidence for this indicator and summarised in the evidence document.  The policy document *National Sport Policy 2018 – 2027* was identified from HEPA PAT. The internet was searched for this policy document, using its title as a search term, and the document was downloaded. Sections of NSP were identified as relevant evidence for this indicator and summarised in the evidence document.  Members of the research team had worked on the *Children’s Sport Participation and Physical Activity Study* (CSPPA) and were aware of the findings which were relevant to policy implementation. The internet was searched for the CSPPA website for use as a reference.  The document *Physical Education Curriculum Specification* was identified from section 3 of the HEPA PAT. The internet was searched for this policy document, using its title as a search term and the document was downloaded. The relevant sections of the policy document were summarised in the evidence document.  The research team accessed the websites of the Department of Education, the National Council for Curriculum Assessment and https://www.curriculumonline.ie/ and the documents specifying the primary school and junior cycle PE curricula were identified and downloaded.  The first draft of the evidence document was validated by government officials. The research team received feedback directing them to the document *Participation Plan 2021-2024 Increasing Participation in a Changing Ireland*, the non-examinable Physical Education curriculum at senior cycle level and the survey *Lifeskills Survey*. A search of the internet was undertaken using the titles of these documents and the survey. |
| E02  National and/or subnational initiatives are in place to promote and support school-related physical activity both at school and in other settings. These initiatives should employ an inter-sectoral approach and collaborative multi-agency partnerships (e.g., links with out-of-school sports clubs, active breaks/recess, walking clubs). |
| The policy documents NPAP, HIF and HI SAP were checked for every indicator as they were identified as crosscutting policy documents. Sections of NPAP were identified as relevant evidence for this indicator and summarised in the evidence document. The policy document “*Get Active! Physical Education, Physical Activity and Sport for Children and Young People. A Guiding Framework*” was identified from section three of the HEPA PAT as potentially relevant for this indicator and downloaded after an internet search for the title of that document. *Active School Flag* was identified from NPAP as a potentially relevant national initiative, based on the wording of the indicator. An internet search was conducted using “Active School Flag” as a search term and the Active School Flag website (<https://activeschoolflag.ie/>) was utilised as a reference.  The implementation reports for NPAP were also checked and *Youth Physical Activity Towards Health* (Y-PATH) was identified as another potentially relevant national initiative. Similar to Active School Flag an internet search was undertaken (using the search term “Y-PATH”) for the Y-PATH website (<https://irishheart.ie/courses/y-path/>).  The NSP was checked for information relevant to this indicator and information on the relevant passages were added to the evidence document.  The first draft of the evidence document was validated by government officials. The research team received feedback directing them to the documents *Participation Plan 2021-2024 Increasing Participation in a Changing Ireland* and *Sports Action Plan 2021-2023*. A search of the internet was undertaken using the titles of these documents.  The validation by government officials also provided comments suggesting that several school-based initiatives that support the wellbeing agenda should be included in the evidence document. The names of these initiatives were searched and relevant initiatives were added to the evidence document “Initiatives supporting the school ‘Wellbeing’ Agenda”. |
| E03  There are shared use agreements that utilise school spaces. Community access is supported by initiatives to promote and support opportunities for physical activity for all persons outside of normal school hours. |
| The NSP was checked for information relevant to this indicator.  Community Sports and Physical Activity Hubs were identified as potentially relevant by snowballing from action 11 of NSP. A follow up internet search was undertaken using the search term “community sports hubs”.  The first draft of the evidence document was validated by government officials. The research team received feedback directing them to the document *Participation Plan 2021-2024 Increasing Participation in a Changing Ireland.* |
| E04  National and/or sub-national policies are in place to promote and support safe active travel to and from school. |
| *Smarter Travel: A Sustainable Transport Future. A New Transport Policy for Ireland 2009 – 2020* (STP) was identified from the HEPA PAT as a policy document that potentially contains important evidence for this indicator. The internet was searched for this document, using its title as a search term, and the document was downloaded. A related document, *Review of actions of Smarter Travel Policy*, was identified from this search. Both documents were checked for information relevant to this indicator.  The Green Schools initiative was identified through snowballing. The initiative was identified through STP and a search of the internet was undertaken using the search term “green schools travel programme”.  The first draft of the evidence document was validated by government officials. The research team received feedback directing them to the documents *Programme for Government: Our Shared Future* and *Participation Plan 2021-2024 Increasing Participation in a Changing Ireland*. |
| T01  Regulations are in place that provide a variety of infrastructures to support safe walking and/or cycling and/or wheeling, including measures to calm speed, reduce vehicle traffic and enhance active mobility. |
| STP was identified from the HEPA PAT as a policy document that potentially contains important evidence for this indicator. STP and *Review of actions of Smarter Travel Policy* were checked for information relevant to this indicator.  Similarly, the *National Cycle Policy Framework* (NCPF) was identified as a policy document that potentially contains important evidence for this indicator from the HEPA PAT. The internet was searched for this document using the documents title, identified from HEPA PAT, as a search term.  The internet was searched using the search terms “speed limits Ireland” and “speed limits legislation Ireland”. |
| T02  There is a funded implementation plan, led by the appropriate level/s of government, to achieve improvements in active travel and increased use of public transport. |
| STP was identified from the HEPA PAT as a policy document that potentially contains important evidence for this indicator. STP and *Review of actions of Smarter Travel Policy* were checked for information relevant to this indicator.  The HEPA PAT was checked for policy documents titles and the “national mitigation plan” was identified. An internet search was conducted using the search term “national mitigation plan”. It was found that the National Mitigation Plan was ruled to be unlawful by the Irish Supreme Court and new policies were required. More searches were conducted using the search terms “climate action plan” and “climate action plan 2019”.  The first draft of the evidence document was validated by government officials. The research team received feedback directing them to the document *Programme for Government: Our Shared Future*. |
| T03  Guidelines and tools to support infrastructure for active mobility and/or transport plans and systems that encourage physical activity are promoted and disseminated. |
| *National Cycle Manual* was identified through snowballing. The resource was identified through STP (action 2) and a search of the internet was undertaken using the search term “national cycle manual”.  The HEPA.  *Design Manual for Urban Roads and Streets*(DMURS) was identified through snowballing. The resource was identified from the *Review of actions of Smarter Travel Policy* and a search of the internet was undertaken for this resource using the search term. |
| UD01  Policies or regulations that take a “health in all” approach are adopted to reallocate space from motorised transport to active travel and/or recreation purposes. |
| The policy documents NPAP, HIF and HI SAP were checked for every indicator as they were identified as crosscutting policy documents. Sections of NPAP were identified as relevant evidence for this indicator and summarised in the evidence document.  STP and *Project Ireland 2040 National Planning Framework* were identified from the HEPA PAT as policy documents that potentially contain important evidence for this indicator. An internet search was conducted for the document *Project Ireland 2040 National Planning Framework* using its title as a search term and the document was downloaded. The relevant sections of both documents were summarised in the evidence document. |
| UD02  Governments adopt land use policies, and planning processes, consistent with principles of mixed land use, compact urban design, and/or provision of green open spaces to support physical activity and reduce motorised transport. |
| *Project Ireland 2040 National Planning Framework* was identified from the HEPA PAT as a policy document that potentially contains important evidence for this indicator. The relevant sections of the policy document were summarised in the evidence document. |
| UD03  There are guidelines and/or regulations that improve universal and equitable access to safe outdoor and indoor spaces and facilities where people can be physically active. |
| The policy documents NPAP, HIF and HI SAP were checked for every indicator as they were identified as crosscutting policy documents. Sections of NPAP were identified as relevant evidence for this indicator and summarised in the evidence document. *Ready, Steady, Play! A National Play Policy* was identified as potentially relevant by snowballing from action 15 of NPAP. An internet search was conducted to identify a downloadable copy of this policy document from its title.  *Better Outcomes, Brighter Futures, the national policy framework for children & young people 2014 – 2020* and NCPF were identified as a policy documents that potentially contains important evidence for this indicator from the HEPA PAT. An internet search was conducted to identify downloadable copies of these policy document from their titles.  DMURS and *The Permeability Best Practice Guide* were identified by snowballing from the document *Review of actions of Smarter Travel Policy*. |
| H01  Guidelines and regulations in healthcare include routine screening for physical activity and, for all insufficiently active patients, brief advice, and referral to appropriately trained practitioners and/or physical activity opportunities. |
| The policy documents NPAP, HIF and HI SAP were checked for every indicator as they were identified as crosscutting policy documents. Sections of NPAP were identified as relevant evidence for this indicator and summarised in the evidence document.  Researchers had access to physical copies of the National Exercise Referral Framework (NERF) document through their work on previous projects. An internet search was undertaken using the search terms “national exercise referral framework” and an online version of the document was downloaded. The document was consulted for both health indicators.  The first draft of the evidence document was validated by government officials. The research team received feedback directing them to the documents *Self-Management Support Framework*, *Exercise Referral for the Health Services Briefing Document* and *Making Every Contact Count*. An internet search was conducted to identify downloadable copies of these policy document from their titles. |
| H02  There are consistent policies for promoting and supporting physical activity in primary and secondary healthcare settings among at-risk groups, such as people with type 2 diabetes and older adults (e.g., protocols for the assessment of the physical activity capacity; accessible, affordable, and tailored physical activity programmes; and training for caregivers for delivering physical activity programmes within residential aged care). |
| The policy documents NPAP, HIF and HI SAP were checked for every indicator as they were identified as crosscutting policy documents. Sections of NPAP were identified as relevant evidence for this indicator and summarised in the evidence document.  Snowballing from action 29 of NPAP identified *Let’s Get Active!... to Improve Health and Wellbeing* as a policy document that potentially contains important evidence for this indicator. An internet search was conducted to identify a downloadable copy of this policy document from its title.  Snowballing from action 30 of NPAP’s 2019 implementation report identified Age and Opportunity as a potentially relevant initiative for this indicator. An internet search was conducted using the search term “age and opportunity carepals”.  Researchers had access to physical copies of the National Exercise Referral Framework (NERF) document through their work on previous projects. An internet search was undertaken using the search terms “national exercise referral framework” and an online version of the document was downloaded. The document was consulted for both health indicators.  The first draft of the evidence document was validated by government officials. Feedback from validation by Government officials directed researchers to the Sláintecare healthcare reform. An internet search was undertaken using the search term “slaintecare” |
| MM01  There are national and/or subnational public policies in place that ensure media and education campaigns that promote and support physical activity are sustained and monitored (e.g., by making them part of, or aligning them with, a national action plan on physical activity and the physical activity guidelines). |
| The policy documents NPAP, HIF and HI SAP were checked for every indicator as they were identified as crosscutting policy documents. Sections of NPAP and HIF were identified as relevant evidence for this indicator and summarised in the evidence document.  The Start campaign was identified from snowballing from HI SAP.  Snowballing from action 1 of NPAPs 2018 implementation report identified the *Healthy Ireland Communication and Citizen Engagement* and *Feel Good Together* campaigns. Internet searches were undertaken using the search terms “healthy ireland communication and citizen engagement campaign” and “physical activity summer campaign”.  The *Let’s Get Set* campaign was identified by searching the internet using the search terms “healthy ireland active campaign” and the *Let’s Get Back* was identified by searching the internet using the search term “physical activity campaign Ireland”.  A search of the Sport Ireland website identified the *Women in Sport Policy* document and *the Keep Well* campaign as relevant. The *In This Together* campaign was identified as the predecessor to the *Keep Well* campaign. The website gov.ie was search with the search terms “in this together” and “#inthistogether”.  The internet was searched using the search term “covid-19 resilience and recovery plan”.  The first draft of the evidence document was validated by government officials. The research team received feedback directing them to action four of NSP and the documents *Participation Plan 2021-2024 Increasing Participation in a Changing Ireland* and *Sports Action Plan 2021-2023*. |
| MM02  There are clear, consistent policies to ensure that multiple media modes/channels (e.g., via posters, social media, radio as well as TV) combined with complementary community initiatives are used to promote the benefits of physical activity and disseminate guidelines which align with the WHO physical activity recommendations. |
| The policy documents NPAP, HIF and HI SAP were checked for every indicator as they were identified as crosscutting policy documents. Sections of NPAP and HIF were identified as relevant evidence for this indicator and summarised in the evidence document. |
| C01  Public policies are in place to support the implementation of whole-of-community approaches to promote physical activity and networking to strengthen resources and exchange experiences (e.g., WHO Healthy Cities, Active Cities, Partnerships for Healthy Cities). |
| The policy documents NPAP, HIF and HI SAP were checked for every indicator as they were identified as crosscutting policy documents and the NSP was checked for information relevant to this indicator.  The Healthy Cities and Counties of Ireland Network was identified by snowballing from HI SAP  Sport Ireland’s Active Cities initiative was identified through an internet search using the search terms “active cities Ireland”. The phrase ‘active cities’ was searched as it appears as an example in the wording of the indicator.  The first draft of the evidence document was validated by government officials. The research team received feedback directing them to the *Bike Week*, *Community Sports Hubs* and *European Week of Sport initiatives*. |
| C02  There are public policies in place to foster partnerships for shared use of public spaces and facilities for community-based and community-led physical activity programmes. |
| The policy documents NPAP, HIF and HI SAP were checked for every indicator as they were identified as crosscutting policy documents and the NSP was checked for information relevant to this indicator. Sections of NPAP were identified as relevant evidence for this indicator and summarised in the evidence document.  The policy document *Ready, Steady, Play! A National Play Policy* was identified from snowballing from action 15 NPAP. An internet search using the search term “national play policy” was undertaken to identify a downloadable copy of this policy document from its title.  The policy documents NSP, STP *Action Plan for Rural Development* and *Better Outcomes, Brighter Futures, the national policy framework for children & young people 2014 – 2020* were identified as a policy documents that potentially contains important evidence for this indicator from the HEPA PAT.  The policies *Outdoor Recreation Plan*(ORP) *and Strategy for the Future Development of National and Regional Greenways* through snowballing. Both documents are cited in action 17 of Review of actions of Smarter Travel Policy. An internet search was conducted to identify downloadable copies of these policy document from their titles.  The *Community Facilities Scheme* initiative was Identified from snowballing. The Action Plan for Rural Development implementation report 2018 action 64. An internet search using the search term “community facilities scheme” was undertaken to identify online resources related to the scheme. |
| SP01  There are national and/or subnational evidence informed 'Sport and Recreation for All' policies that prioritise investment in initiatives that target the least active, as well as disadvantaged groups. |
| The NSP was identified, from its title, as the primary policy document relevant for this indicator. The Sports Capital and Equipment Programme (SCEP) was identified from snowballing from the NSP. This was followed up by an internet search using the search term “sports capital programme”.  The Sport Ireland website was searched for relevant policy documents and *the Participation Plan 2021-2024 Increasing Participation in a Changing Ireland*, *Sport Inclusion Disability Charter, Sport Ireland Policy on Participation in Sport by People with Disabilities* and *Women in Sport Policy* were all identified as relevant for this indicator.  The HEPA PAT for Ireland document was checked for policy document titles and an internet search was undertaken using the search term “migrant integration strategy”.  The first draft of the evidence document was validated by government officials. The research team received feedback directing them to *Diversity and Inclusion Plan*. |
| SP02  There are national and/or subnational evidence informed policies or action plans in place that ensure equitable access to sport and recreation spaces and places for all. |
| The SCEP and the Large Scale Sport Infrastructure Fund (LSSIF) were identified from snowballing from the NSP. |
| SP03  There is government support for programs designed to encourage sports clubs to promote health-enhancing physical activity and other health behaviours (e.g., 'sports clubs for health' and 'health promoting sport clubs'). |
| The policy documents NPAP, HIF and HI SAP were checked for every indicator as they were identified as crosscutting policy documents. The GAA National Healthy Club Project was identified by snowballing from HI SAP.  The first draft of the evidence document was validated by government officials. The research team were directed to chapter 3 of the NSP.  The Community Sports and Physical Activity Hubs were identified by snowballing from the NSP. |
| W01  There are national and/or sub-national policy initiatives and infrastructure development programmes in place to promote and support safe active travel to and from the workplace. |
| STP was identified from the HEPA PAT as a policy document that potentially contains important evidence for this indicator. STP and *Review of actions of Smarter Travel Policy* were checked for information relevant to this indicator. Smarter Travel Workplace was identified by snowballing from action 8 of *Review of actions of Smarter Travel Policy* while the Cycle to Work Scheme was identified from action 11. |
| W02  There are concepts and regulations for buildings, plots and the environment in place that promote and support employers to create physically active workplace environments through building design and provision of adequate facilities (both indoor and outdoor). |
| The 2018 Finance Act was identified by Snowballing. The NSP suggests that changes to the tax code may be sought to incentive usage of gym equipment. The Finance Act amends the Taxes Consolidation Act 1997 |

Infrastructure Support Domains

| L01  There is strong, visible, political support (at the head of state/cabinet level) for creating health-promoting policy environments to improve population levels of physical activity and reduce inactivity related non-communicable diseases and their related inequalities. Political responsibility for health-related physical activity is clearly allocated within the governmental structures. |
| --- |
| The policy documents NPAP, HIF and HI SAP were checked for every indicator as they were identified as crosscutting policy documents.  The *Healthy Ireland Fund* was identified by snowballing from HIF and *the National Healthy Cities and Counties of Ireland Network* was identified from HI SAP.  The first draft of the evidence document was validated by government officials. The research team received feedback directing them to the documents the NSP, *Programme for Government: Our Shared Future, A Healthy Weight for Ireland, Obesity Policy and Action Plan 2016-2025, Healthy Workplaces Framework, Healthy Campus* and the initiatives *Sláintecare Healthy Communities Programme* and *Healthy Ireland Campaign* |
| L02  There is a comprehensive up-to-date plan (including timeline, targets, funding, priority policy and programme strategies) linked to national needs and priorities to increase population physical activity. |
| The relevant policy documents for this indicator were selected from section 3 of the HEPA PAT document. |
| L03  Priorities are given to reduce inequalities in relation to inactivity related non-communicable diseases in the comprehensive plan (above). |
| This indicator does not require a search as the content is based entirely on the previous indicator. |
| L04  There are clearly defined, evidenced informed population physical activity guidelines for all age groups and for people living with non-communicable diseases, pregnant women, and people with disabilities. |
| The policy documents NPAP, HIF and HI SAP were checked for every indicator as they were identified as crosscutting policy documents. Sections of NPAP were identified as relevant evidence for this indicator and summarised in the evidence document.  The national guidelines were identified from the HEPA PAT. An internet search was undertaken using the title of the guidelines document. |
| G01  There are reliable procedures to restrict commercial influences related to physical activity environments where there are conflicts of interest with improving population physical activity levels (e.g., restricting lobbying influences that limit physical activity opportunities). |
| An internet search using the google search engine was undertaken using the search terms “lobbying regulations ireland”.  Feedback from validation by Government officials was received directing the research team to the 2018 Public Health Act. The website Irish Statute Book (<https://www.irishstatutebook.ie/>) was searched for the relevant legislation |
| G02  There are procedures in place for using evidence in the development of physical activity policies. |
| The policy documents NPAP, HIF and HI SAP were checked for every indicator as they were identified as crosscutting policy documents. Sections of HIF and NPAP were identified as relevant evidence for this indicator and summarised in the evidence document.  The two physical activity-relevant actions of document *Healthy Weight for Ireland* were read and linked to the appropriate indicators.  The first draft of the evidence document was validated by government officials. The research team received feedback suggesting that evidence from NSP was relevant to this indicator.  Feedback from validation by Government officials was received directing the research team to the NSP, *Participation Plan 2021-2024 Increasing Participation in a Changing Ireland, National Sports Policy 2018 – 2027 Sports Action Plan 2021 – 2023* and the *National Sports Policy 2018 – 2027 Research Strategy*. |
| G03  The government ensures access to and regular dissemination of physical activity guidelines and key documents to the public. |
| The Physical Activity Guidelines were read, and a dissemination section was identified which was used to populate this section. |
| G04  The government fosters the cooperation and coordination of all sectors to align with strategic plans to improve the physical activity environment, and where appropriate, promotes civil society participation to develop and implement these plans. |
| The policy documents NPAP, HIF and HI SAP were checked for every indicator as they were identified as crosscutting policy documents. Sections of NPAP were identified as relevant evidence for this indicator and summarised in the evidence document.  Feedback from validation by Government officials was received and relevant elements from NSP, the Sports Action Plan and the participation plan were added. |
| MI01  There is regular monitoring of physical activity levels across the life-course based on representative samples, against guidelines/standards/targets. |
| Surveillance instruments identified from section 6 of the HEPA PAT survey, follow up searches, using names of surveys as keywords were on google internet search engine conducted to identify the associated online resources. Members of the research team had previous been involved in these surveys notably CSPPA. Follow up searches on google internet search engine conducted to identify relevant online resources. |
| MI02  There is regular monitoring of physical activity environments across all 8 policy domains (e.g., walkability, built environment). |
| The Childhood Obesity Surveillance Initiative (COSI) population survey identified from HEPA PAT. The Lifeskills survey and Sport Ireland’s National Trials Register were added following feedback from validation by Government Official. |
| MI03  Physical activity monitoring is systematically linked to the regular monitoring of non-communicable diseases and their related inequalities. |
| Healthy Ireland survey and Irish Sports Monitor identified from HEPA PAT document section 6. |
| MI04  There is regular research and evaluation of policies and major programmes to assess their effectiveness, process, and impact on achieving the goals of the physical activity and health plans. |
| The policy documents NPAP, HIF and HI SAP were checked for every indicator as they were identified as crosscutting policy documents. Sections of NPAP were identified as relevant evidence for this indicator and summarised in the evidence document. |
| MI05  Progress towards reducing health inequalities related to social and economic determinants of physical activity is regularly monitored. |
| The Healthy Ireland survey was identified through snowballing from HI SAP.  The paragraph on demographics modules of surveys was added after validation by government officials. |
| FR01  The budget spent on physical activity promotion across all policy domains is clearly identified and periodically monitored. |
| The HEPA PAT itself and the budgetary information contained therein, was added as a paragraph following feedback from the validation by government officials.  Details of local government expenditure were included following a suggestion from validation by government officials. Government official feedback provided details of the online location of spending reports.  Feedback from validation by Government officials was received and descriptions of the relevant elements of NSP were added. |
| FR02  There is a sufficient proportion of total health spending assigned to population physical activity promotion. |
| The details of total government budget was identified by searches of the website of the Department of Finance and the Department of Public Expenditure and the website <https://whereyourmoneygoes.gov.ie/en/>.  Government expenditure reports were identified from the website of the separate the Department of Public Expenditure.  Details of local government expenditure were included following a suggestion from validation by government officials. Government official feedback provided details of the online location of spending reports. |
| FR03  A sufficient proportion of total research spending is assigned to population physical activity promotion. |
| The policy documents NPAP, HIF and HI SAP were checked for every indicator as they were identified as crosscutting policy documents. Sections of NPAP and HIF were identified as relevant evidence for this indicator and summarised in the evidence document.  The Health Research Board is responsible for funding health research in Ireland. The HRBs website was searched for budget information.  The Sport Ireland Research Funding Scheme was added following validation by government officials which directed the research team to the relevant passages of the Sports Action Plan. |
| FR04  A secure funding stream is available for at least one statutory health promotion agency with an objective to improve population physical activity |
| The policy documents NPAP, HIF and HI SAP were checked for every indicator as they were identified as crosscutting policy documents. Sections of HIF were identified as relevant evidence for this indicator and summarised in the evidence document.  Details of the Health and Wellbeing division were identified from searches of the Health Service Executives website. The Health Service Executive is the state agency responsible for health services in Ireland. |
| PI01  There are robust coordination mechanisms across departments and levels of government to ensure policy coherence, alignment and integration of physical activity, and inactivity related non-communicable disease prevention policies across governments. |
| The policy documents NPAP, HIF and HI SAP were checked for every indicator as they were identified as crosscutting policy documents. Sections of NPAP and HIF were identified as relevant evidence for this indicator and summarised in the evidence document.  The two physical activity-relevant actions of document Healthy Weight for Ireland were read and linked to the appropriate indicators.  Feedback from validation by Government officials was received and relevant elements from the NSP and the Sports Action Plan were added. |
| PI02  There are structures and mechanisms for regular, meaningful, and inclusive interactions between government and civil society (academia, professional organizations, public-interest, non-governmental organisations, and citizens) on physical activity policies and other strategies to improve population physical activity and health. |
| The policy documents NPAP, HIF and HI SAP were checked for every indicator as they were identified as crosscutting policy documents. Sections of NPAP and HIF were identified as relevant evidence for this indicator and summarised in the evidence document.  Members of the research team were involved with the I-PARC. Details of I-PARC were added after using the I-PARC website (<https://i-parc.ie/>) as a reference.  Feedback from validation by Government officials was received and descriptions of the relevant elements of the NSP were added. |
| WD01  To address the challenge of population physical inactivity, there are sufficient resources and people with necessary skills within the government’s workforce (across all 8 policy domains). |
| The policy documents NPAP, HIF and HI SAP were checked for every indicator as they were identified as crosscutting policy documents. Sections of HIF were identified as relevant evidence for this indicator and summarised in the evidence document.  A professional working in the Health Service Executive was contacted and directed the research team to CORU which regulate health and social professions in Ireland including physiotherapists.  A search of the internet was undertaken using the search term ”teacher training in ireland”. The website of the Teaching Council of Ireland, the professional body for teaching in Ireland was accessed. From the website’s homepage, the pages on becoming a post-primary teacher (which includes Physical Education teacher) and Continuous Professional Development were accessed.  Feedback from validation by Government officials was received and details of the NSP, details of the activities of the inspectorate of the Department of Education and details of the activities of the Professional Development Service for Teachers where added. |
| WD02  Opportunities for training and professional development are provided to relevant individuals across multiple sectors (e.g., the 8 'Policy' domains) regarding the fundamentals of physical activity, its role in public health, and effective strategies for physical activity promotion. |
| The policy documents NPAP, HIF and HI SAP were checked for every indicator as they were identified as crosscutting policy documents. Sections of NPAP were identified as relevant evidence for this indicator and summarised in the evidence document.  A professional working in the Health Service Executive was contacted and directed the research team to CORU (website: https://www.coru.ie/) which regulates the health and social care professions in Ireland including physiotherapists. The research team was also directed to REPS Ireland - the register for exercise professionals in Ireland (website: <https://repsireland.ie/>).  Feedback from validation by Government officials was received and details of the relevant sections of the NSP where added. |
| WD03  Support and training systems are in place for relevant professionals (e.g., guidelines, toolkits, training workshops/modules/courses). To ensure uptake, accrediting agencies for professional education, and professional licensing entities should include minimum requirements for initial and continuing education in this domain |
| A professional working in the Health Service Executive was contacted and directed the research team to CORU (website: <https://www.coru.ie/>) which regulates the health and social care professions in Ireland including physiotherapists. The research team was also directed to REPS Ireland - the register for exercise professionals in Ireland (website: <https://repsireland.ie/>).  Feedback from validation by Government officials was received and details of the relevant sections of the NSP where added.  The website of Sport Ireland, the state agency responsible for the development of sport in Ireland, was searched using the search terms “coaching development”.  A search of the internet was undertaken using the search term ”teacher training in ireland”. The website of the Teaching Council of Ireland, the professional body for teaching in Ireland was accessed. From the website’s homepage, the pages on becoming a post-primary teacher (which includes Physical Education teacher) and Continuous Professional Development were accessed.  Feedback from validation by Government officials was received and details of the NSP, details of the activities of the inspectorate of the Department of Education and details of the activities of the Professional Development Service for Teachers where added. |
| HIAP01  There are processes in place to ensure that population physical activity and related health outcomes are explicitly and transparently considered and prioritised in the development of all government policies. |
| The policy documents NPAP, HIF and HI SAP were checked for every indicator as they were identified as crosscutting policy documents. Sections of HIF were identified as relevant evidence for this indicator and summarised in the evidence document.  Information on the Health Promotion Strategic Framework was identified by an internet search using the Google search engine using the search term “crosscutting health promotion policy ireland” |
| HIAP02  There are processes (e.g., health impact assessments) to assess and consider health impacts during the development of policies indirectly related to physical activity |
| The policy documents NPAP, HIF and HI SAP were checked for every indicator as they were identified as crosscutting policy documents. Sections of HIF were identified as relevant evidence for this indicator and summarised in the evidence document.  Information on the activities of the Institute of Public Health was identified by an internet search using the Google search engine using the search term “health impact assessment Ireland”. |
