# Supplementary material for: Assessing the implementation of physical activity-promoting public policies in the Republic of Ireland: a study using the Physical Activity Environment Policy Index (PA-EPI)
Source: Health Res Policy Syst. 2023 Jun 26;21:63. doi: 10.1186/s12961-023-01013-6 (PMC10291784; doi:10.1186/s12961-023-01013-6)
Supplement: Supplementary file 2 — Additional file 2. Evidence Document. [file 12961_2023_1013_MOESM2_ESM.docx]

Additional file 2: Evidence Document

Physical Activity Environment Policy Index: Policy Domains

DOMAIN 1 – EDUCATION

**There are public policies implemented that aim to impact on healthy physical activity environments and promote and support physical activity within the school setting.**

| **E01**  **Evidence informed, quality mandatory physical education that promotes and supports the ideals of equity, diversity and inclusion and adheres to defined standards is part of the curricula in all schools.** | |
| --- | --- |
| **Definitions and scope** | - Schools includes government and non-government primary and secondary schools (up to age 18 years). - Schools excludes early childhood education and care services (0-5 years). - Physical education Includes policies that relate to school PA programmes, where the programme is partly or fully funded, managed or overseen by the government. - Excludes programmes in schools that are targeted to children of low socioeconomic groups only. |
| **International best practice examples (benchmarks)** | **France**  A mandatory three hours of Physical Education (PE) per week is taught at primary school level with two mandatory hours at secondary school level. These requirements result in France devoting the most time to physical education of any country in Europe. The PE curriculum is structured around three aims (a) enrichment of motor skills (b) health education and management of physical life and (c) access to physical culture and sports. Physical Education is delivered by specialised teachers at secondary level (Weichselbaum et al., 2012).  **Belgium, Flanders**  Two hours of PE a week are compulsory for Flemish at primary and secondary level. The quality of Physical Education is scrutinized by the School Inspection Service (WHOROE and European Commission 2021).  **News South Wales, Australia**  In the Australian state of New South Wales between 1.5 and 2.5 hours of PE is required from kindergarten to year 6 (from ages 6 to 12 years approximately). 300 hours of Personal Development, Health and Physical Education (PDHPE) a year is required in years 7 – 10 (ages 12 to 16 years approximately; New South Wales Government, 2020). |
| **Context e.g. EU action / regulation** | **WHO**  **Global Action Plan on Physical Activity**  GAPPA contains 20 evidence-based policy actions for increasing rates of PA. Each action is associated with a list of sub actions, to be carried out by member states, the WHO secretariat, or other stakeholders. Action 3.1 of GAPPA states that member states should “strengthen provision of good-quality physical education”. The sub action 3.1.1 proposes that member states should ensure the provision of inclusive, quality, PE in primary and secondary schools (WHO, 2018).  **WHOROE**  **Physical activity strategy for the WHO European Region 2016–2025**  The European Physical Activity Strategy (EPAS) was developed by WHOROE as a strategy to promote PA amongst European citizens. The strategy suggests 14 objectives for European governments categorised into 5 priority areas. In the priority area ‘Supporting the development of  children and adolescents’, Objective 2.2 of EPAS (‘Promote physical activity in preschools and schools’) exhorts WHO member states in the European region to promote PA in schools. The activities that WHOROE recommends to fulfil this objective include providing an ‘adequate number’ of PE lessons. Furthermore, the lessons should be in accordance with the best scientific evidence and incorporate a wide variety of activities (WHOROE, 2016).  **EU**  **2013 Council recommendations**  In 2013, the European Council requested that the European Commission establish a HEPA monitoring framework based on a list of indicators provided by the council. Indicators 13 was developed to gather information about PE in schools at both primary and secondary level. Information sought by the indicator was the number of hours of PE at both levels, whether PE is mandatory or optional and whether it is regulated at a national or sub-national level (Council of the European Union, 2013).    **Eurydice**  Eurydice is a network supports cooperation in matters of education amongst European countries. It is part of the European Commission’s Erasmus+ initiative. Eurydice publishes reports that compares the educational systems of its 38 member states (European Commission, 2013). |
| **Evidence of implementation** | **Ireland**  **Get Ireland Active! National Physical Activity Plan for Ireland**  Action 11 of the National Physical Activity Plan (NPAP) states that the PE curriculum (described below) will be fully implemented, in accordance with department guidelines, in all primary and post-primary schools. Action 12 of the NPAP states that an annual evaluation of the quality of PE will be conducted in a sample of primary and post primary schools (Healthy Ireland, 2016 p36). Action 17 of NPAP states that the Lifeskills survey (see below) will be conducted every three years.    **National Sport Policy 2018 – 2027**  Action 2 of the National Sports Policy (NSP) states that the Department of Education and Skills and other stakeholders will ensure that PE supports the physical literacy of children (Department of Transport, Tourism and Sport, 2018 p102).  **Participation Plan 2021-2024 Increasing Participation in a Changing Ireland**  In 2021, the Participation Unit of Sport Ireland published the participation plan. The participation plan builds on NSP and its 54 actions are linked to actions in NSP. Action 34 states that Sport Ireland and its partners will “Support for the development of Secondary School PE Curriculum resource material in line with the new PE Curriculum” (Sport Ireland, 2021a; p19).    **Primary School Physical Education Curriculum**  In Ireland, the primary school physical education curriculum is outlined in the Physical Education Teacher Guidelines (Department of Education and Science, 1999). Physical Education (PE) is delivered by a generalist teacher along with the rest of the primary school curriculum (European Commission 2013; Coulter et al., 2020) The PE curriculum consists of six strands: Athletics, Dance, Gymnastics, Games, Outdoor and adventure activities and Aquatics (Department of Education and Science, 1999).    **Post Primary School Curricula**  Secondary school curriculum is divided into a junior cycle of 3 years and a senior cycle of 2 years with the option of an additional 'transition year' between junior and senior cycle.    **Junior Cycle - "Wellbeing"**  A new area of learning, titled “Wellbeing”, was added to the junior cycle curriculum in 2017. The subjects of Social, Personal and Health Education (SPHE), Civic, Social and Political Education (CSPE) and PE were incorporated into the new wellbeing programme. A minimum of 300 hours of timetabled engagement (aiming for 400 hours) across the three years of instruction are required for Wellbeing.  Guidelines for schools to provide a Wellbeing programme were issued by the National Council for Curriculum and Assessment (NCCA) the same year. The guidelines indicate that a minimum threshold of 135 hours should be allocated for PE, 70 for SPHE and 70 for CSPE across the three years of instruction. Other short courses, with resources developed by NCCA, may be offered as part of the Wellbeing programme (e.g. Philosophy, Digital Media Literacy). Schools have autonomy in delivering their Wellbeing programme but examples of programmes provided in the guidelines recommend a minimum of one double period a week (NCCA, 2017a). The resources developed by NCCA specify that the PE course is structured around four strands: Physical Activity for Health and Wellbeing; Games; Individual and Team Challenges; and Dance and Gymnastics (NCCA, 2017a). The materials specify that inclusive assessment practices are a key feature of teaching. Accommodations should be in line with the students need. An example that is provided is that a student who cannot speak must be provided with alternative means to communicate ideas. Instruction is conducted by a specialist PE teacher (European Commission, 2013).   **Senior Cycle – Physical Education Framework (Non-examinable Physical Education)**  The senior cycle physical education (SCPE) framework is a tool to assist teachers in planning Physical Education for all pupils at senior cycle. It recommends a minimum of one double period of PE per week. The framework introduces six ‘models’ or philosophies of Physical Education with different learning objectives which teachers can utilise to teach PE. The titles of these learning models are: Health-related physical activity, Sport education, Contemporary issues in physical activity, Adventure education, Personal and social responsibility and Teaching games for understanding (NCCA, 2017b).  **Senior Cycle**- **Physical Education Curriculum Specification (Examinable Physical Education)**  Senior cycle students have the option of studying PE as an examinable subject (Department of Education and Skills, 2017a). The curriculum for students who avail of this option consists of two strands. Strand 1 consists of learning about optimising performance. Strand 2 introduces contemporary issues in physical activity. The curriculum for PE as an examinable subject is designed to be delivered over 180 hours of instruction, with a recommendation of devoting 5 hours a week, incorporating a double period (Department of Education and Skills, 2017a).    **Children’s Sport Participation and Physical Activity Study (CSPPA) survey**  According to the Children’s Sport Participation and Physical Activity Study (CSPPA), conducted in 2018, 51% of primary school pupils reported receiving PE classes at least twice a week. However, 18% reported receiving less than 30 minutes of PE.  At the post primary level, 23% of pupils reported receiving 120 minutes of per or more. The average amount of PE received by pupils at post primary level was 89 minutes (Woods et al., 2018 p11).  **Lifeskills Survey**  The department with responsibility for overseeing education conducts a survey called the Lifeskills Survey intended to inform policy direction every three years (Department of Education and Skills, 2017b, see indicator Monitoring and Intelligence 02). In 2015, the Lifeskills Survey found that 94% of surveyed primary schools reported meeting the recommended time allocation of physical education for primary schools of one or more hours per week. The same survey stratified results for post primary schools by year group. It was found that 4% of schools reported meeting the recommended time allocation of physical education for post primary schools of two or more hours per week in the first-year cohort. Three per cent reported meeting the recommendation in the second-year cohort, 1% in the third-year cohort, 35% in the transitions year cohort and 0% in the final two years. |

| **E02**  **National and/or subnational initiatives are in place to promote and support school-related physical activity both at school and in other settings. These initiatives should employ an inter-sectoral approach and collaborative multi-agency partnerships (e.g., links with out-of-school sports clubs, active breaks/recess, walking clubs).** | |
| --- | --- |
| **Definitions and scope** | - Schemes, in this context, refers to a particular type of policy that supports programmes (through for example funding, materials, additional staff, interventions) - School-related physical activity includes activities outside of the school grounds - Includes standards for public sector school settings that promote school-related PA both inside and outside of classrooms. - Includes partnerships with ‘private companies’. - Includes partnerships with voluntary and for-profit sport organizations and extends to non-government organisations (NGOs). |
| **International best practice examples (benchmarks)** | **Finland**  **Schools on the Move Programme**  The Finnish Schools on the Move Programme is a government programmed to achieve a more active school workday. School staff report that the increased physical activity during the school day has contributed to great enjoyment of school. In 2016, 75% of comprehensive schools in Finland participated in the Schools on the Move programme (Blom *et al.,* 2016)  **Austria**  **Moving Children Healthy 2.0**  The Moving Children Healthy 2.0 is a nationwide programme designed to promote physical activity in primary schools and kindergartens (Fit Austria GmbH, 2016).  The programme achieves this by facilitating needs-based cooperation between schools and local sports clubs. Moving Children Healthily 2.0 is funded by the Ministry of Sport (Bundesministerium für Kunst, Kultur, öffentlichen Dienst und Sport) and the federal sports fund (Bundes-Sport GmbH) (World Cancer Research Fund, 2021).  **Australia, New South Wales**  **Active Kids**  The government of New South Wales runs a voucher programme entitled Active Kids. Every school-enrolled child in the state is eligible for two vouchers worth $100 Australian dollars per year. The voucher is used by parents to ameliorate the costs of participating in leisure time physical activity. The effectiveness of the programme has been evaluated and it has been found to significantly increase children’s PA. |
| **Context e.g. EU action / regulation** | **WHO**  **Global Action Plan on Physical Activity**  As well as advocating for good quality PE, action 3.1 of GAPPA advocates the adoption of a whole-of-school approach to PA promotion. Sub action 3.1.2 recommends to member states to implement whole of school programmes guided by the WHO’s “Health Promoting Schools” resource (WHO, 2007; WHO, 2018).  **WHOROE**  **Physical activity strategy for the WHO European Region 2016–2025**  Objective 2.2 of EPAS recommends to member states to employ an intersectoral approach to promoting PA in schools and preschools. Further, it recommends various measures to ensure implementation of PA promotion programmes in schools, including legislation (WHOROE, 2016).  **EU**  **2013 Council recommendations**  Indicator 14 of the EU HEPA monitoring network seeks information on whether member states have implemented a national or subnational scheme for school-related PA promotion (Council of the European Union, 2013). |
| **Evidence of implementation** | **Get Active! Physical Education, Physical Activity and Sport for Children and Young People. A Guiding Framework**  Get Active! Is a document published in 2012 by the Department of Education and Skills to promote physical activity and play in the following contexts:  1. In Class  2. In extracurricular activities  3. In the community.    There are 3 parts to the Get Active! Document. Part 1 (Get Active 1) outlines the desired outcomes of children’s and young people’s participation in physical education and physical activity. Part 2 (Get Active 2) guides the document's intended users through the process of implementation via a six-step process specifying the roles of key stakeholders (including boards of management, principals, parents and teachers). Part 3 (Get Active 3) outlines a framework for evaluation.  The Get Active! Guide is intended to support other initiatives such as Active School Flag (described below) (Department of Education and Skills, 2012). Action 8 of the National Physical Activity Plan (NPAP) commits to supporting schools implement the Get Active Framework (Healthy Ireland, 2016, p35).    **Youth Physical Activity Towards Health**  The Youth Physical Activity Towards Health (Y-PATH) programme aims to stop or ameliorate the declines in physical activity that occur in the teenage years by supporting schools. Y-PATH is a post-primary whole-of-school programme delivered by PE teachers. Participating schools can access resources which allow the teachers to better develop the knowledge, motivation and Fundamental Movement Skills (FMSs) of children. Y-PATH is a programme of the Irish Heart Foundation, an independent charity, and is recognised by the Professional Development Service for Teachers (PDST) and Sport Ireland (Irish Heart Foundation, n.d.).    **Active School Flag**  The Active School Flag (ASF) is an initiative developed by the Department of Education and Skills and supported by the Department of Health (Belton et al., 2020; Active School Flag, 2021). Established in 2009, ASF aims to create more physically active school environments utilising a multicomponent whole-school approach. The possibility of achieving ‘active school’ status incentivises schools to engage in a process of ensuring the school environment is conducive to daily physical activity.  Important features of the programme include seeking out consultation with children and young people as part of the decision-making and implementation processes and encouragement of schools to collaborate with parents and local organisations. Once awarded to a school, 'active school' status remains valid for three years.  By 2020, ASF had been adopted by more than 2000 primary schools (Belton et al., 2020) and presently 673 schools have 'active school' status (Active School Flag, 2021). For context, the Department of Education and Skills counted 3241 primary schools in the Republic in 2020 (Department of Education, 2021). A new ASF process for post primary schools is under development (Active School Flag, 2021).    **Get Ireland Active! National Physical Activity Plan for Ireland**  Action 9 of the National Physical Activity Plan (NPAP) commits to extend the ASF programme by a further 500 schools compared to baseline (Healthy Ireland, 2016, p35).  **National Sports Policy 2018 – 2027**  Action 7 of the National Sports Policy (NSP) states that messages about the positive relationship between sport participation and exam performance will be promoted among teachers and parents and guardians.  **Participation Plan 2021-2024 Increasing Participation in a Changing Ireland**  The actions of the participation plan are categorised into ‘themes’. There are 6 actions in the theme ‘Youth’ and four in the theme ‘Schools’. Action 25 is to develop a ‘Physical Literacy Consensus Statement’ and to promote it to schools. Action 26, 27 and 29 are concerned with investment in programmes which meet the objectives of the Physical Literacy Consensus Statement, focus on secondary school children and support teenagers who are dropping out of team sports to transition to individual sports respectively. Action 30 supports the delivery of after school physical activity programmes (Sport Ireland, 2021a).  **National Sports Policy 2018 – 2027 Sports Action Plan 2021 - 2023**  Action 2.2 of the Sport Action Plan also states that a physical literacy consensus statement will be developed and promoted. Further it states that a means to assess the implementation of this statement will be introduced (Department of Tourism, Culture, Arts, Gaeltacht, Sport and Media, 2021).  **Initiatives supporting the school ‘Wellbeing’ Agenda**  Physical activity promotion considerations are incorporated into a more general ‘wellbeing’ agenda. The Wellbeing Policy Statement and Framework for Practice (Department of Education and Skills 2019) asserts that student wellbeing is considered both an outcome and an enabler of other target outcomes. Schools are requested to include wellbeing promotion as a focus in School Self Evaluation. To support wellbeing support there are several support initiatives. The Health Service Executive’s (HSE’s) Education Programme supports teachers to “promote wellbeing, physical activity and healthy eating in school” and the National Education Psychological Service (NEPS) assists schools to promote wellbeing and (Health Service Executive, n.d.). |

| **E03**  **There are shared use agreements that utilise school spaces. Community access is supported by initiatives to promote and support opportunities for physical activity for all persons outside of normal school hours.** | |
| --- | --- |
| **Definitions and scope** | - Includes policies and PA standards to provide and promote the use of facilities (both public and private companies) for physical activity. - Includes partnerships with ‘private companies’. - Includes partnerships with voluntary and for-profit sport organizations and extends to non-government organisations (NGOs). |
| **International best practice examples (benchmarks)** | **US, North Carolina**  The US state of North Carolina has a lengthy history of promoting the use of school facilities by the community. NCGS § 115C-524 authorises community use of school facilities. NCGS § 115C-12(35) states that the state board of education shall encourage shared use of physical activity facilities (North Carolina Division of Public Health, 2014). |
| **Context e.g. EU action / regulation** | **WHO**  **Global Action Plan on Physical Activity**  The WHO’s GAPPA document recognises that access to recreational spaces and sports amenities is necessary by people of all ages and abilities to reduce inequalities. Action 2.4.1 recommends to member states to increase the availability of playing fields by encouraging a policy of shared use of school facilities (WHO, 2018). |
| **Evidence of implementation** | **Ireland**  **Community Sports and Physical Activity Hubs**  The Community Sport Hubs (CSH) initiative is a Sport Ireland initiative designed to increase local sport participation. The Community Sports and Physical Activity Hubs Evaluation Report (Sport Ireland, 2019) distinguishes between three types of CSH: community sports hubs, outdoor sports hubs, and importantly for this statement, school community sports hubs. School community sports hubs are characterised by using links with local schools to maximise sport facility usage.  For example: in Carrickphierish, Waterford a multipurpose sports hall is used by schools during the school day and rented by the local sport partnership on evenings and weekends (Sport Ireland, 2019).  **Participation Plan 2021-2024 Increasing Participation in a Changing Ireland**  Action 31 of the participation plan states that Sport Ireland will seek to promote access to school facilities through local collaboration and action 32 states that the sport section will be supported to access school infrastructure. This action includes provision of the necessary insurance cover (Sport Ireland 2021a).  **National Sports Policy 2018 – 2027**  Action 6 of the National Sports Policy (NSP) states that schools and sports clubs will be encouraged to cooperate to make better use of their facilities (DTTS, 2018). |

| **E04**  **National and/or sub-national policies are in place to promote and support safe active travel to and from school.** | |
| --- | --- |
| **Definitions and scope** | - Includes government-funded or managed services where the government is responsible for safe travel to and from school. - Includes private companies that are under contract by the government to provide safe travel to and from school. For the purpose of this indicator, ‘private companies’ includes for profit companies and extends to non-government organisations (NGOs). - Includes policies and guidelines to provide and promote safe active travel to and from school. - Includes the strategic placement of active travel infrastructure. - Includes the use of signage to highlight the benefits of active travel, as well as signage outlining safety. |
| **International best practice examples (benchmarks)** | **Japan**  Japan has a highly established “walking to school practice” that has been implemented since the School Education Act enforcement order, enacted in 1953, stating that public elementary schools should be sited within no more than 4 km, and for public junior high schools no more than 6 km from the student’s home. This policy is still successful today at promoting active transportation among Japanese children and youth.  Since 1953 Japan has a “walking to school practice” resulting from the implementation of the article 49 of the School Education Act, which regulates the siting of public schools in urban areas of Japan. This article establishes that the commuting distances are 4 km for elementary schools and 6 km for junior high schools. Based on these, the boards of education must ensure that children attend to schools located within those distances to allow children to walk to school (Mori et al., 2012; Tanaka et al., 2016). |
| **Context e.g. EU action / regulation** | **WHO**  **Global Action Plan on Physical Activity**  Action 3.1.3 of GAPPA requests WHO member states to “promote walk and cycle to school programmes” (WHO, 2018).  **WHOROE**  **Physical activity strategy for the WHO European Region 2016–2025**  One of the various measures listed under objective 2.2 ‘Promote physical activity in preschools and schools’ is the action that member states should provide for safe active commuting by bicycle or ‘walking bus’ (WHOROE, 2016).  **EU**  **2013 Council recommendations**  Indicator 17 of the EU HEPA monitoring network seeks information on whether EU member states have implemented national or sub-national schemes to promote active travel to travel (Council of the European Union, 2013). |
| **Evidence of implementation** | **Ireland**  **Smarter Travel: A Sustainable Transport Future. A New Transport Policy for Ireland 2009 - 2020**  Action 4 of the Smarter Travel Policy (STP) states that the government will require local authorities to retrofit neighbourhoods to ensure that walking and cycling are the most viable transport options for accessing local facilities. Shops and schools were cited as examples of such amenities.  Action 7 of STP proclaims that the government will ensure that every school has a travel plan to encourage alternatives to car use (Department of Transport, 2009).    **Programme for Government: Our Shared Future**  The programme for government states that the government will increase the number of children walking or cycling to school by mandating the Department of Transport to cooperate with schools, local authorities, the green schools programme and local initiatives (Department of An Taoiseach, 2020a).    **Green Schools – Travel Programme**   Supported by the Department of Transport and the NTA, the travel component of the Green Schools initiative (titled 'Safe Routes to School') encourages students to walk, cycle or use public transport to travel to school (An Taisce, 2021).  A review of implementation cites the actions taken as part of the Green Schools initiative as an implementation activity on action 7 of STP. (Department of Transport, Tourism and Sport, 2020).    **Participation Plan 2021-2024 Increasing Participation in a Changing Ireland**  Action 33 of the participation plan states that Sport Ireland and its partners will Identify and implement solutions to transport challenges that occur in accessing facilities after school (Sport Ireland, 2021a). |

DOMAIN 2 – Transport

**There are public policies to promote and support active mobility for people of all ages and abilities.**

| **T01**  **Regulations are in place that provide a variety of infrastructures to support safe walking and/or cycling and/or wheeling, including measures to calm speed, reduce vehicle traffic and enhance active mobility.** | |
| --- | --- |
| **Definitions and scope** | - Includes policies and guidelines to provide and promote safe active travel. - Includes the strategic placement of active travel infrastructure. - Includes government-funded or managed services where the government is responsible for the promotion of active travel. - Includes the use of signage to highlight the benefits of active travel, as well as signage outlining safety. |
| **International best practice examples (benchmarks)** | **Belgium, Brussels**  The city of Brussels in Belgium introduced a city wide 30km/h speed limit in January 2021. This makes the city of Brussels Europe’s largest 30km/h zone. Early results show a decrease in the speeds across Brussels roads (European Transport Safety Council, 2021). The measure is part of the “Good Move” Regional mobility plan which lists ‘promoting safe and healthy mobility practices’ amongst its seven challenges to be reconciled (Thiry, 2020). |
| **Context e.g. EU action / regulation** | **WHO**  **Global Action Plan on Physical Activity**  Action 2.2 of GAPPA requests WHO member states to “improve the level of service provided by walking and cycling network infrastructure, to enable and promote walking, cycling, other forms of mobility involving the use of wheels (including wheelchairs, scooters and skates) and the use of public transport, in urban, peri-urban and rural communities, with due regard for the principles of safe, universal and equitable access by people of all ages and abilities” (WHO, 2018).  **WHOROE**  **Physical activity strategy for the WHO European Region 2016–2025**  Objective 3.1 requests reduction of car traffic and increase walking and cycling suitability. National governments and local decision-makers should establish a mix of accessible walking and cycling infrastructures appropriate to national geographic and cultural contexts (WHOROE, 2016). |
| **Evidence of implementation** | **Ireland**  **Smarter Travel: A Sustainable Transport Future. A New Transport Policy for Ireland 2009 - 2020**  The Smarter Travel Policy (STP), with a timeframe of 2009 to 2020, contains 49 policy actions designed to affect a modal shift in transport away from cars (Department of Transport, 2009a).  The following are examples of actions included in STP.   - Action 11 states that the Government of Ireland will consider fiscal measures to reduce car use. A carbon tax was introduced in 2010 and has been increased in subsequent budgets (Department of Communications, Climate Action and Environment 2019). - Action 16 commits the Government of Ireland to increasing opportunities for walking, through a series of measures including providing safe pedestrian routes, reprioritising traffic signals to favour pedestrians, creating traffic-free areas in urban centres, and signposting pedestrian routes to important destinations (Department of Transport, 2009a). - Action 23 states that the Government of Ireland will ensure that cyclists and pedestrians are given road priority to key public transport interchanges (Department of Transport, 2009a).     **National Cycle Policy Framework**  The National Cycle Policy Framework (NCPF) was launched in 2009. It contains 19 objectives and 109 policy actions aimed at achieving these objectives (Department of Transport 2009b). Nine of these objectives and 53 actions were developed specifically to ensuring adequate physical infrastructure for cyclists. The nine infrastructure-related objectives were:   - Support the planning, development and design of towns and cities in a cycling and pedestrian friendly way. - Ensure that the urban road infrastructure is designed or retrofitted so as to be cyclist-friendly and that traffic management measures are also cyclist friendly. - Provide designated rural cycle networks especially for visitors and recreational cycling. - Provide cycling-friendly routes to all schools, adequate cycling parking facilities within schools, and cycling training to all school pupils. - Ensure that all surfaces used by cyclists are maintained to a high standard and are well lit. - Ensure that all cycling networks - both urban and rural - are signposted to an agreed standard. - Provide secure parking for bikes. - Ensure proper integration between cycling and public transport. - Provide public bikes in cities.     Two objectives and nine policy actions were developed to enforce and legislate for increased cycle safety. The two legislation and enforcement actions were:   - Introduce changes to legislation to improve cyclist safety. - Improve enforcement of traffic laws to enhance cyclist safety and respect for cyclists.     **Speed limits**  The Road Safety Act of 2004 legislates a number of ‘ordinary speed limits’: 50 kilometres per hour (km/h) for roads in built up areas, 80 km/h in regional and local roads other than those in built up areas, 100 km/h on national roads and 120km/h on motorways. The same act empowers local authorities (i.e. city and county councils) to designate particular roads as having a ‘special speed limit’ (Road Traffic Act, 2004).    In 2015, the Government department with responsibility for transport issued guidelines for the setting and managing of these special speed limits by city and county councils. Maps of roads where special speed limits are in effect are uploaded by city and county councils to the website speedlimits.ie.  A 2021 city council plan to make 30km/h the default speed limit in Dublin city was dropped after a public consultation revealed 46% of respondents were against the measure with 41% in favour (Kilraine, 2021).  **Review of Sustainable Mobility Policy**  In late 2019 and 2020 the Government of Ireland undertook a public consultation to investigate the status of Ireland’s sustainable mobility (Department of Transport Tourism and Sport, 2020). Reviews of STP and NCPF were published as part of this consultation.  The review on the implementation status of STP utilised a traffic light signal to signify the level of progress in implementing the policy actions: green for substantive progress or on-going implementation, amber for some substantive progress, red for minimal or no substantive progress. The report presents 18 green ratings, 34 amber ratings, and 6 red ratings. Some policy actions of STP were split into several subcomponents, hence the sum of the number of ratings is greater than the number of actions.    The report on NCPF implementation utilised the same system. Thirty-seven actions received green ratings, 37 received amber ratings, 35 received red ratings (Department of Transport Tourism and Sport, 2020). |

| **T02**  **There is a funded implementation plan, led by the appropriate level/s of government, to achieve improvements in active travel and increased use of public transport.** | |
| --- | --- |
| **Definitions and scope** | - Includes plans for the strategic placement of active travel infrastructure. - Includes plans for the use of signage to highlight the benefits of active travel, as well as signage outlining safety. |
| **International best practice examples (benchmarks)** | **Belgium, Ghent**  The city of Ghent, Belgium implemented a new circulation plan in 2017. The goal of the plan was to reduce traffic in the city centre. This was achieved by dividing the city into a pedestrian zone in the centre and six surrounding sections (Hellemeier and Soltaniehha 2010). Private motorized transport was not permitted in the city centre and traffic moving from one of the six zones to another was redirected to the ring road (De Geest, 2017). This arrangement increases the barriers to private motorized transport for short journeys within the city and facilitates alternative modes of transportation. Citizens report that the city is now much easier to traverse by these alternative modes of transportation (Heath and Bilderback 2019). The effect was a mode shift to walking, cycling and public transportation (Zając, 2019).  **Italy, Pasero**  Towards new Regional Action plans for sustainable urban Mobility (TRAM) is designed to strengthen the urban dimension of regional and local policymaking, contributing to the implementation of EU Transport White Paper, Urban Agenda and the EU 2020 strategy and facilitating the shift to low carbon economy (Reid, 2020). The TRAM was applied in Pasero City where the bicycle routes were extension to 87 km in 2017 and the “zones 30” were used. Nowadays Pesaro is one of the most Bike Friendly City in Italy, with the highest modal share satisfied by bike (Thiry, 2020).  **US, Chattanooga County**  Grow Healthy Together Chattanooga (GHTC) was a community project funded by the Robert Wood Johnson Foundation’s (RWJF) Healthy Kids, Healthy Communities initiative. The goal of the project was to increase the number of parks/recreational sites, length of added sidewalks and length of designated bicycle lanes in each community, and also to build new bus shelters. Several government agencies (Chattanooga/Hamilton County, Health Department, Hamilton County Schools, City Parks and Recreation, Chattanooga Area Regional Transportation Authority (CARTA), were involved in the planning and implementation of the project. Analysis of the results showed that improved access to “urban” pedestrian/bicycle routes/trails appears to translate into increased opportunities for physical activity among inner-city children/youth (Interreg Europe, 2021).  **United Kingdom, Wales**  The Active Travel (Wales) Act 2013 makes it a legal requirement for local authorities in Wales to map and plan for suitable routes for active travel, and to build and improve every year their infrastructure for walking and cycling as a mode of transport. The health dimension in transport planning is a driving force of the Act to achieve wider national objectives related to well-being, physical activity, behaviour change and road safety. The Act makes explicit the essential role of good quality and integrated infrastructure networks to encourage active travel, and the need to incorporate the health dimension in legislation, standards and tools of governing bodies beyond the transport sphere.    **Sweden, Gothenburg**  Gothenburg has worked systematically to improve accessibility of public transport for people with reduced mobility in the frame of the KOLLA project (“Public transport for all”). This includes: adaptation of the vehicles (buses and trams), improving accessibility to walking paths leading to public transport stops and areas in direct connection to the stops. Over the course of the five-year project, 6,500 obstacles to accessibility were inventoried to enable systematic removal by 2018. After introducing the Flexline bus service for people with reduced mobility, the number of special transport services dropped by 180,000 trips. The KOLLA project was the winner of the European Access City Award 2014. Project KOLLA was a collaboration between the regional public transport authority (Västtrafik), and the departments covering special transport services (Färdtjänstförvaltningen) and traffic and roads (Trafikkontoret) in the City of Gothenburg. The project involved close cooperation with a reference group - a User Council formed specifically for the KOLLA project. The User Council consisted of thirteen representatives from seven different user organisations representing people with different types of reduced mobility (seeing, hearing, function, elderly etc). |
| **Context e.g. EU action / regulation** | **WHO**  **Global Action Plan on Physical Activity**  Action 2.3 of GAPPA requests WHO member states to “accelerate implementation of policy actions to improve road safety and the personal safety of pedestrians, cyclists, people engaged in other forms of mobility involving the use of wheels (including wheelchairs, scooters and skates) and public transport passengers (WHO, 2018). |
| **Evidence of implementation** | **Ireland**  **Smarter Travel: A Sustainable Transport Future. A New Transport Policy for Ireland 2009 - 2020**  STP recognised the trends towards greater car ownership and greater car dependency. To achieve greater sustainability in Irish transport it sought to affect a mode shift towards more active travel and public transportation. It contained the following targets (amongst others):   - Reduce the share of commuters travelling by car from 65% to 45% and increase the share of commuters walking, cycling or using public transport to 55%. - Keep the number of kilometres travelled by car at current [2009] levels.     To achieve its ‘smarter travel’ targets the STP set out 49 actions. Programmes established by these actions are ongoing. Actions in STP designed to promote active travel include the following:   - Actions 7 and 8 deal with school and workplace travel programmes which are dealt with elsewhere in this document. - Actions 12 to 14 are designed to shift commuters from cars to public transport. - Actions 15 to 17 are designed to promote walking and cycling.     The policy was estimated to require €4.5 billion during its lifetime to achieve its goals. Funding decisions were described as a matter for the government “in light of prevailing economic and budgetary parameters” (Department of Transport, 2009, p62).    **Climate Action Plan (2019)**  The Climate Action Plan published in 2019 (CAP19) contains 183 actions.   - Action 91 of the Climate Action Plan commits the Government of Ireland to establish a Cycling Project Office within the National Transport Authority (NTA). This office will expand the cycling infrastructure and develop an implementation plan. - Action 95 commits the Government of Ireland to publishing an implementation plan building on the Smarter Travel Policy (STP) and addressing any outstanding policy commitments. - Action 97 states that the Government will commence full implementation of the National Cycle Policy Framework (NCPF) (Department of the Environment, Climate and Communications, 2019).     **Programme for Government: Our Shared Future**  The 2020 Programme for Government states that €360 million will be allocated to improving cycling and pedestrian infrastructure over the lifetime of the government (Department of An Taoiseach, 2020). Our Shared Future also states that local authorities will be mandated to adopt ‘high-quality’ transport policies with the assistance of trained Cycling Officers. Furthermore, the expenditure on public transport infrastructure will be great than the expenditure on new roads by a ratio of 2:1 (Department of an Taoiseach, 2020a).  **Climate Action Plan (2021)**  A new Climate Action Plan (CAP21) was published in 2021, building on the 2019 plan. According to the new plan €125 billion in investment is required to deliver on all the actions over the course of the plan.  A target listed in the Climate Action Plan (2021) is that an additional 500 000 public transport and active travel journeys will be undertaken per day versus 2021 levels by 2030.  The action plan contains 475 actions   - Action 223 of the Climate Action Plan commits the Government of Ireland to the improvement and expansion of the Active Travel and Greenway Network - Action 224 of the Climate Action Plan commits the Government of Ireland to the development of a coherent and connected National Cycle Network Strategy - Action 225 of the Climate Action Plan commits the Government of Ireland to the construction of an additional 1,000km of cycling and walking infrastructure - Action 226 of the Climate Action Plan commits the Government of Ireland to increasing the level of modal shift towards Active Travel (walking and cycling) and away from private car use. - Action 230 of the Climate Action Plan commits the Government of Ireland to legislate to improve the Active Travel environment in urban centres - Action 232 of the Climate Action Plan commits the Government of Ireland to publish the new ten-year Sustainable Mobility Policy (Department of the Environment, Climate and Communications, 2021). |

| **T03**  **Guidelines and tools to support infrastructure for active mobility and/or transport plans and systems that encourage physical activity are promoted and disseminated.** | |
| --- | --- |
| **Definitions and scope** | - Includes policies that influence change of people behaviour by different mechanisms to enhance their shift from motor-power transport to active transportation. |
| **International best practice examples (benchmarks)** | **Scotland**  The Smarter Choices, Smarter Places (SCSP) programme implemented in Scotland, intended to encourage uptake of walking, cycling and the use of public transport as more active forms of travel. To achieve these goals, local authorities covering the participating towns delivered complex programmes to organise, enable, promote and provide sustainable transport solutions. Implementation of SCSP was restricted to seven local areas. The interventions were many interventions incl.:  *public transport provision - new bus services, ticketing improvements, bus shelters and other improvements at bus stop,  *infrastructure provision - new cycleways, footpaths, pedestrian crossings, bus lanes, pedestrianisation, cycle facilities, and other associated changes to the built environment,  *active travel promotion - covering a wide range of different general promotional activities covering health walks, healthy lifestyles, calorie maps, and other health promoting materials and activities (Norwood *et al.,* 2014).  **Belgium, Flanders**  The international PA guideline (10000 steps/day) has been implemented as “10 000 Steps” programme in the entire state of Flanders (Dutch) in 2007. The aim of a multistrategy community-based intervention was to increase PA. The project was focused on promoting PA by local media campaign, community events and selling and loaning pedometers etc (Dubuy et al. 2013).  **United Kingdom, London**  Transport for London (TfL) has been implementing the Transport Action Plan between 2014 and 2017. They did it in partnership with Greater London’s 32 boroughs (local authority districts) and the departments in London’s city authorities responsible for urban planning and health. It provides a strategic approach to active travel promotion through the Healthy Streets Approach, which itself is based on 10 Healthy Street Indicators. The Plan has helped drive a shift towards active transport modes, especially in planning and policy. In this way, it has provided a framework for considering ways to increase everyday physical activity levels amongst London’s population, a large proportion of whom are inactive (27%) (Ref. The EU-funded project PASTA - Physical Activity Through Sustainable Transport Approaches - aimed to connect transport and health by promoting active mobility in cities).  As part of the local Go Cycle programme, in September 2016 the London Borough of Kingston-upon-Thames piloted a new cycling on referral scheme to encourage those who live, work and study in Kingston to take up cycling to improve their health. Doctors and a range of specialist health professionals could refer patients suffering from health problems connected to lack of physical activity to professional cycling trainers to help them better manage and improve their health. The 12-week programme is free of charge and includes a number of fixed appointments with professional cycle coaches. |
| **Context e.g. EU action / regulation** | **WHO**  **Global Action Plan on Physical Activity**  Action 2.2 of GAPPA requests WHO member states to “improve the level of service provided by walking and cycling network infrastructure, to promote walking, cycling, other forms of mobility involving the use of wheels (including wheelchairs, scooters and skates) and the use of public transport, in urban, peri-urban and rural communities, with due regard for the principles of safe, universal and equitable access by people of all ages and abilities” (WHO, 2018).  **Physical activity strategy for the WHO European Region 2016–2020**  Objective 3.1 requests reduction of car traffic and increase walking and cycling suitability. National governments and local decision-makers should promote human-powered transport (WHOROE, 2016). |
| **Evidence of implementation** | **Ireland**  **National Cycle Manual**  The National Cycle Manual (2011), published by the National Transport Authority aims to encourage more people to cycle for transport by improving the ‘cycling offer’. The manual is a 226-page document with seven sections (The Basics, Legislation and Policy, Planning for the Bicycle, Designing for the Bicycle, Getting the Details Right, Maintenance, Tools and Checklists)  for planners on how to incorporate cycling into transport systems (National Transport Authority, 2011). The working group for the draft manual included representatives from Kildare, Laois and South Dublin county councils, Limerick and Dublin city councils, the Dublin Transportation Office, the Quality Bus Network Project Office, the National Roads Authority and An Garda Síochana. It is available at the website: www.cyclemanual.ie.    **Design Manual for Urban Roads and Street**  The Design Manual for Urban Roads and Street (2013), prepared by the former Department of Transport, Tourism and Sport and the former Department of Environment, Community and Local Government, provides advice on prioritising vulnerable road users (i.e. pedestrians and cyclists) (Department of Transport, Tourism and Sport and Department of Environment, Community and Local Government, 2013). |

DOMAIN 3 – Urban Design

**There are public policies enacted at appropriate level/s of government to ensure that evidence-informed urban design principles are implemented to promote and support physical activity and active mobility for people of all ages and abilities.**

| **UD01**  **Policies or regulations that take a “health in all” approach is adopted to reallocate space from motorised transport to active travel and/or recreation purposes.** | |
| --- | --- |
| **Definitions and scope** | - Includes policies and guidelines that promote a holistic approach to infrastructure planning and mixed-land use. - Includes the strategic placement of active travel infrastructure. - Includes the use of signage to highlight the benefits of active travel, as well as signage outlining safety. |
| **International best practice examples (benchmarks)** | **Netherlands, Groningen**  The city of Groningen has the highest use of bicycles as a mode of transport of any city in the Netherlands (Ashworth 2001). Its high use of bicycles has led residents to self-style as the ‘bicycle capital of Europe’ (Pucher and Buelher 2007). In 1977, Groningen introduced the traffic circulation plan (VCP) which dramatically reduced the number of cars in the inner city (Tsubohara, 2007). The VCP divided the city into four sections and required motorists to use the ring road to move between sections while cyclists retained the right to move freely between the sections (Van Der Zee, 2015).  **Spain, Pontevedra**  The city of Pontevedra in Spain banned all nonessential car journeys in the city centre in 1999. These policies have had ancillary positive effects. Between 1999 and 2014 CO^2^ emissions were reduced by 66% and smaller shops in were more resilient to economic crises than those in other comparable cities in the country (Hardinghaus et al., 2021). |
| **Context e.g. EU action / regulation** | **WHO**  **Global Action Plan on Physical Activity**  Action 2.1: Strengthen the integration of urban and transport planning policies that prioritize the principles of compact, mixed land use, at all levels of government as appropriate, to deliver highly connected neighbourhoods that enable and promote walking, cycling, other forms of mobility involving the use of wheels (including wheelchairs, scooters and skates) and the use of public transport, in urban, peri-urban and rural communities (WHO, 2018).  Proposed sub action for member states 2.1.3: Support the development and implementation of planning and transport policy, guidelines and regulations that redistribute, as appropriate, urban space from private motorized transport to support increased walking, cycling and use of public transport, as well as provision of public open and green spaces, including regulations to limit car parking options for singular occupancy private vehicles. |
| **Evidence of implementation** | **Ireland**  **Get Ireland Active! National Physical Activity Plan for Ireland** The National Physical Activity Policy (NPAP) contains the following actions:  Action 33: "Ensure that the planning, development and design of towns and cities promotes cycling and walking with the aim of delivering a network of cycle routes and footpaths" (Healthy Ireland, 2016 p38).  Action 34: "Ensure that the planning, development and design of towns and cities promotes the development of local and regional parks and recreational spaces that encourage physical activity" (Healthy Ireland, 2016 p38).  Action 36: "Prioritise the planning and development of walking and cycling and general recreational / physical activity infrastructure" (Healthy Ireland, 2016 p38).    **Project Ireland 2040 National Planning Framework**  Project 2040 the National Planning Framework is a policy document which lists 75 national policy objectives designed to manage future growth and development. National policy objective 26 states that the public health objectives of the Healthy Ireland Framework and the National Physical Activity Plan (NPAP) will be integrated into planning policy.    **Smarter Travel: A Sustainable Transport Future. A New Transport Policy for Ireland 2009 - 2020**  Action 12 of STP states that the Government of Ireland will implement “more radical bus priority measures” and that this may include “making some urban streets car-free”.  Action 17 commits the Government of Ireland to make state-owned lands, such as former rail lines, available for walking and cycling trails. |

| **UD02**  **Governments adopt land use policies, and planning processes, consistent with principles of mixed land use, compact urban design, and/or provision of green open spaces to support physical activity and reduce motorised transport.** | |
| --- | --- |
| **Definitions and scope** | - Appropriate consultation processes are in place to encourage active participation and engagement of community members and transparent mechanisms exist for use of consultations to inform policy development. |
| **International best practice examples (benchmarks)** | **New Zealand, Auckland**  In 2018 Auckland Plan, Strategy and Research Department published Auckland Plan 2050 Evidence Report. This plan is focused on the sustainable development, safe and friendly environment and increasing active transport. Chapter 5 describes how the elements of this report were consulted with partners, stakeholders and the public (Auckland Council, 2018). |
| **Context e.g. EU action / regulation** | **WHO**  **Global Action Plan on Physical Activity**  Proposed sub action for member states 2.4.1: Promote and enforce urban planning, land use and spatial policy at all levels of government, as appropriate, that requires the provision of a connected network of green infrastructure that enables equitable access to quality, safe public space, blue space and green open spaces, natural spaces, recreational areas and sports facilities (WHO, 2018). |
| **Evidence of implementation** | **Ireland**  **Project Ireland 2040 National Planning Framework**  National policy objective 27 denotes that the Government of Ireland will integrate alternatives to car transport into the design of communities, prioritise walking and cycling and integrate physical activity facilities for all. |

| **UD03**  **There are guidelines and/or regulations that improve universal and equitable access to safe outdoor and indoor spaces and facilities where people can be physically active.** | |
| --- | --- |
| **Definitions and scope** | - Includes policies and guidelines to provide and improve access to quality open spaces. - Includes the strategic placement of quality open spaces. - Includes policies and regulations to ensure universal access to buildings by all people. - Frameworks, strategies, or implementation plans specify aims, objectives or targets to reduce inequalities including taking a preventive approach that addresses the social and environmental determinants of health. - Frameworks, strategies, or implementation plans identify vulnerable populations or priority groups. - Implementation plans specify policies or programmes that aim to reduce inequalities for specific population groups. |
| **International best practice examples (benchmarks)** | **China, Ürümqi**  Implementation of the “Opinions on the Construction of a Garden City in Urumqi” (2001) plays an important role in the improvement of the urban green space (UGS) area and the accessibility of built-up areas. The amount of UGS in the built-up areas increased rapidly from 1999 to 2019, reflecting the fairness of UGS in the overall amount. However, the distribution and different UGS categories are shows the spatial imbalance of UGS resources and need to be improved (Shi et al. 2020). |
| **Context e.g. EU action / regulation** | **WHO**  **Global Action Plan on Physical Activity**  Action 2.4: Strengthen access to good-quality public and green open spaces, green networks, recreational spaces (including river and coastal areas) and sports amenities by all people, of all ages, and of diverse abilities in urban, peri-urban and rural communities, ensuring design is consistent with these principles of safe, universal, age-friendly and equitable access with a priority being to reduce inequalities (WHO, 2018). |
| **Evidence of implementation** | **Ireland**  **Design Manual for Urban Roads and Streets**  The Design Manual for Urban Roads and Streets (DMURS) provides guidance for urban street design. It is mandatory that local authorities consider the DMURS in their urban planning. The DMURS encourages designers to consider a ‘user hierarchy with pedestrians and cyclists at the top public transport below and private motor vehicles at the bottom. The DMURS and related documents are available online on the dedicated website: <https://www.dmurs.ie/>.  **National Cycle Framework Policy**  The National Cycle Framework Policy (NCPF) lists as an objective (objective 1) that planning will be cyclist and pedestrian friendly. Action 1.1 of NCPF states that all planning guidelines will support cycling promotion as a stated objective (Department of Transport, 2009b).    **The Permeability Best Practice Guide**  The Permeability Best Practice Guide prepared by the NTA in collaboration with South Dublin County Council provides guidance on increasing permeability in built-up areas. Permeability as defined by the guide is “the extent to which an urban area permits the movement of people by walking or cycling” (as opposed to motorised vehicles).  The guide sets out principles for achieving permeability (National Transport Authority, 2015).    **Ready, Steady, Play! A National Play Policy**  The National Play Policy was developed to ensure that all Irish children have opportunities to participate in play. Emphasis is placed on ensuring that children have access to playgrounds and recreational facilities. An aim of the policy is to ensure that all playgrounds provided by public authorities conform to standards specified in the Irish Standards for play equipment and surfacing. Another aim of the policy is to bring the supply of playgrounds up to 1 per 10 000 population. It is estimated in the policy that the shortfall in provision at the time of policy’s preparation would require building 204 local playgrounds at a minimum cost of €16 million (National Children’s Office, 2004 p.57).    **Better Outcomes, Brighter Futures, the national policy framework for children & young people 2014 - 2020**  Commitment number 1.15 of the national policy framework for children and young people commits government to creating more recreational spaces for children and young people. Examples of such spaces that are provided include youth cafés, playgrounds and sports and leisure centres (Department of Children, Equality, Disability, Integration and Youth, 2019). |

DOMAIN 4 – Healthcare

**Public policies implemented within healthcare settings promote and support physical activity, e.g., by providing guidelines and regulations, applying digital health technologies, and targeting at-risk groups like older adults.**

| **H01**  **Guidelines and regulations in healthcare include routine screening for physical activity and, for all insufficiently active patients, brief advice, and referral to appropriately trained practitioners and/or physical activity opportunities.** | |
| --- | --- |
| **Definitions and scope** | - Ensures policies and guidelines are in place that encourage and promote PA in the rehabilitation pathways for patients. - Excludes pre-and in-service training on the importance of physical activity promotion within healthcare (which is covered under Workforce Development 03). |
| **International best practice examples (benchmarks)** | **United Kingdom**  The National Institute for Health and Care Excellence (NICE) provides brief advice guidelines for professionals and practitioners in primary care. (NICE project team, 2013).  **Belgium**  **Physical activity on referral (Bewegen Op Verwijzing)** The programme aims to increase physical activity levels during subsidized sessions with personal physical activity coaches. Its main instrument is a written referral by primary care physicians to inactive and/or sedentary people, with a focus on disadvantaged groups. The programme is coordinated by the Flemish Institute of Healthy Living, funded and supported by the Flemish government and implemented by an intersectoral network of local organizations (WHO/Europe, 2018).  **Croatia**  **Walking towards health (Hodanjem do zdravlja)**  Initiated by the National Health Promotion Programme “Healthy living”, health professionals inform and encourage citizens to participate in regularly organized walks with professional guidance as part of the programme “Walking towards health”. This programme has been implemented in 21 counties by multidisciplinary teams of experts, including general physicians, hospital representatives and city representatives. The next step is to adapt walking routes in all counties to turn them into designated health-promoting environments (WHO/Europe, 2018).  **Czech Republic**  **Short intervention web (krate intervence)**  The programme provides for short interventions by professionals who are trained by the National Institute of Health in the “short intervention method”, which teaches communication and motivation. The method is suitable for workers in health care, social services and education who are interested in educating ther patients, clients or pupils about risk factors and health-enhancing lifestyle behaviour, including physical activity. The programme is supported by the National Health Programme of the Ministry of Health (WHO/Europe, 2018).  **Finland**  **Lifestyle counselling for social and health care through a multisectoral network (VESOTE)**  The programme is designed to reinforce and develop lifestyle counselling in social and health care, to increase physical activity, decrease sedentary behaviour, improve diet and sleeping patterns and reduce health inequalities. It is funded by the Ministry of Social Affairs and Health and covers 4 million people in 184 municipalities. In particular, VESOTE includes adopting and reinforcing lifestyle counselling models, capacity-building for lifestyle counselling and using and extending good practices. The programme offers activities such as web-based training for professionals, e-lectures, development and improvement of treatment and service chains among different units in health care and objective population-based monitoring of physical activity and sleeh (WHO/Europe, 2018). |
| **Context** | **WHO Global Action Plan on Physical Activity:**  Implement and strengthen systems of patient assessment and counselling on increasing physical activity and reducing sedentary behaviour, by appropriately trained health, community and social care providers, as appropriate, in primary and secondary health care and social services, as part of universal health care, ensuring community and patient involvement and coordinated links with community resources, where appropriate (action 3.2) (WHO, 2018).  **Physical activity strategy for the WHO European Region:**  Member States should work towards making the promotion of physical activity by health professionals the norm. Early identification, counselling and referral at the primary care level should be integrated into standard practice and should respond to the different needs of patients. For the general population, a simple assessment of the level of physical activity could be integrated into the ongoing risk factor assessment, followed by brief advice, if required, about recommended levels. For patients requiring more support, the counselling may take the form of an intervention using motivational techniques and goal setting, with referral to specialists and other health and allied professionals, where necessary. While the promotion of physical activity should be seen as a core competence for all primary health-care professionals, governments could also consider ways to continuously provide incentives for its full integration into daily practice. Physical activity programmes or sports classes and physical activity-based prevention or rehabilitation offers may be considered, according to national circumstances, for the purpose of reimbursement by health insurance companies or national health systems when and where appropriate, with clear guidance for health professionals (objective 3.3) (WHOROE, 2016).  **EU**  **2013 Council Recommendations:**  Counselling on physical activity performed by health professionals (indicator 11) (Council of the European Union, 2013). |
| **Evidence of implementation** | **Ireland**  **National Exercise Referral Framework (NERF)**  The NERF is a framework for embedding physical activity promotion into the healthcare setting, particularly for individuals living with an established NCD or mental illness. Healthcare professionals, physical activity service providers and the participants themselves can work together to fully realize Healthy Ireland’s potential to make Ireland a more active place to live.  NERF proposes that a fourth stakeholder group the Exercise Referral Unit be established to manage the implementation of the framework. NERF presents a five-stage model (the Physical Activity Pathways in Healthcare Model) detailing how patients progress through the process of exercise referral. (Woods et al., 2016).  **Self-Management Support Framework**  The Self-Management Support Framework (SMSF) contains 44 recommendations for patients managing chronic conditions.  Recommendation 4 states that exercise-based programmes and supports for disease self-management, such as cardiac and pulmonary rehabilitation, should be standardised nationally (Mullaney et al., 2017, p10). Furthermore recommendation 11 states that referral to behaviour change interventions, including exercise interventions should be available to patients through contact with regular healthcare professionals.  The framework also includes a list of Implementation actions to be carried out by various healthcare partners. Actions 7.19 (a) and 7.19 (c) relate to resourcing, scaling and standardising cardiac and pulmonary rehabilitation, respectively.  **Get Ireland Active! National Physical Activity Plan for Ireland**  The following actions of the National Physical Activity Plan (NPAP) deal with screening and referral in the healthcare system  Action 23: "Develop and implement a brief intervention model for delivery of physical activity advice" (Healthy Ireland, 2016 p37).  Action 25: "Develop and implement a pilot programme for the National Exercise Referral Framework" (Healthy Ireland, 2016 p37).  Action 26: "Include questions on physical activity levels in risk factor screening and brief intervention/patient education" (Healthy Ireland, 2016 p37).  **Exercise Referral for the Health Services Briefing Document**  In 2017, the HSE’s Healthy Eating and Active Living Programme conducted an assessment of the structures required to successful implementation action 25 of NPAP (above). The assessment recommended that certain structures needed to be put in place to order for referral to be successful. Among these recommendations were that staff training via Making Every Contact Count (MECC, see below) needed to be rolled out prior to any pilot exercise referral scheme. It also recommended that the exercise referral pathway should be into the pathways of SMSF.  **Making Every Contact Count**  The implementation plan, Making Every Contact Count (MECC), provides a model for promoting health behaviours including physical activity to persons who access health services. The model resembles a pyramid. The lowest tier of the pyramid recommends providing brief advice to everyone accessing the health system.  Higher tiers include a recommendations to provide brief interventions for persons with diagnosed lifestyle risk factors and specialist services for those who require the greatest level of support (HSE, 2021).  The eLearning training programme consists of 6 x 30-minute eLearning modules.    Cunningham and O’Sullivan (2021) conducted a survey of healthcare professional’s knowledge of PA promotion. Based on 347 eligible responses, 30% of healthcare professionals agree that they have received “suitable training to initiate conversations with patients about physical activity” (p1). |

| **H02**  **There are consistent policies for promoting and supporting physical activity in primary and secondary healthcare settings among at-risk groups, such as people with type 2 diabetes and older adults (e.g., protocols for the assessment of the physical activity capacity; accessible, affordable, and tailored physical activity programmes; and training for caregivers for delivering physical activity programmes within residential aged care).** | |
| --- | --- |
| **Definitions and scope** | - Includes policies that relate to healthy ageing programmes, where the programme is partly or fully funded, managed or overseen by the government. |
| **International best practice examples (benchmarks)** | **France**  In 2016, prescription of sport for persons with long-term illness was described in legislation. Article D1172-2 of the public health code (“Code de la santé publique”) states that a physician may refer a patient to a health professional for sport if the health professional holds the appropriate qualifications (Government of the French Republic, 2016; Sport-ordonnance.fr, 2017).  **Adapted Physical Activity**  The programme aims to enable people with chronic diseases to have a physically active lifestyle in order to reduce their risk factors and the functional limitations due to their conditions. A “sport on prescription” scheme is implemented by several local authorities using different approaches. The patient’s needs in terms of physical activity are usually identified by a general practitioner who prescribes it as part of care, referring the beneficiary to local sports associations that offer sport-health programmes. Interviews and physical tests are conducted to design a personalized programme adapted to the individual. After the programme, personalized follow-up and support are offered (WHO/Europe, 2018). |
| **Context e.g. EU action / regulation** | **EU**  **Physical activity strategy for the WHO European Region:**  Member States should ensure that health professionals are in a position to provide simple and timely advice to older patients on the specific benefits of a physically active lifestyle that is tailored to their individual health needs, capacity and preferences; where needed, more in-depth counselling and support for change should be offered. They should also be able to provide linkages to tailored community services and resources to support physical activity among older people (objective 4.1) (WHOROE, 2016).  **WHO Global Action Plan on Physical Activity:**  Enhance the provision of, and opportunities for, appropriately tailored programmes and services aimed at increasing physical activity and reducing sedentary behaviour in older adults, according to ability, in key settings such as local and community venues, health, social and long-term care settings, assisted living facilities and family environments, to support healthy ageing (action 3.4) (WHO, 2018). |
| **Evidence of implementation** | **Ireland**  **Sláintecare Healthcare Reform**  In 2017, Sláintecare, a major reform of healthcare in Ireland was announced. The first of ten key recommendations of the report drafted by legislatures was to expand Health and Wellbeing, in essence the services that support people to protect their own health, to achieve a better integrated system (Houses of the Oireachtas, 2017).  **Sláintecare Integration Fund**  The Sláintecare Integration Fund was established to support and evaluate innovative services with a focus on “prevention, community care and integration of all health and social care settings” (Department of Health, 2020 p7). Projects can transition from the integration fund to mainstream funding by providing proof of concept (Government of Ireland, 2021). These supported projects can included physical activity interventions. For example round one of the Integration Fund supported a project called Active Neuro designed to deliver physical activity programmes to people with neurological conditions in the community (Department of Health, 2020).  **Get Ireland Active! National Physical Activity Plan for Ireland** The National Physical Activity Plan (NPAP) contains the following actions to promote physical activity in healthcare settings:   - Action 29: "Develop guidelines, support materials and referral pathways to promote physical activity for organisations providing mental health services and disability services" (Healthy Ireland, 2016 p35). - Action 30: "Develop guidelines to integrate physical activity into long-term care planning and practice" (Healthy Ireland, 2016 p35).     **Let's Get Active!... to Improve Health and Wellbeing** The Let's Get Active! document was prepared to fulfil action 29 of NPAP by the HSE (Healthy Ireland et al., 2021). Let's Get Active! is divided into the following chapters and appendices:    Chapter 1: What do we mean by physical activity?  Chapter 2: What are the benefits of physical activity?  Chapter 3: How much physical activity is advised?  Chapter 4: How can staff support service users to get more active?  Chapter 5: Practical tips and examples for increasing physical activity  Chapter 6: Safety advice  Chapter 7: Conclusion  Appendix 1: PAR-Q Screening Tool  Appendix 2: Get Ireland Active Leaflet and Physical Activity Diary  Appendix 3: Further exercise resources (Broderick et al., 2018).  **National Exercise Referral Framework (NERF)**  The NERF details how physical activity promotion can be embedded into the treatment pathways of individuals living with an established NCD or mental illness. NERF presents a five-stage model (the Physical Activity Pathways in Healthcare Model) detailing how patients progress through the process of exercise referral (Woods et al., 2016).  **Age & Opportunity – CarePALs** Age & Opportunity Active is a national organisation seeking to increase the quality of life of Irish people as they age. Age and Opportunity train 'physical activity leaders', known as CarePALs to deliver physical activity in residential and day care institutions (Age & Opportunity, 2022). The CarePALs peer leadership programme has been cited in NPAP implementation reports as activities fulfilling action 30 (Healthy Ireland et al., 2021). |

DOMAIN 5 – Mass Media / Public Education

**There are national and/or subnational public policies implemented to ensure enactment of media/education campaigns that actively promote and support increasing physical activity levels for all ages and abilities.**

| **MM01**  **There are national and/or subnational public policies in place that ensure media and education campaigns that promote and support physical activity are sustained and monitored (e.g., by making them part of, or aligning them with, a national action plan on physical activity and the physical activity guidelines).** | |
| --- | --- |
| **Definitions and scope** | - Includes mandatory policy (i.e. legislation or regulations) or voluntary standards, codes, guidelines set by government or by industry where the government plays a role in promoting PA and associated benefits. - Includes free-to-air and subscription television and radio only. |
| **International best practice examples (benchmarks)** | **Netherlands**  Rijksoverheid (former Postbus 51) creates and facilitates educational campaigns, including tv advertisments, posters, radio. Educational campaigns around covid measures, drunk driving (BOB), offline while driving/biking (MONO), no alcohol and smoking under 18 (NIX18). The effects and awareness of campaigns are monitored and published on government website.  MPOWER policy package to reverse the tobacco epidemic (WHO, 2008). |
| **Context e.g. EU action / regulation** | **WHO**  **Global Action Plan on Physical Activity**  Action 1.1: Implement best practice communication campaigns, linked with community-based programmes, to heighten awareness, knowledge and understanding of, and appreciation for, the multiple health benefits of regular physical activity and less sedentary behaviour, according to ability, for individual, family and community well-being.  Proposed sub action for member states 1.1.1: Develop a national communication strategy for physical activity as part of, or aligned with, a national action plan on physical activity to raise awareness and knowledge of the health benefits of physical activity, promote behaviour change and increase health and physical literacy (WHO, 2018).  **EU**  **2013 Council recommendations**  Indicator 23: Existence of a national awareness raising campaign on PA. |
| **Evidence of implementation** | **Ireland**  **Healthy Ireland, A Framework for Improved Health and Wellbeing 2013- 2025**  Action 2.9 of the Healthy Ireland Framework states that the Department of Health, the HSE, local authorities and statutory agencies will work together to implement a communications plan to promote public health (including physical activity). The 'Healthy Ireland' brand is to be applied to programmes and actions across sectors (Healthy Ireland, 2013).    **Healthy Ireland Communication and Citizen Engagement Campaign**  A communications campaign was launched in January 2018 focussing on healthy eating, physical activity and mental wellbeing (KildareStreet, 2018; Oireachtas.ie, 2020).    **Feel Good Together Campaign**  In June 2018 the Healthy Ireland summer campaign ‘Feel Good Together’ was launched. The campaign was intended to build upon the Healthy Ireland communications campaign launched in January and similarly targeted positive change in healthy eating, physical activity and mental wellbeing (Department of Health, 2018).    **Resilience and Recovery 2020-2021 Plan for Living with COVID-19**  The Resilience and Recovery plan set out in seven sections the Government of Ireland’s strategy for dealing with the Covid-19 emergency. In the sixth section (‘Growing our Resilience’) the plan states that Healthy Ireland and Sport Ireland will deliver a national campaign to promote the importance of being active (Department of an Taoiseach and Department of Health, 2020),    **#InthisTogether Campaign**  In April 2020, the Government announced the #InthisTogether campaign (Department of An Taoiseach, 2020b). The campaign was launched to encourage the Irish public to pick new activities which could help them to feel a little healthier during a period of restriction caused by the Covid-19 emergency.    **Keep Well Campaign**  In October 2020, the new coalition government announced the Keep Well campaign. The intention of ‘Keep Well’ was to build upon the #InThisTogether campaign. The ‘Keeping Healthy’ campaign was based on five themes: ‘Keeping active’, ‘Staying connected’, ‘Switching off and being creative’, ‘Eating Well’ and ‘Minding your Mood’ (Department of Health 2020a).    **Let's Get Set / Off the Couch Campaign**  The Off the Couch campaign was announced in 2021. The intended launch date of this awareness campaign was 2019 but it was postponed due to the Covid-19 emergency.  The ‘Let's Get Set’ / ‘Off the Couch’ campaign was organised around three themes: ‘Keeping active’, ‘Eating Well’ and ‘Minding your Mood’.    **Let’s Get Back Campaign**  As part of Let’s Get Set, the ‘Let’s Get Back’ campaign was launched in October 2021 with the intention of encouraging the Irish public to return to sport and physical activity following the removal of restrictions put in place during the Covid-19 pandemic (Sport Ireland, 2021b). The Let’s Get Back was a collaboration between Healthy Ireland, Sport Ireland, the National Governing Bodies (NGBs) for Sport, and Local Sport Partnerships (LSPs). The campaign was fronted by Olympic gold winning boxer Kellie Harrington, former rugby player Tommy Bowe, activist Joanne O‘Riordan and GAA manager  Jamie Wall.    **Get Ireland Active! National Physical Activity Plan for Ireland**  Action 2 of NPAP states that the Department of Health will conduct an annual campaign to promote physical activity (Healthy Ireland, 2016 p35). The latest implementation report cite the Healthy Ireland Communications Campaign (mentioned above), Healthy Ireland presence at the Bloom Festival and National Ploughing Championship, collaboration with the National Transport Authority and Healthy Ireland support for the Federation of Irish Sports' 20x20 campaign as evidence of implementation activities (Healthy Ireland et al., 2021).  **National Sports Policy 2018 – 2027**  Action 4 of the Nationals Sports Policy promises an educational campaign for parents and guardians to help them improve children’s physical literacy and attitudes towards sport and physical activity. The campaign is to be delivered in collaboration with the Department of Children and Youth Affairs, LSPs, NGBs, and the HSE (Department of Transport, Tourism and Sport, 2018 p29).    **Women in Sport Policy**  An action of Sport Ireland’s Women in Sport policy commits to “Develop and implement a Women in Sport Communications Plan” (Sport Ireland, 2018 p15).    **Safefood START campaign**  START is a public health campaign run by Safefood, in collaboration with public health agencies in Northern Ireland and the Republic. The message of the START campaign is that there are seven things that parents can do for their children to help them maintain a healthy weight   1. Eat fewer treat foods 2. Drink water and milk as regular drinks 3. Give the right portion size for children 4. Eat more fruit or vegetables. 5. Be more physically active 6. Limit screen-time 7. Increase sleep-time.   The START campaign runs ads on a variety of media including TV, radio, outdoor posters and social media. The START website contains information on the physical activity guidelines for children as well as advise on how families can be more active together (Safefood, 2022).  **Participation Plan 2021-2024 Increasing Participation in a Changing Ireland**  Action 11 of Sport Ireland’s Participation plan states that Sport Ireland will develop a communications plan to promotes sport participation. Action 12 states that Sport Ireland will work with sport governing bodies and local sport partnerships to ‘active’ national campaigns at a local level (Sport Ireland, 2021a).  **National Sports Policy 2018 – 2027 Sports Action Plan 2021 – 2023**  Action 2.1 of the Sport Action Plan states that a multi-annual, nationwide communications campaign will be undertaken to support the delivery of the National Sports Policy. The campaign it is stated will target women minorities, people with disabilities and communities with lower levels of participation (Department of Tourism, Culture, Arts, Gaeltacht, Sport and Media, 2021). |

| **MM02**  **There are clear, consistent policies to ensure that multiple media modes/channels (e.g., via posters, social media, radio as well as TV) combined with complementary community initiatives are used to promote the benefits of physical activity and disseminate guidelines which align with the WHO physical activity recommendations.** | |
| --- | --- |
| **Definitions and scope** | - Includes policies that are aligned with the WHO physical activity recommendations and are targeted at promoting the benefits of physical activity. - Includes frameworks, strategies or implementation plans targeted at promoting community PA initiatives that are partly or fully funded, managed or overseen by the government. |
| **International best practice examples (benchmarks)** | **Canada**  ParticipACTION is an initiative combining branding, sports events and mass media initiatives dedicated to promoting healthy living and physical fitness. It originated as a government programme in the 1970s. ParticipACTION has run numerous campaigns and public service advertisements (PSAs) on television, on radio, in newspapers, magazines and in corporate publications. Examples of activities that increased public awareness include the 1973 PSA “The 60 year old Swede & 30 year old Canadian” which highlighted the issue of physical fitness via international comparison (ParticipACTION, 2022). Another activity that ParticipACTION sponsored was Sports Day in Canada (SDIC), which sought to increase participation in sporting activity. This annual weeklong celebration of sport was run in collaboration with the Royal Bank of Canada (RBC) and the broadcasters Sports Radio Canada (SRC) and Canadian Broadcasting Corporation (CBC). A study of the effects of SDIC concluded that it significantly increased intentions to participate in sport (White et al., 2016).  **Netherlands**  Stoptober: dedicated month with activities/social support/norm to help people stop smoking. Supported by health funds and municipal health services.  ”30 Minuten Bewegen” (30 minutes of PA) included an ’energy tour’ which organised activities in schools (lower educational level) to promote PA, e.g. clinics by sport role models, PA week with activities. |
| **Context e.g. EU action / regulation** | **WHO**  **Global Action Plan on Physical Activity**  Proposed sub action for member states 1.1.2: Implement sustained public education, awareness and behaviour-change campaigns using traditional, social and digital mass reach communication channels, combined with complementing community initiatives, to increase the understanding of and positive attitudes towards physical activity, and promote the different ways everyone can increase physical activity and reduce sedentary behaviour (WHO, 2018). |
| **Evidence of implementation** | **Ireland**  **Healthy Ireland, A Framework for Improved Health and Wellbeing 2013- 2025**  The Healthy Ireland campaign, mentioned above, ran advertisements promoting PA on national TV and radio, as well as online video players, cinema, and social and digital media. There was also outdoor advertising in areas of high footfall.    **Get Ireland Active! National Physical Activity Plan for Ireland**  Action 1 of NPAP states that all stakeholders, led by the Department of Health, will collaborate to develop a three-year communications strategy. The strategy is intended to include the development of ‘print, online and social media resources’. Furthermore, Action 3 of NPAP states that a Get Ireland Active website will be developed as a ’one-stop shop’ for PA information. The deadline for both of these actions is listed as 2016. |

DOMAIN 6 – Community

**There are policies and programmes that promote and support physical activity for all ages and abilities, consistent with relevant recommendations, e.g. by supporting the implementation of whole-of-community events and approaches, and promoting the shared use of public spaces and facilities.**

| **C01**  **Public policies are in place to support the implementation of whole-of-community approaches to promote physical activity and networking to strengthen resources and exchange experiences (e.g., WHO Healthy Cities, Active Cities, Partnerships for Healthy Cities).** | |
| --- | --- |
| **Definitions and scope** | - Includes frameworks, strategies or implementation plans aimed at providing PA opportunities in local public spaces. - Includes approaches that collaborate with voluntary and for-profit organizations and non-government organisations (NGOs) |
| **International best practice examples (benchmarks)** | **Dominican Republic, Santo Domingo**  The city of Santo Domingo has collaborated with the initiative Partnership for healthy cities (a joint initiative of WHO and Bloomberg philanthropies) to implement a bicycle action plan (Lei Ravelo 2018; Valdivia 2018; Bloomberg Philanthropies Support LLC 2021).  **Lithuania – Public health bureaus**  Public health bureaus were established in 2006 in Lithuania to strengthen public health (including PA) at the municipal level. Municipal governments must fund public health bureaus in order to receive additional resources though bureaus also receive funding from European funds. Municipalities where public health bureaus were established first were assessed as having greater levels of public health knowledge and greater levels of participation in health promoting activities (WHOROE and EC 2018). |
| **Context e.g. EU action / regulation** | **WHO**  **Global Action Plan on Physical Activity**  Action 3.6: Implement whole-of-community initiatives, at the city, town or community levels, that stimulate engagement by all stakeholders and optimize a combination of policy approaches, across different settings, to promote increased participation in physical activity and reduced sedentary behaviour by people of all ages and diverse abilities, focusing on grassroots community engagement, co- development and ownership.  Proposed sub action for member states 3.6.1: Strengthen or establish national and/or subnational (municipality or local authority) networks of cities and communities implementing whole-of-community approaches to promote physical activity and share guidelines, resources and experiences (e.g., WHO Healthy Cities, Active Cities, Partnerships for Healthy Cities) (WHO, 2018). |
| **Evidence of implementation** | **Ireland**  **Healthy Cities and Counties of Ireland Network**  The Healthy Cities and Counties Programme is an initiative of the WHO, initiated in 1986. In November 2016, the National Healthy Cities and Counties of Ireland Network was launched. The stated aim of the Network is to support local authorities to implement the Healthy Ireland Framework (Department of Health, 2019c). Presently the network has a membership of 17 participating local authorities, the county councils of Cavan, Carlow, Clare, Laois, Leitrim, Longford, Louth, Mayo, Offaly, Roscommon, South Dublin, Tipperary and Waterford, the city councils of Cork, and Galway and the city and county councils of Limerick and Waterford.    **National Sport Policy 2018 – 2027**  Action 8 of the National Sport Policy is to establish, through Sport Ireland, an initiative to support Local Authorities in developing Local Sports Plans consistent with the overall vision and objectives of this National Sports Policy. The Local Plans will review needs and set out actions to increase participation locally. It will be developed and implemented in cooperation with LSPs, clubs, communities and partners within and beyond sport (Department of Transport, Tourism and Sport, 2018).  **Sport Ireland’s Active Cities**  Sport Ireland has allocated funding for an 'Active Cities' project in line with principles of the WHO’s Global Action Plan on Physical Activity. A 2020 document reports that €825 000 will be allocated to seven urban local sports partnerships (LSPs) through this fund. The fund is designed to invest in initiatives which create social norms around the benefits of sport and physical activity (Sport Ireland, 2020 p4; WHO, 2018).    **Bike week**  The Department of Transport funds local authorities and Local Sports Partnerships to run “Bike Week” an annual effort to promote cycling. In 2022, Bike Week will run from the 14th to the 22nd of May (National Transport Authority, 2022).  **Community Sports and Physical Activity Hubs.**  Community Sports and Physical Activity Hubs (CSPAHs, see Education 03) has been described as “a collection of progressive sports clubs and other local organisations that want to work together to improve sport offered in their local community”. Through the Hubs provide information, support and advice on a wide range of sports and physical activities to make it easier for people in disadvantaged areas to engage in a more active lifestyle. In 2019, Sport Ireland published a report evaluating the outputs of the first nine CSPAHs to be established (Sport Ireland, 2019).  **European Week of Sport**  The European Week of Sport is an initiative of the European Commission, co-ordinated in Ireland by Sport Ireland. In 2021, European Week of Sport ran from the 23rd to the 30th of September (O’Mahoney, 2021). |

| **C02**  **There are public policies in place to foster partnerships for shared use of public spaces and facilities for community-based and community-led physical activity programmes.** | |
| --- | --- |
| **Definitions and scope** | - Includes partnerships with voluntary and for-profit organizations and non-government organisations (NGOs) |
| **International best practice examples (benchmarks)** | No suitable examples identified. |
| **Context e.g. EU action / regulation** | **WHO**  **Global Action Plan on Physical Activity**  GAPPA Action 3.3: Enhance provision of, and opportunities for, more physical activity programmes and promotion in parks and other natural environments (such as beaches, rivers and foreshores) as well as in private and public workplaces, community centres, recreation and sports facilities, and faith- based centres, to support participation in physical activity, by all people of diverse abilities.  Proposed sub action for member states 3.3.3: Partner and support the development of national sports policies that prioritize investment in active recreation and sports programmes which target the least active, disadvantaged, marginalized, stigmatized, and indigenous communities and populations, including people with mental and/or physical disabilities (WHO, 2018). |
| **Evidence of implementation** | **Ireland**  *In Ireland, provision of recreational amenities is primarily the responsibility of local government (Citizens Information, 2021a).*  **Get Ireland Active! National Physical Activity Plan for Ireland**  Action 41 of Get Ireland Active, The National Physical Activity Plan (Healthy Ireland, 2016) assigns responsibility for supporting Community Sport and Physical Activity Hubs (CSPAHs) to Sport Ireland (Healthy Ireland, 2016).  The latest NPAP implementation report states that Sport Ireland has invested €4,922,894 into 37 existing CSPAHs over the last 5 years. It also states that investment in a further 12 CSPAHs is planned (Healthy Ireland et al., 2021 p.37).  **National Sport Policy 2018 – 2027**  In this document, three actions specifically refer to foster partnerships, especially in regard to addressing the social and disability gradient in participation in sport. Action 9 seeks to promote collaboration between sporting bodies (NGBs, LSPs and clubs) and between the sport sector and other policy sectors on the development and promotion of participation initiatives. Action 11 seeks to support the private sector in rolling out initiatives such as Community Sports Hubs. Action 12 seeks to explore the possibility of introducing a national network of Sport Inclusion Disability Officers (SIDOs), aligned to the LSP network, which are expected to work closely with relevant NGBs, the disability sector, leisure center providers, the CARA Centre and other stakeholders (Department of Transport, Tourism and Sport, 2018).    **Action Plan for Rural Development**  Action 176 of the Action Plan for rural development proposes that the relevant department cooperate with the state claims agency to introduce a scheme which indemnifies private landowners with regard to recreational users of their land (Department of Children, Equality, Disability, Integration and Youth, 2017a).  The first progress report of the scheme reports that the state claims agency has issued a report supporting the introduction of such a scheme and that a submission proposing a course of action is being prepared for the Attorney General’s office (Department of Children, Equality, Disability, Integration and Youth, 2017b).    **Outdoor Recreation Plan (ORP)**  The Outdoor Recreation Plan (ORP) recognises the need for well-maintained outdoor facilities and seeks to increase provisions of outdoor facilities and opportunities (Coillte, 2017).    **Strategy for the Future Development of National and Regional Greenways**  Following a public consultation, a strategy for developing greenways was published in 2018. The preferred method of delivery for these amenities is to use state owned land. Where a proposed greenway route passes through private land it is proposed that voluntary agreements are entered into to sell the required land for the construction of the greenway. The majority of existing trails are developed on a ‘permissive access’ basis whereby the public may use the route but users must not cause damage to the amenity (Department of Transport, Tourism and Sport, 2018a).  **Great Outdoors. A guide for accessibility**  The Great Outdoors is a document prepared by Sport Ireland and the Dormant Accounts Fund in partnership with the Irish Wheelchair Association (IWA) Sport. The IWA is a representative body for people with disabilities and IWA Sport seeks to enable people with physical disabilities to participation in sports and recreational activity (IWA, n.d.). The Great Outdoors provides guidance, intended for a variety of organisations for designing outdoor spaces to ensure that they are accessible for people with disabilities. The Great Outdoors recommends a ‘Universal Design’ approach as necessary for providing access to people with disabilities. The Great Outdoors contains seven sections including an introductory section. Section two provides guidance on “Training, Consultation & Collaboration”, Section three provides guidance on “Information and Communication”, Section 4 deals with “Trails, Greenways & Public Parks”, Section 5 deals with “Beaches”, Section 6 deals with “Waterways” and Section 7 deals with “The Built Environment” (IWA et al., 2018).    **Community Facilities Scheme**  The Community Facilities Scheme was established in 2017 to provide a total of €2 million in funding to grass roots community projects. The scheme is administered by Local Community Development Committees (LCDCs) working alongside local authorities. The following types of projects can apply for funding of up to €1000:   - Development/renovation of community centres. - Community amenities. - Youth clubs. - Sports/recreation facilities. - Improvements to town parks and common areas and spaces. - Creative Ireland Programme 2017-2022 projects (Dun Laoghaire-Rathdown LCDC, 2017).   **Ready, Steady, Play! A National Play Policy**  The National Play Policy was developed to ensure that all Irish children have opportunities to participate in play. Emphasis is placed on ensuring that children have access to playgrounds and recreational facilities. An aim of the policy is to ensure that all playgrounds provided by public authorities conform to standards specified in the Irish Standards for play equipment and surfacing. Another aim of the policy is to bring the supply of playgrounds up to 1 per 10 000 population. It is estimated in the policy that the shortfall in provision at the time of policy’s preparation would require building 204 local playgrounds at a minimum cost of €16 million (National Children’s Office, 2004 p.57).    **Better Outcomes, Brighter Futures, the national policy framework for children & young people 2014 - 2020**  Commitment number 1.15 of the national policy framework for children and young people commits government to creating more recreational spaces for children and young people. Examples of such spaces that are provided include youth cafés, playgrounds and sports and leisure centres (Department of Children, Equality, Disability, Integration and Youth, 2019). |

DOMAIN 7 – Sport

**There are evidence-informed public policies implemented to promote and support sport and recreation for all.**

| **SP01**  **There are national and/or subnational evidence informed 'Sport and Recreation for All' policies that prioritise investment in initiatives that target the least active, as well as disadvantaged groups.** | |
| --- | --- |
| **Definitions and scope** | - Includes frameworks, strategies or implementation plans that specify aims, objectives or targets to reduce inequalities including taking a preventive approach that addresses the social and environmental determinants of health. - Implementation plans specify policies or programmes that aim to reduce inequalities for specific population groups. |
| **International best practice examples (benchmarks)** | **Lithuania**  An order of the Minister of the Environment, “On technical regulation for constructions 2.03.01:2001”, includes a requirement that sports-related constructions be easily accessible for people with disabilities. (WHO factsheets, 2015). |
| **Context e.g. EU action / regulation** | **WHO**  **Global Action Plan for Physical Activity**  Action 3.5.3 of GAPPA requests member states develop national sports policies that prioritise the least active and marginalised.  **EU**  **2013 Council Recommendations**  Indicators 6 was developed to gather information about the presence of a national sport for all policy or action plan (Council of the European Union, 2013). |
| **Evidence of implementation** | **Ireland**  **National Sport Policy 2018 – 2027**  The National Sport Policy (NSP) was developed through collaboration with the sport sector following a public consultation (Government of Ireland, 2018). The NSP has several dedicated actions listed to address disparities in participation caused by disadvantage.  Action 8 of the NSP states that Sport Ireland will establish an initiative to support local authorities to develop local sports plans to support sport participation locally and Action 9 of the NSP states that, in the development of local sports plans programmes, a focus on groups with lower levels of sport participation will be prioritised for funding. This includes people with disabilities (PWD) or people of low socioeconomic status (Department of Transport, Tourism and Sport, 2018, p36).  Action 12 of the NSP introduces a network of Sports Inclusion Disability Officers (SIDOs) to work with the agencies tasked with increasing participation at a local level (the Local Sports Partnerships – LSPs). The SIDOs have the responsibility of providing opportunities for PWDs to participate (Department of Transport, Tourism and Sport, 2018, p37).  Action 48 stipulates that Sport Ireland (SI – the agency established by statute to develop sport in Ireland) will fund pilot programmes aimed at addressing increasing participation in sport for people who are disadvantaged and PWDs (Department of Transport, Tourism and Sport, 2018, p105).    **Participation Plan 2021-2024 Increasing Participation in a Changing Ireland**  Action 4 of Sport Ireland’s participation plan (the single action in the theme ’Partners’) states that Sport Ireland will develop new and existing partnerships ‘to increase participation in sport and reduce participation gradients’ (Sport Ireland, 2021a p15).  Action 30 (in the theme ‘Youth’) supports the delivery of after school physical activity programmes focussing on socio economic disadvantaged areas.  Action 40 (theme: ‘Disability’) states that Sport Ireland and its partners will continue to build on work designed to reduce the barriers to participation of people with disabilities.  Action 41 (theme: ‘inclusion’) states that Sport Ireland and its partners will develop an inclusion policy base up research.  Action 42 (theme: ‘Ethnic minorities’) Sport Ireland and its partners will support participation by people from diverse ethnic backgrounds.  Action 43 (theme: ‘LGBTQ+’) states that Sport Ireland will promote inclusion of people from the LGBTQ+ community.  Action 44 (theme: ‘Socio Economic Disadvantage’) states that Sport Ireland and its partners will invest in programmes that enable sport participation in areas of socio economic disadvantage) and action 45 states that Sport Ireland will support participation in disadvantaged rural areas where transport to facilities poses a barrier (Sport Ireland, 2021).  **Sport Inclusion Disability Charter**  The charter was launched in 2018. It was intended that the charter would contribute to achieving the actions set out in NSP (Cork Sport Partnership, 2018).    **Sport Ireland Policy on Participation in Sport by People with Disabilities**  Sport Ireland’s Policy on promoting PA for PWD was published in 2020. The policy is designed to reduce the gradient in participation between people with disabilities and people without disabilities. The policy has 14 actions. These include: designating an organisation, CARA, to fulfil the commitments in NSP regarding participation amongst PWD, commit grantees to sign up to the disability charter, include disability participation targets in all future strategies and accounts of disability participation activities in all future annual reports (Sport Ireland, 2018a).    **Women in Sport Policy**  Sport Ireland’s Women in Sport Policy is designed to link with NSP. Its ultimate objective is to eliminate the gender difference in sport participation rates (Sport Ireland, 2018b).  **Diversity and Inclusion plan**  A diversity and inclusion plan is currently under development at Sport Ireland (Sport Ireland, 2021c).    **Sport Capital and Equipment Programme**  The Sport Capital and Equipment Programme (SCEP) requires that all equipment and facilities funded are accessible to people with disabilities. This is a requirement of the Sport Ireland Policy on Participation in Sport by People with Disabilities (Department of Tourism, Culture, Arts, Gaeltacht, Sport and Media, 2019).    **The Migrant Integration Strategy A Blueprint for the Future 2017 – 2020**  Action 4 of the National Migrant Integration Strategy states that funding decisions for community and sport organisations with include criteria based on whether the organisations have activities which promote migrant integration (Department of Justice and Equality, 2017).  Action 73 pledges to encourage the integration of migrants into Irish society via sport. Sport Ireland is listed as a partner in this endeavour (Department of Justice and Equality, 2017).    In 2019, the department published a report evaluating progress on the various actions of the strategy. Each action was received one of the four possible ratings: “On track”, “Minor Problems or Delays”, “One or More Major Difficulties” or “complete”. Action 4 received a “Minor Problems or Delays” rating and action 73 received an “On track” rating (Office for the Promotion of Migrant Integration, 2019). |

| **SP02**  **There are national and/or subnational evidence informed policies or action plans in place that ensure equitable access to sport and recreation spaces and places for all.** | |
| --- | --- |
| **Definitions and scope** | - Evidence informed means the best available research evidence is used to inform decision-making. It is characterized by systematic and transparent access to and appraisal of evidence as an input into the policy-making process (WHOROE, 2021). - Equitable entails the absence of avoidable, unfair, or remediable differences among groups of people, whether those groups are defined socially, economically, demographically or geographically or by other means of stratification (PEN Glossary, In Press). |
| **International best practice examples (benchmarks)** | **Finland**  Section 5 of the Act on the Promotion of Sports and Physical Activity (2015) assigns responsibility for creating opportunities and facilities to local government. The provision of services is required to involve consultation with local residents with due regard to the various target groups. Finland has a high level of sports infrastructure provision, 71% of which is owned by local government (Bergsgard *et al.,* 2019). |
| **Context e.g. EU action / regulation** | **Council of Europe**  Members of the Council of Europe committed to implement the Sport for All Charter (Council of Europe, 1976). Article I stipulate that every individual has the right to participate in sport. Article VI recognises that participation in sport is dependent on access to facilities and stipulates that planning of sports facilities is a matter for public authorities.  **EU**  **Nice Declaration of 2000**  The European Council met in Nice in 2000 in the context of the negotiation of the Treaty of Nice. The council, which provides the European Union with its agreed policy direction, issued its conclusions the annexes of which make clear that the primary responsibility for the conduct of sporting affairs lies with member states and sporting organisations. The European Council recommends that sporting activity should be accessible to every man and woman with due regard for aspiration and ability (European Council, 2000). The European Commission White Paper on Sport reiterates that primary responsibility for sporting affairs is with member states (European Commission, 2007).  **2013 Council Recommendations**  Indicator 8 of the proposed framework seeks information on whether there is a framework to increase access to sport and recreation facilities for persons of low socio-economic status (Council of the European Union, 2013). |
| **Evidence of implementation** | **Ireland**  **Participation Plan 2021-2024 Increasing Participation in a Changing Ireland**  Action 35 (theme: ‘Exercise facilities’) states that Sport Ireland and its partners will seek to incentive exercise facilities and sport service providers to tackle gradients in sport participation.  Action 44 (mentioned in Sport 01) states that Sport Ireland and its partners will invest in programmes that enable sport participation in areas of socio economic disadvantage) and action 45 states that Sport Ireland will support participation in disadvantaged rural areas where transport to facilities poses a barrier (Sport Ireland, 2021a).  Action 47 (‘outdoors’) 8 states that local authorities will be supported to maximise the use of recreational areas via the Local Sport Plans.  **Sports Capital and Equipment Programme**  The Sports Capital and Equipment Programme (SCEP) funds facilities targeted at increased participation. A scoring system, which is periodically reviewed, is in place to ensure that the programme is contributing to its stated objectives.    Allocation decisions are made by the department with responsibility for sport. Presently applications for funding are based on six criteria:   1. Likelihood of increasing participation or improving performance 2. Sharing of facilities 3. Level of socio-economic disadvantage in the area 4. Technical merits of the project 5. Level of self-funding 6. Level of Sports Capital and Equipment Programme   funding received in the past 10 years (Department of Tourism, Culture, Arts, Gaeltacht, Sport and Media, 2019a).     **Large Scale Sport Infrastructure Fund**  The Large Scale Sport Infrastructure Fund (LSSIF) supports sports facility projects that require a larger amount than is available under the SCP (Department of Tourism, Culture, Arts, Gaeltacht, Sport and Media, 2019b). This includes projects that increase participation in some cases (for example, swimming pools are considered under LSSIF). The LSSIF received an allocation of €100 million from the government for the period 2019 to 2027. |

| **SP03**  **There is government support for programs designed to encourage sports clubs to promote health-enhancing physical activity and other health behaviours (e.g., 'sports clubs for health' and 'health promoting sport clubs').** | |
| --- | --- |
| **Definitions and scope** | - Sports clubs refers to organisations whose primary activity is to enable members to participate in sport, whether for competitive or recreational purposes. - Sports clubs for health is an approach to sports club management which aims to increase the availability and quality of health-enhancing sports activities. - Health-enhancing sports activities are defined as any ‘sports activities that are beneficial to health and that present no or only minimal health and safety risks.’ (Sports club for Health, 2020). - Health promoting sports club is an approach whereby sports clubs support health promotion in their activities. - Support includes guidelines, toolkits, templates (e.g. policy/guidelines or contracts), PA sessions planning tools/equipment, expert advice, online training modules, and training workshops or courses. |
| **International best practice examples (benchmarks)** | **Australia**  Victorian Health Promotion Foundation, or VicHealth, is an independent health promotion agency supported by the government of the Australian state of Victoria. VicHealth was established under the 1987 Tobacco Act and is funded from the state budget since 1997 (VicHealth, 2005). VicHealth provides grants to sports clubs who implement practices that support public health. VicHealth’s Active Club Grants initiative provides grants to sports clubs that support increased physical activity. The grants were especially targeted for disadvantaged communities including indigenous communities, older people, younger people and people with disabilities (VicHealth, 2012). Recently the grant has been focused on people female participation and social and modified sport 3.  **Scotland**  The Community Sports Hubs initiative was developed to increase participation in sport in Scotland. The support provided is intended to for people of all ages. |
| **Context e.g. EU action / regulation** | **EU**  **European Council**  Indicator 7 of the HEPA monitoring framework requests implementation of the guidelines for health-oriented sports clubs developed by the Sports Clubs for Health Programme.  The European Council recommends that sporting activity should be accessible to every man and woman with due regard for aspiration and ability. The European Council supports the independence of sports organisations and their right to organise themselves.  **Sports Clubs for Health (project)**  The third international Sports Club for Health (SCforH) project promotes the adoption of the SCforH approach. The SCforH project hosts an online training course on the principles of the SCforH approach on its website as well as information on how to apply them in a sports organisation. The SCforH approach is also laid out in a textbook available at the same location (Sports club for Health, 2020). |
| **Evidence of implementation** | **Ireland**  **National Sport Policy 2018 – 2027**  Chapter three of NSP contains actions that will be taken to promote participation. Most relevant for this statement is action 14 which states: “We will use our funding streams to foster social participation in sport by encouraging the development of strong community-based sports organisations (e.g. Community Sports Hubs), supporting and developing those who volunteer for sport and promoting social membership of sports clubs” (as well as playing membership) (DTTS, 2018; p38).  **GAA National Healthy Club Project (partnered with HSE)**  The Gaelic Athletic Association (GAA) is a sporting organisation which promotes traditional Irish sports such as Hurling and Gaelic Football. The GAA’s Healthy Clubs Project aims to assist GAA clubs to support the health of their members and communities (GAA, 2016a). Over 300 clubs are reported to be participating in the Healthy Club Project which is based on the Sports Club for Health Guidelines (WHOROE and European Commission 2021). The GAA has 2200 clubs across the Republic and Northern Ireland (GAA, 2016b).  Healthy Ireland, working with the GAA is seeking to recruit 150 – 200 further clubs into the programme in 2022 and to investigate the potential of expanding the programme with other sporting organisations (Healthy Ireland, 2021).    **Community Sports and Physical Activity Hubs**  The Community Sports and Physical Activity Hubs (CSPAHs) project is intended to increase the number of people of all ages participating in sport and physical activity. In 2019, Sport Ireland published a report evaluating the outputs of the first nine CSPAHs to be established (Sport Ireland, 2019). |

DOMAIN 8 – Workplace

**There are national and/or sub-national policies implemented related to the workplace that promote and support increasing physical activity (e.g. cycle to work initiatives, physically active workplaces) and promote a culture of health for all employees.**

| **W01**  **There are national and/or sub-national policy initiatives and infrastructure development programmes in place to promote and support safe active travel to and from the workplace.** | |
| --- | --- |
| **Definitions and scope** | - Includes standards for the public sector which promote and support safe active commuting to and from the workplace. - Includes both public and private sector workplace settings. |
| **International best practice examples (benchmarks)** | **Netherlands**  The work-related expenses scheme (Wekkostenregeling) allows employers to provide a tax-free travel allowance provided the allowance is less than 1.5% of salary costs (KING Software, 2021).  **United States, Massachusetts:** Massachusetts wellness tax credit. Businesses with 200 employees or fewer may apply for a tax credit for wellness programmes. The tax credit is valued at up to 25% of the cost of implementing the programme (Massachusetts Department of Public Health, 2021) |
| **Context e.g. EU action / regulation** | **WHO**  **Physical activity strategy for the WHO European Region 2016–2025**  Objective 3.2: “Provide opportunities and counselling for physical activity at the workplace. Member States should consider adopting appropriate measures to promote active commuting and the use of public transport to travel to work. The measures could include regulations, guidelines or financial incentives for companies with regard to cycle racks, changing rooms, showers and adequate public transport options” […](WHOROE, 2016).  **EU**  **2013 Council recommendations**  Indicator 19: Schemes to promote active travel to work. Existence of a national or sub-national (where relevant) incentive scheme for companies to promote physical activity at the workplace (e.g. gyms, showers, walking stairs etc.) (Council of the European Union, 2013). |
| **Evidence of implementation** | **Ireland**  **Cycle to work scheme**  Under the cycle to work scheme, an employer can purchase a new bicycle and cycling accessories (up to a value of €1250) for their employee (Citizens Information, 2021b). The employee repays the employer for the cost of the equipment in instalments deducted from their salary. Tax, Universal Social Care (USC) or Pay Related Social Insurance (PRSI) are applied to an employee’s salary after the deduction has been applied. This means that the employee is taxed on lower proportion of their total income (Revenue Commissioners, 2021).    **Smarter Travel Workplace**  Action 8 of the Smarter Travel Policy (STP) commits the Government of Ireland to require all organisations with over 100 staff to implement workplace travel plans. This requirement led to the Smarter Travel Workplace programme. According to a report compiled by the department with responsibility for transport, Smarter Travel Workplaces engaged with 97 larger employers with 105 000 employees in 2018 (Department of Transport, Tourism and Sport and Department of Environment, Community and Local Government, 2019c). |

| **W02**  **There are concepts and regulations for buildings, plots and the environment in place that promote and support employers to create physically active workplace environments through building design and provision of adequate facilities (both indoor and outdoor).** | |
| --- | --- |
| **Definitions and scope** | - Includes policies and regulations which promote and support employers (both public and private) to create a physically active environment. - Facilities includes building features that support physical activity (e.g. showers, bicycle rakes). |
| **International best practice examples (benchmarks)** | **USA, New York**  Though not a regulation, the active living guidelines were developed with city agencies (Bloomberg *et al.,* 2010) |
| **Context e.g. EU action / regulation** | **WHO**  **Global Action Plan on Physical Activity**  Action 2.5: Strengthen the policy, regulatory and design guidelines and frameworks at the national and subnational levels, as appropriate, to promote public amenities, schools, health-care, sports and recreation facilities, workplaces and social housing, that are designed to enable occupants and visitors with diverse abilities to be physically active in and around the buildings, and prioritize universal access by pedestrians, cyclists and public transport.  Proposed sub action for member states 2.5.6: Industry, Guilds, Labour, Unions, Occupational Health and Safety and other related organizations should develop and implement guidance to support employers create healthy workplaces that support physical activity and reduced extended periods of sedentary behaviour during the working day, and encourage active lifestyles of their employees and families (WHO, 2018).  **WHOROE**  **Physical activity strategy for the WHO European Region 2016–2025**  Objective 3.2: “Provide opportunities and counselling for physical activity at the workplace […] Member States may consider adopting appropriate measures, such as regulations and guidelines concerning health at the workplace, to enable more physical activity during the working day. The measures could include action to address the workplace layout, such as the provision of adjustable desks, prominent and promotional signs on staircases encouraging their use […].” (WHOROE, 2016). |
| **Evidence of implementation** | **Ireland**  **Finance Act 2018**  Section 19 of the Finance Act of 2018 creates a capital allowance for buildings used by employers for the purposes of providing childcare fitness facilities for employees. A fitness facility is described in the act as “a gymnasium used exclusively in providing a range of facilities designed to improve and maintain the physical fitness and health of participants”. |

Physical Activity Environment Policy Index: Infrastructure Support Domains

DOMAIN 9 – LEADERSHIP

**The political leadership ensures that there is strong support for the vision, planning, communication, implementation and evaluation of policies to create health-promoting policy environments to improve population physical activity and reduce related inequalities.**

| **L01**  **There is strong, visible, political support (at the head of state/cabinet level) for creating health-promoting policy environments to improve population levels of physical activity and reduce inactivity related non-communicable diseases and their related inequalities. Political responsibility for health-related physical activity is clearly allocated within the governmental structures.** | |
| --- | --- |
| **Definitions and scope** | - Visible support includes statements of intent, election commitments, budget commitments, establishing priorities and targets, demonstration of support in the media, other actions that demonstrate support for new or strengthened policy. - Documents that contain evidence of strong political support include media releases, speeches, pre-election policy papers, introduction of a bill, State-level strategic plans with targets or key performance indicators (Food EPI Evidence Document - LEAD1). |
| **International best practice examples (benchmarks)** | **United States**  The First Lady of the United States (FLOTUS), Michelle Obama, initiated the Let’s Move Campaign. The office of the first lady is a part of the executive office of the presidency (Let’s Move! 2021) |
| **Context e.g. EU action / regulation** | **WHO**  **Global Action Plan on Physical Activity**  Action 4.1: Strengthen policy frameworks, leadership and governance systems, at the national and subnational levels, to support implementation of actions aimed at increasing physical activity and reducing sedentary behaviours, including multisectoral engagement and coordination mechanisms; policy coherence across sectors; guidelines, recommendations and actions plans on physical activity and sedentary behaviour for all ages; and progress monitoring and evaluation to strengthen accountability.  Proposed sub action for member states 4.1.1: Initiate or strengthen, as appropriate, a high-level national multisectoral coordination committee to provide leadership, strategic planning and oversight of implementation and monitoring of national policy actions on physical activity and sedentary behaviour, ensuring appropriate representation from all relevant areas and levels of government, as well as nongovernmental stakeholders and the community (WHO, 2018). |
| **Evidence of implementation** | **Ireland**  **Programme for Government: Our Shared Future**  The 2020 Programme for Government states that the Government will “Promote more physical exercise among all sections of the community” (Department of An Taoiseach, 2020a).  **Healthy Ireland, A Framework for Improved Health and Wellbeing 2013- 2025**  The 'Healthy Ireland' Framework was launched in 2013 with a foreword by the then Taoiseach Enda Kenny and an introduction by the then Minister for Health Dr. James Reilly, TD. It articulates four central goals for improved health and wellbeing:   - Increase the proportion of people who are healthy at all stages of life. - Reduce health inequalities. - Protect the public from threats to health and well being. - Create an environment where every individual and sector of society can play their part in achieving a healthy Ireland.     The Cabinet Committee on Social Policy oversees the delivery of this framework. The Health and Wellbeing Programme, in the Department of Health, is responsible for strategic planning and implementation of the framework actions. A multi-stakeholder body, the Healthy Ireland Council, was established to provide a national advisory forum supporting the implementation of the framework across sectors. The framework was accompanied later in 2013 by a high-level implementation plan and an outcomes framework (Department of Health, 2013b).   The 'Healthy Ireland' framework was later updated by the 'Healthy Ireland Strategic Action Plan 2021–2025' (Healthy Ireland, 2021).  **Get Ireland Active! National Physical Activity Plan for Ireland**  Action 56 of the National Physical Activity Plan (NPAP) establishes a NPAP implementation group. The NPAP Implementation Group is co-chaired by the department with responsibility for health and the department with responsibility for sport (Healthy Ireland, 2016) (see statements Leadership 02 and Governance 04).    **A Healthy Weight for Ireland, Obesity Policy and Action Plan 2016-2025**    The OPAP was launched with a Foreword by the then Minister for Health, Simon Harris TD, and a Foreword by the then Minister of State for Health Promotion, Marcella Corcoran Kennedy TD. The policy sets out "Ten Steps Forward" which aims at reversing the obesity trend while preventing complications associated with obesity and reducing the overall burden for individuals, their families and the health system. The "Ten Steps Forward" are:   1. Embed multi-sectoral actions on obesity prevention with the support of government departments and public sector agencies. 2. Regulate for a healthier environment. 3. Secure appropriate support from the commercial sector to play its part in obesity prevention. 4. Inform and empower change through a clear communications strategy. 5. The Department of Health will provide leadership. 6. Mobilise the health services with a focus on prevention. 7. Develop a service model for specialist care for children and adults. 8. Acknowledge the key role of physical activity in the prevention of overweight and obesity. 9. Allocate resources according to need in particular for children and disadvantaged groups. 10. Monitor research and review.    A leadership group, the Obesity Policy Implementation Oversight Group (OPIOG), was established in October 2017 to oversee implementation of OPAP. OPIOG is chaired by the Department of Health.  **National Sport Policy 2018 – 2027**  Action 39 of NSP states: “We will establish a Sports Leadership Group within three months of the publication of this policy to agree an Action Plan and to oversee its implementation once approved by Government” (Department of Transport, Tourism and Sport, 2018; p83). The Sports Leadership Group established includes representatives from the Departments of Education (DoE); Health (DoH); Children, Equality, Disability, Integration and Youth (DCEDIY) and Tourism, Culture, Arts, Gaeltacht, Sport and Media (DTCAGSM).  **Sláintecare Reforms**  The Sláintecare Reforms have been described as a “cross-party political consensus on the future of our health and social care services” (Department of Health, 2020b). The Sláintecare Report sets out a vision for an integrated care service, that shifts away from a hospital-centric model of care towards a system with great focus on health promotion and public health. The report recommends that the role of Minister of State for Health Promotion should be retained in future governments and that the Minister for Health is held responsible on a legislative basis for the delivery of health system reform (Houses of the Oireachtas, 2017). To achieve Sláintecare’s health promotion objectives several programmes and initiatives were launched including the Sláintecare Healthy Communities Programme and the Healthy Campus and Healthy Workplace Frameworks.  **Sláintecare Healthy Communities Programme**  The Sláintecare Healthy Communities Programme was introduced in the Sláintecare Implementation and Action Plan for the period 2021 to 2023 (Government of Ireland, 2020). The purpose is to improve health and wellbeing in the most disadvantaged areas of the country by addressing disparities in the social determinants of health (the non-medical factors that influence health outcomes). Initially, nineteen disadvantaged areas were identified. A variety of interventions targeting the social determinants of health will be delivered via the programme including health specific interventions by the Department of Health and the HSE and the wider supports provided by other government agencies and departments.  **Healthy Workplaces Framework**  The Sláintecare report recognises that health inequalities are influenced by the social determinants of health related to employment. The Healthy Workplaces Framework sets out the governments vision to support workplaces to promote health and wellbeing including through physical activity. The framework contains a list of actions detailing how the government will engage with employers to promote health in the workplace (Healthy Ireland 2021).  **Healthy Campus**  The Healthy Campus Charter and Framework was developed by the Department of Health and the HSE in collaboration with higher education institutions. By signing the charter, Higher Education Institutions commit to promoting health and wellbeing using a ‘whole campus approach’. The Framework document sets out a process by which Higher Education Institutions can promote health.    **Healthy Ireland Fund**  In 2016, the government approved the creation of a Healthy Ireland Fund (HIF) with an initial allocation of €5 million, approved in Budget 2017. The fund established to support the implementation of Healthy Ireland programmes and projects in a variety of settings. The HIF seeks to support innovative, cross-sectoral, evidence-based projects and initiatives that support the implementation of key national policies in areas such as obesity, smoking, alcohol, physical activity and sexual health. The funding has been allocated to 2 Strands, both of which are closed calls for funding. Strand 1 focuses on the delivery of local initiatives and Strand 2 funds national actions (Pobal, 2016).  The Department of Health has approved a third round of funding for the HIF. The aim of this funding is to support local and national organisations to deliver actions that will improve health and wellbeing in line with ‘Healthy Ireland, A Framework for Improved Health and Wellbeing 2013-2025’ (Department of Health, 2019).    Target Groups  All actions with a focus on supporting the health and wellbeing of the population in general are eligible for HIF support. However, the following are the priority target groups for this round of funding:   - Disadvantaged communities - Disadvantaged men and women - Disadvantaged families, including one parent families - Children and young people - People with disabilities, including people with mental health issues - Unemployed young people and adults - Traveller and Roma communities - New communities, asylum seekers and refugees - LGBTI - Homeless people     **Healthy Ireland campaign (2019)**  Taoiseach Leo Varadkar TD, Minister for Health Simon Harris TD and Minister for Health Promotion, Catherine Byrne TD have launched the 2019 Healthy Ireland campaign and announced funding of €1 million to boost community engagement on health and wellbeing in every county. The 2019 campaign seeks to raise public awareness of the Healthy Ireland website (gov.ie/HealthyIreland) as a resource where trusted sources of information and supports are available to people seeking to make healthy changes (Department of Health, 2019).    **National Healthy Cities and Counties of Ireland Network**  National Healthy Cities and Counties of Ireland Network was launched in 2016 by the Minister for Health Promotion. The network was launched under the ‘Healthy Ireland' framework, and has representatives from government departments, the HSE, the Institute of Public Health, the Federation of Irish Sport and local political and community representatives (Department of Health, 2019) The network aims to:   - Promote lifelong health and wellbeing - Provide a means where local issues can influence national policy   Provide a voice for Ireland in the WHO Network of European National Healthy Cities Networks (Department of Health, 2019). |

| **L02**  **There is a comprehensive up-to-date plan (including timeline, targets, funding, priority policy and programme strategies) linked to national needs and priorities to increase population physical activity.** | |
| --- | --- |
| **Definitions and scope** | - Includes documented plans with specific actions and interventions (i.e. policies, programs, partnerships) - Plans should be current (i.e. maintain endorsement by the current government and/or are being reported against) - Plans should refer to actions to improve physical activity environments (as defined in the policy domains above) and should include both policy and program strategies - Excludes overarching frameworks that provide general guidance and direction - Includes priority policy and program strategies, social media marketing for public awareness and threat of legislation for voluntary approaches (Food EPI Evidence Document – LEAD4) |
| **International best practice examples (benchmarks)** | **Luxembourg**  Four Ministries (Ministry of Family, Integration and the Greater Region, the Ministry of National Education, Children and Youth, the Ministry of Sports and the Ministry of Health) have come together to form an interministerial committee which coordinates the promotion of physical activity in the Duchy of Luxembourg. The committee has developed the national framework for promoting healthy diet and physical activity in Luxembourg (Gesond iessen, Méi bewegen – GIMB) (Le Gouvernement du Grand-Duché de Luxembourg, 2017). |
| **Context e.g. EU action / regulation** | **WHO**  **Global Action Plan on Physical Activity**  Proposed sub action for member states 4.1.2: Strengthen current, and where necessary develop new, national and subnational action plans on physical activity and sedentary behaviour which align with recommendations in global and regional guidance, and maximize policy coherence and synergies with relevant priorities across key sectors including, but not limited to, transport, urban planning, health, social care, education, tourism, and sports and recreation (WHO, 2018).  **EU Action**  **Council Recommendation of 26 November 2013 on promoting health-enhancing physical activity across sectors**  In 2013, the Council of the European Union issued four recommendations to member states. The first recommendation extorts member states to utilise a cross sectoral approach in developing HEPA policies (Council of the European Union, 2013).  **European Commission**  The Directorate General for Health and Food Safety (Sante) within the European Commission has as an objective to prom.ote cost effective health promotion and disease prevention. Indicator 1.3.A of the Directorates Strategic plan for 2016 to 2020 was that every member state was to have a national Plan in place targeting WHO NCD targets (European Commission, 2016). |
| **Evidence of implementation** | **Ireland**  **Healthy Ireland, A Framework for Improved Health and Wellbeing 2013 - 2025**  The 'Healthy Ireland' Framework outlines the Republic of Ireland’s response to contemporary issues in public health. Action 2.11 stipulates that government departments will develop a plan to increase physical activity. The Healthy Ireland Framework is overseen by the Cabinet Committee on Social Policy. The Cabinet Committee is chaired by An Taoiseach (Healthy Ireland 2013). Furthermore, increasing the "proportion of the population undertaking regular physical activity" is listed as a key performance indicator (Healthy Ireland, 2013 p34).    **Get Ireland Active! National Physical Activity Plan for Ireland**  The National Physical Activity Plan (NPAP) fulfils the requirements of action 2.11 of the Healthy Ireland Framework. The NPAP Implementation Group is co-chaired by the department with responsibility for health and the department with responsibility for sport (Healthy Ireland, 2016) (see statements Leadership 01 and Governance 04).    **National Sports Policy 2018 – 2027**  The National Sports Policy has three overriding 'High Level Goals': 'increased participation', 'more excellence' and 'improved capacity'. There are several targets, or 'Key Performance Indicators' listed for gauging success in achieving these goals. Indicators of success for the first goal include increasing the proportion of adults actively participating in recreational sport for 43% to 50% over the lifetime of the policy and increasing the number of children regularly playing sport (Department of Transport, Tourism and Sport, 2018b). This target was subsequently made more ambitious by the 2020 Programme for Government which targets 60% participation (Department of An Taoiseach, 2020a). |

| **L03**  **Priorities are given to reduce inequalities in relation to inactivity related non-communicable diseases in the comprehensive plan (above).** | |
| --- | --- |
| **Definitions and scope** | - Frameworks, strategies or implementation plans specify aims, objectives or targets to reduce inequalities including taking a preventive approach that addresses the social and environmental determinants of health. - Frameworks, strategies or implementation plans identify vulnerable populations or priority groups. - Implementation plans specify policies or programs that aim to reduce inequalities for specific population groups. - Excludes priorities to reduce inequalities in secondary or tertiary prevention (Food EPI Evidence Document – LEAD5). |
| **International best practice examples (benchmarks)** | **Spain**  **Plan Integral Para La Actividad Física Y El Deporte**  The Plan Integral Para La Actividad Física Y El Deporte 2010–2020 (Comprehensive Plan for Physical Activity and Sport 2010–2020) contains 100 indicators or ‘measures’ across 15 programmes (WHOROE and European Commission 2021). The fourth of these programmes is entitled “Actividad Física Y Deporte Para las Personas con Discapacidad (Physical Activity and Sports for People with Disabilities)”. The seventh of these programmes is entitled “Actividad Física y Deporte para la Inclusión Social (Physical Activity and Sports for Social Inclusion)”. The nineth of these programmes is entitled “Igualdad Efectiva entre Mujeres y Hombres (Effective Equality between Women and Men)”. |
| **Context e.g. EU action / regulation** | **WHO**  **Global Action Plan on Physical Activity**  Proposed sub action for member states 4.1.2: Strengthen current, and where necessary develop new, national and subnational action plans on physical activity and sedentary behaviour which align with recommendations in global and regional guidance, and maximize policy coherence and synergies with relevant priorities across key sectors including, but not limited to, transport, urban planning, health, social care, education, tourism, and sports and recreation (WHO, 2018).  **EU Action**  **Council Recommendation of 26 November 2013 on promoting health-enhancing physical activity across sectors**  In 2013, the Council of the European Union issued four recommendations to member states. The first recommendation extorts member states to utilise a cross sectoral approach in developing HEPA policies (Council of the European Union, 2013). |
| **Evidence of implementation** | **Ireland**  **Healthy Ireland, A Framework for Improved Health and Wellbeing 2013- 2025**  The 'Healthy Ireland' Framework (2013) identifies the following groups as priority targets for funding:   - Disadvantaged communities - Disadvantaged men and women - Disadvantaged families, including one parent families - Children and young people - People with disabilities, including people mental health issues - Unemployed young people and adults - Traveller and Roma communities - New communities, asylum seekers and refugees - LGBTI communities - Homeless people - Older people - People with chronic health conditions     The document 'Healthy Ireland Strategic Action Plan 2021–2025' updates the 'Healthy Ireland' Framework (Healthy Ireland 2013, 2021). The actions of the original 'Healthy Ireland' Framework are organised into six ‘themes’ titled “Governance and Policy”, “Partnerships and Cross-Sectoral Work”, “Empowering People and Communities”, “Health and Health Reform”, “Research and Evidence” and “Monitoring, reporting and evaluation”. In the Strategic Action Plan, themes four, five and six are revised to “Sláintecare Health Reform”, “Research, Evidence, Monitoring, Reporting and Evaluation” and “Reducing Health Inequalities”. The revisions to themes five and six were introduced to attend to “pressing need to address health inequalities and place a greater emphasis on promoting Healthy Ireland amongst disadvantaged and harder to reach communities" (Healthy Ireland, 2021 p5).  Theme six contains six “strategic actions” and nine “Implementation actions”. |

| **L04**  **There are clearly defined, evidenced informed population physical activity guidelines for all age groups and for people living with non-communicable diseases, pregnant women, and people with disabilities.** | |
| --- | --- |
| **Definitions and scope** | - Evidence-informed includes extensive review of up-to-date research and mechanisms to seek expert input (see definitions above). - Evidence includes ways physical activity guidelines have been used to develop/implement policies to improve physical activity (adapted from Irish Food EPI physical copy – LEAD3). |
| **International best practice examples (benchmarks)** | **France**  The Government of France’s guidelines (*Actualisation des repères du PNNS - Révisions desbrepères relatifs à l’activité physique et à la sédentarité*) are based on the WHO 2010 guidelines and contains recommendations for children (<5 years), Adults (18–64 years), Older adults(≥ 65 years), Frail and very elderly adults (≥ 85 years), Pregnant and breastfeeding women, People with disabilities, People with chronic diseases, Children and adolescents (5–17 years), and Postmenopausal women [WHO Europe physical activity fact sheet, 2018]. |
| **Context e.g. EU action / regulation** | **WHO**  **Global Action Plan on Physical Activity**  Proposed sub action for member states 4.1.4: Review and, where needed, adopt or update national physical activity and sedentary behaviour guidelines for all ages, and disseminate through tailored resources adapted to target audiences, settings and local context (WHO, 2018).  **EU**  **2013 Council recommendations**  Indicator 1: National recommendation on physical activity for health (yes/no) (Council of the European Union, 2013). |
| **Evidence of implementation** | **Ireland**  **The National Guidelines on Physical Activity for Ireland**  The following guidelines for physical activity were issued by the Department of Health and Children and the Health Service Executive in 2009:   - "All children and young people [aged 2 - 18] should be active, at a moderate to vigorous level, for at least 60 minutes every day. Include muscle-strengthening, flexibility and bone-strengthening exercises 3 times a week" (Department of Health and Children and Health Service Executive, 2009 p10). - Adults (aged 18–64) should engage in "at least 30 minutes a day of moderate activity on 5 days a week (or 150 minutes a week)." (Department of Health and Children and Health Service Executive, 2009 p13). - Older people (aged over 65) should engage in "least 30 minutes a day of moderate intensity activity on five days a week, or 150 minutes a week. Focus on aerobic activity, muscle-strengthening and balance" (Department of Health and Children and Health Service Executive, 2009 p15). - Adults with disabilities should "Be as active as your ability allows. Aim to meet adult guidelines of at least 30 minutes of moderate-intensity activity on 5 days a week" (Department of Health and Children and Health Service Executive, 2009 p16).     **Get Ireland Active! The National Physical Activity Plan for Ireland**  Action 18 of the National Physical Activity Plan (NPAP) states that new guidelines on physical activity will be developed for children aged 0 - 5 years of age (Healthy Ireland, 2016). |

DOMAIN 10 – Governance

**There are government structures in place to ensure transparency and accountability, and encourage broad community participation when developing and implementing policies and actions to create healthy physical activity environments and improve population physical activity.**

| **G01**  **There are reliable procedures to restrict commercial influences related to physical activity environments where there are conflicts of interest with improving population physical activity levels (e.g., restricting lobbying influences that limit physical activity opportunities).** | |
| --- | --- |
| **Definitions and scope** | - Includes government policies, guidelines, codes of conduct or other mechanisms to guide actions and decision-making by government employees, for example conflict of interest declaration procedures. - Includes procedures to manage partnerships with private companies or peak bodies representing industries that are consulted for the purpose of developing policy, for examples committee procedural guidelines or terms of reference. - Includes publicly available, up-to-date registers of lobbyist and/or their activities (Food EPI Evidence Document – GOVER1). |
| **International best practice examples (benchmarks)** | **USA**  Mandatory and publicly accessible lobby registers exist at the federal level, as well as in nearly every state. Financial information must be disclosed, and the register is enforced through significant sanctions. A number of pieces of legislation uphold compliance with the register including Lobbying Disclosure Act of 1995 and the Honest Leadership and Open Government Act 2007.  **New Zealand**  The State Services Commission has published Best Practice Guidelines for Departments Responsible for Regulatory Processes with Significant Commercial Implications. They cover the development and operation of a regulatory process and include specific references to principles around stakeholder relationship management.  **Australia**  The Australian Public Service Commission’s Values and Code of Conduct includes a number of relevant sections such as the Conflict of Interest, Working with the Private Sector and other Stakeholders and the Lobbying Code of Conduct. (Food EPI Evidence Document – GOVER1). |
| **Context e.g. EU action / regulation** | **WHO**  **Global Action Plan on Physical Activity**  Proposed sub action for member states 2.4.5: Strengthen the implementation of market restrictions on unhealthy food and non-alcoholic beverages in and around parks, other open public spaces, schools and sports facilities to reduce exposure to the marketing of foods high in fat, salt and sugar, consistent with previous commitments and recommendations of the Commission on Ending Childhood Obesity (WHO, 2018).  **EU Transparency Register (European Commission)**  The European Commission has set up a database of special interest groups whose goal is to influence policy and law making at the European institutions. Registrants are bound by a Code of Conduct (Annex 3 of the Inter Institutional Agreement) which set out rules for all registrants and establishes the underlying principles for standards of behaviour in all relations with the EU institutions (Europa, 2014a).  Commissioners, their cabinet members and Directors-General publish information on meetings held with organisations or self-employed individuals (European Commission). Meetings related to policy-making and implementation in the EU can only take place if the interest representatives are registered in the EU Transparency Register. |
| **Evidence of implementation** | **Ireland**  **Regulation of Lobbying Act 2015**  The Regulation of Lobbying Act 2015 was signed into law in March 2015. The purpose of the Act is to provide a web-based register of lobbying activities and to provide information to the public on the identities of those communicating with public officials on public policy, legislative matters or prospective decisions. To support the Act’s objectives of increasing transparency and proper conduct of lobbying activities, the 'Code of Conduct for Persons Carrying on Lobbying Activities' was established. As its name implies, its purpose is to govern the behaviour of persons carrying out lobbying activities. The provisions of the Act can apply to employers, to representative or advocacy bodies, to professional lobbyists or to third parties who are being paid to communicate on behalf of a client or other person; and significantly, to any person communicating about the development or zoning of land (Standards in Public Office Commission, 2019).    The register of lobbyist is available at: <https://www.lobbying.ie/>  **Public Health (Alcohol) Act 2018**  The Public Health (Alcohol) Act of 2018 places restrictions on the advertising of alcoholic products. Section 14 of the Act prohibits the advertising of alcohol at certain locations including parks or playgrounds owned by local authorities and schools. Section 15 of the Act prohibits the advertising of alcohol products at sports events. Section 16 of the Act prohibits the sponsorship of certain events, including events aimed at children, by alcohol brands. |

| **G02**  **There are procedures in place for using evidence in the development of physical activity policies.** | |
| --- | --- |
| **Definitions and scope** | - Includes policies, procedures or guidelines to support government employees in the use of evidence for policy development including best practice evidence review methodology (including types and strength of evidence needed) and policy implementation in the absence of strong evidence (where the potential risk or harms of inaction are great). - Includes policies, procedures or guidelines that stipulate the requirements for the establishment of a scientific or expert committee to inform policy development. - Includes the use of evidence-based models, algorithms and tools to guide policy development or within policy to guide implementation (e.g. nutrient profiling model). - Includes government resourcing of evidence and research by specific units, either within or across government departments. |
| **International best practice examples (benchmarks)** | **Australia**  The National Health and Medical Research Council Act 1992 (NHMRC Act) requires NHMRC to develop evidence-based guidelines. These national guidelines are developed by teams of specialists following a rigorous nine-step development process. |
| **Context e.g. EU action / regulation** | **WHO**  **Global Action Plan on Physical Activity**  Action 4.3: Strengthen the national and institutional research and evaluation capacity and stimulate the application of digital technologies and innovation to accelerate the development and implementation of effective policy solutions aimed at increasing  Proposed sub action for member states 4.3.2: Identify and disseminate a set of national research priorities for physical inactivity and sedentary behaviour to strengthen the evidence base and inform national planning and implementation of policy actions.  Proposed sub action for member states 4.3.4: Within all government departments, strengthen a culture of innovation, evaluation and knowledge-sharing to ensure that research and practice-based evidence on physical activity and sedentary behaviour are widely accessible and can advance global, regional, national and subnational level policy implementation and effective use of limited resources (WHO, 2018).  **The Institute of Public Health**  The Institute of Public Health in Ireland have a Policy Team which support evidence-informed policy-making at all stages of the policy cycle. The activities of the Policy team include evidence synthesis/reviews, consultations, health impact assessments and evaluations. The policy team responds to public consultations to maximise the impact of policies outside the health sector. The Policy team contributes to government policies on obesity, tobacco, breastfeeding, healthy workplaces and child wellbeing as well as contributing to EU funded projects relevant to public health (Institute of Public Health, 2019). |
| **Evidence of implementation** | **Ireland**  **Healthy Ireland, A Framework for Improved Health and Wellbeing 2013- 2025**  The 'Healthy Ireland' Framework (2013) sets out ‘Better use of evidence’ as one of its seven 'Guiding Principles for Implementation’. The desire to ensure that actions within the framework are based on robust evidence is alluded to throughout the framework document.  The Framework for action section consists of six ‘themes’. Theme 5 is titled ‘Research and Evidence’. It contains six actions to build capacity for health and wellbeing research.  Appendix 1 of the document consist of an “Overview of Literature on Health, its Determinants and Evidence to Address the Risks”.    **Get Ireland Active! The National Physical Activity Plan for Ireland**  The National Physical Activity Plan for Ireland (NPAP) is divided into eight ‘thematic action areas’.  Thematic area seven is entitled ‘Research, Monitoring and Evaluation’. Within this area, action 52 states that a research programme to inform physical activity policies and programmes will be established.  The latest NPAP implementation report names several studies and surveys as evidence of activities in fulfilling this action. These are:  The Children’s Sport Participation and Physical Activity Study (CSPPA, see Monitoring and Intelligence 01 for details). The Irish Physical Activity Research Collaboration (I-PARC, see Platforms for Interaction 01 for details). Two interventions by the Economic and Social Research Institute (ESRI) seeking to provide behavioural insights into increasing sport and physical activity participation in groups of lower socio-economic status. The Move for Life (MfL) study, a physical activity study of persons over 50 examining the effectiveness of a peer mentoring intervention. The completion of HEPA PAT version 2 by the NPAP implementation group. The Institute of Public Health’s All Island Physical Activity and Ageing group. This group connects academics and policymakers via triennial meetings (Healthy Ireland et al., 2021).    **A Healthy Weight for Ireland, Obesity Policy and Action Plan 2016-2025**  The Obesity Policy and Action Plan (OPAP) contains ten steps. Step ten is: “Develop a multi-annual research programme that is closely allied to policy actions”. Contained within step ten are 11 actions.  Action 10.3 is “Develop and implement a national physical activity surveillance system”.  Action 10.7 is “Enhance the research capabilities, training and capacity to provide for knowledge translation”.  Action 10.8 is “Develop an obesity ‘knowledge translation’ programme to inform the Action Plan”.    **National Sport Policy 2018 – 2027**  The “Values Statement” of NSP lists being “evidence-led and outcomes-focused” as a core value underpinning the policy (p.19). Scientific studies are cited in the text of the policy document. For example: the CSPPA study (see Monitoring and Intelligence and for other surveys that may be used in the development of physical activity policies) is cited as support for the statement that there is “scope for improvement in our PE system around the time allocation, facility availability and monitoring of outcomes for children” (DTTS, 2018 p28).  **Participation Plan 2021-2024 Increasing Participation in a Changing Ireland**  The participation plan, which builds on NSP, contains a theme with five actions titled ‘Evidence Informed’. The plan pledges to use research to use research to develop investment strategies for participation initiatives (action 13), understand the market for participation in sport (action 14), use behavioural research to understand non-participation (action 15), to develop tools for the assessment of impact of publicly funded facilities and initiatives (action 16) and grow Sport Ireland’s Evaluation function (action 17) (Sport Ireland, 2021a).  **National Sports Policy 2018 – 2027 Sports Action Plan 2021 - 2023**  Section 7 of the Sport Action Plan is titled ‘Research and Evaluation’ Action 7.1 states that a research strategy, and research data repository will be developed (Department of Tourism, Culture, Arts, Gaeltacht, Sport and Media, 2021).  **Sport Ireland’s Research Strategy 2021 – 2027**  The Sport Ireland research strategy contains 26 actions. The first action commits to monitoring rates and types of sport participation to inform policy decisions (Sport Ireland 2021d). |

| **G03**  **The government ensures access to and regular dissemination of physical activity guidelines and key documents to the public.** | |
| --- | --- |
| **Definitions and scope** | - Includes policies and procedures to guide the timely, online publishing of government budgets, performance reviews, audits, evaluation reports or the findings of other reviews or inquiries. - Includes ‘freedom of information’ legislation and related processes to enable the public access to government information on request, with minimal restrictions and exemptions. - Includes policies or procedures to guide the timely, online publishing of population health data captured /owned by government. |
| **International best practice examples (benchmarks)** | **France**  The French Agency for Food, Environmental and Occupational Health Safety (ANSES) updates and disseminations the national sedentary behaviour and physical activity guidelines. (Director General of Agence Nationale de Sécurité Sanitaire de l'Alimentation, 2015; Agence Nationale de Sécurité Sanitaire de l'Alimentation, 2020). |
| **Context e.g. EU action / regulation** | **WHO**  **Global Action Plan on Physical Activity**  GAPPA Action 4.1: Strengthen policy frameworks, leadership and governance systems, at the national and subnational levels, to support implementation of actions aimed at increasing physical activity and reducing sedentary behaviours, including multisectoral engagement and coordination mechanisms; policy coherence across sectors; guidelines, recommendations and actions plans on physical activity and sedentary behaviour for all ages; and progress monitoring and evaluation to strengthen accountability.  Proposed sub action for member states 4.1.4: Review and, where needed, adopt or update national physical activity and sedentary behaviour guidelines for all ages, and disseminate through tailored resources adapted to target audiences, settings and local context (WHO, 2018). |
| **Evidence of implementation** | **Ireland**  **Get Ireland Active! The National Physical Activity Plan for Ireland**  The national guidelines contain a section on promoting the guidelines. Partners supporting the promotion of physical activity were identified as: HSE health promotion departments, local sport partnerships, the Irish Heart Foundation, National Governing Bodies of Sports and the CARA Adapted Physical Activity Centre (Dept of Health and Children, 2009). |

| **G04**  **The government fosters the cooperation and coordination of all sectors to align with strategic plans to improve the physical activity environment, and where appropriate, promotes civil society participation to develop and implement these plans.** | |
| --- | --- |
| **Definitions and scope** | - Excludes structures for cross-departmental governance (covered by Platforms for Interaction 01). |
| **International best practice examples (benchmarks)** | **Norway**  **The Action Plan on Physical Activity 2005-2009**  The Norwegian action plan was developed was a collaborative process involving various NGOs. Members of the reference group included the Norwegian Diabetes Association, the Norwegian Federation of Organisations of Disabled People, the Norwegian Confederation of Trade Unions, the Confederation of Norwegian Enterprise and the Confederation of Vocational Unions.  The product of this development process includes measures that are implemented by civil society organisations. For example, measure no.23 allocates funds to NGOs that engaged in “low threshold” activities such as organising “walking buses" to school for children or organising outdoor recreation activities to promote mental health (Belander et al., 2011). |
| **Context e.g. EU action / regulation** | **WHO**  **Global Action Plan on Physical Activity**  Proposed sub action for member states 4.1.1: Initiate or strengthen, as appropriate, a high-level national multisectoral coordination committee to provide leadership, strategic planning and oversight of implementation and monitoring of national policy actions on physical activity and sedentary behaviour, ensuring appropriate representation from all relevant areas and levels of government, as well as nongovernmental stakeholders and the community (WHO, 2018). |
| **Evidence of implementation** | **Ireland**  **Get Ireland Active! The National Physical Activity Plan for Ireland**  The eighth action areas within the National Physical Activity Plan (NPAP) is titled "implementation through partnership".  The policy states that stakeholders from the "children, education, environment, health, sport, transport and academic sectors" are to be given a leadership role in the implementation of the plan (Healthy Ireland, 2016 p34).  A cross sectoral working group was established to develop NPAP.  The members of the working group listed (in appendix two of NPAP) include representatives from the following organisations: The Department of Health, and the Department of Transport, Tourism and Sport (co-chairs), the Health Service Executive, the Department of Children and Youth Affairs, Fingal County Council, the Federation of Irish Sport, Sport Ireland, the Department of Health and Skills and Dublin City University.  Action 56 of NPAP states that a cross sectional group will be established to oversee the continued implementation of the plan. The membership of the implementation group changed over the lifetime of the policy. Academic partners include the University of Limerick and University College Dublin and the Department of Rural and Community Development joined the group in 2019 (Healthy Ireland, 2021). Furthermore, there was a change in government and relatedly organisation rearrangements of government departments in 2020. The new Department of Tourism, Culture, Arts, Gaeltacht, Sport and Media is responsible for the role of the former Department of Transport, Tourism and Sport; the new Department of Children, Equality, Disability, Integration and Youth took the responsibilities of the former Department of Children and Youth Affairs; the Department of Education took the responsibilities of the former Department of Education and Skills; the department of Housing, Local Government and Heritage took the responsibilities of the former Department of Housing, Planning and Local Government.  Partners are listed for each of NPAPs 60 actions (Healthy Ireland 2016). For example: action 5 (“Develop a publicly accessible national sports / recreation facilities and amenities directory”) lists 3 government departments, local authorities, local sport partnerships and National Governing Bodies as partners to the lead (the HSE) (Healthy Ireland, 2016, p16).  **National Sports Policy 2018 - 2027**  Action 8 of the NSP states that Sport Ireland will establish an initiative to support local authorities to develop local sports plans to support sport participation locally (see Sport 01).  **Participation Plan 2021-2024 Increasing Participation in a Changing Ireland**  Actions 5, 6 and 7 of the participation plan (theme ‘local action plans’) link in with actions 8 and 9 of NSP and state that supports for local sport plans development will include the development of templates for the plans and annual meetings to support development and implementation (Sport Ireland, 2021a).  **National Sports Policy 2018 – 2027 Sports Action Plan 2021 - 2023**  Action 6.2 of the Sports Action Plan states that: “Sport Ireland will work with Local Authorities nationally to develop a framework to support the development and implementation of Local Sports Plans, ensuring in particular collaboration with LSPs and NGBs.” (Department of Tourism, Culture, Arts, Gaeltacht, Sport and Media, 2021; p25). |

DOMAIN 11 – Monitoring and Intelligence

**There is regular monitoring of population physical activity levels and physical activity environments, systematically linked to the regular monitoring of physical inactivity related non-communicable diseases. Ideally, monitoring should be consistent over time, integrated and occur annually, with more extensive surveys at least every five years (e.g. to allow data analysis across all jurisdictions, priority groups). Additionally, policies and major programmes should be evaluated regularly.**

| **MI01**  **There is regular monitoring of physical activity levels across the life-course based on representative samples, against guidelines/standards/targets.** | |
| --- | --- |
| **Definitions and scope** | - ‘Regular’ is considered to be every five years or more frequently (Food EPI Evidence Document – MONITOR2). |
| **International best practice examples (benchmarks)** | **Finland**  The survey Health Behaviour and Health among the Finnish Adult Population (Suomalaisen aikuisväestön terveyskäyttäytyminen ja terveys - AVTK) measures physical activity in the adult population (ages 18 – 64) on an annual basis  The Health Behaviour and Health among the Finnish Elderly (Eläkeikäisen väestön terveyskäyttäytyminen ja terveys - EVTK) measures physical activity in the elderly population (ages 65 – 84) on an bi annual basis  **United Kingdom**  The Active Lives Adult Survey focuses on people aged 16 and over. The survey collects responses from around 175 000 randomly selected people, no less than 500 from each local authority1. There is also a survey for children and young people (age 5 – 16) the Active Lives Children and Young People Survey (Sport England, 2021) |
| **Context e.g. EU action / regulation** | **WHO Global Action Plan on Physical Activity:**  Enhance data systems and capabilities at the national and, where appropriate, subnational level, to support: regular population surveillance of physical activity and sedentary behaviour, across all ages and multiple domains; development and testing of new digital technologies to strengthen surveillance systems; (…) (action 4.2) (WHO, 2018).  **WHOROE**  **Physical activity strategy for the WHO European Region 2016–2025**  Member States should work towards consolidating, adjusting and extending existing national and international systems for the surveillance of physical activity with the adequate levels of disaggregation. Member States recall the commitment to the WHO global monitoring framework for the prevention and control of noncommunicable diseases, which contains specific physical activity indicators and a related target (…). (objective 5.1) (WHOROE, 2016).  **EU Council recommendations:**  Monitoring and surveillance of physical activity and sedentary behaviour (indicator 10) (Council of the European Union, 2013). |
| **Evidence of implementation** | **Ireland**  **Children and Young People**  **Childhood Obesity Surveillance Initiative**  Ireland participates in the Childhood Obesity Surveillance Initiative (COSI). COSI is a collaborative study with 35 participating countries. COSI measures trends related to overweight and obesity in children aged 6 to 10 years via their schools. Measurements are taken every three years. Measures reported in COSI include participation in structured physical activities outside of schools (stratified by hours per week) and vigorous physical activity reported by family (stratified by hours per day) (Mitchell et al., 2020).  **Health Behaviour in School-aged Children**  Ireland participates in the WHO’s Health Behaviour in School-aged Children (HBSC) research study. The HBSC study aims to provide insight into the health behaviours of young people (aged between 9 and 18 years). Measurements are taken every four years from a nationally representative sample (Gavin et al., 2020). The HBSC study reports on the proportion of children participating in vigorous exercise 4 or more times per week.    **Growing Up in Ireland**  The Growing Up in Ireland (GUI) survey is a longitudinal interview study of children in Ireland. GUI follows two cohorts of children (the ‘child’ cohort of 8 500 subjects born in 1998 and the ‘infant’ cohort of 11 000 subjects born in 2008). It is funded by the department with responsibility for children and young people and is managed by that department in association with the Central Statistics Office (CSO). The research examines a wide variety of issues concerning children’s development, including physical exercise (Growing Up in Ireland, n.d.).  For the child cohort, data is available for 4 waves of data collection: initial data collection at 9 years of age then subsequent waves at 13 years, 17 or 18 years and 20 years.  For the ‘infant’ cohort, data is available for 5 waves of data collection: initial data collection at 9 months of age, then subsequent waves at 3 years, 5 years, 7 or 8 years and 9 years.    **Children’s Sport Participation and Physical Activity Study (CSPPA)**  The cross-sectional Children’s Sport Participation and Physical Activity Study was conducted in 2010 and 2018 and funded by Sport Ireland. It surveyed participation in physical activity and sport among children aged 10 to 18. An all-Ireland survey, the 2018 iteration surveyed 1103 pupils at the primary school level and 3594 students at the post primary level in the Republic.  Data was stratified by gender to shed light on gender inequalities. Data on socioeconomic inequalities were measured by the Family Affluence Scale II (FAS) and disability status was assessed using the Child Functioning Module questionnaire.  A subsample of 1325 participants provided the following physical health measures: Cardiorespiratory fitness, blood pressure, waist circumference and body mass index. The 2018 survey found that 13% of children were meeting National Physical Activity Guidelines of at least 60 minutes of MVPA per day (Woods et al., 2018).    **Adults**  **The Irish Sports Monitor**  The Irish Sports Monitor collects data on the proportion of Irish people who are regularly involved in sporting activity.   The survey is undertaken biennially (Sport Ireland and Ipsos MRBI, 2019).    **Health Ireland Survey**  The annual Healthy Ireland Survey monitors the proportion of the Irish population achieving the national physical activity guidelines.  The survey is an annual interview, administered face-to-face, commissioned by the Department of Health. The sample is representative of the population aged 15 and older living in Ireland. Survey data plays a number of roles, including supporting the Department in on-going engagement and awareness-raising activities in the various policy areas and supporting better understanding of policy priorities (Healthy Ireland, 2018).    **European Health Interview Survey / Irish Health Survey**  The European Health Interview Survey is conducted in Ireland as the Irish Health Survey. The survey collects data on the physical activity of persons aged 15 years and over amongst other health behaviours. The indicators measuring physical activity behaviours are the following: The proportion of persons who walk to get to and from places, the proportion of persons who cycle to get to and from places, the proportion of persons who do sports, fitness or recreational physical activities, the proportion of persons who do muscle strengthening activities (Central Statistics Office, n.d.).  **Older Adults**  **The Irish Longitudinal Study on Ageing**  The Irish Longitudinal Study on Ageing (TILDA) survey was initiated in 2009 drawing data from a representative cohort of 8 504 people over the age of 50. Four subsequent waves were completed in 2012, 2015, 2016, and 2018. A sixth waves is currently underway. Data on health behaviours is collected as part of TILDA. Health behaviours measured include physical activity which is measured using the short form International Physical Activity Questionnaire (IPAQ).  Furthermore, data assessing health status was collected in the first and third waves and is being collected in the sixth wave. |

| **MI02**  **There is regular monitoring of physical activity environments across all 8 policy domains (e.g., walkability, built environment).** | |
| --- | --- |
| **Definitions and scope** | - ‘Regular’ is considered to be every five years or more frequently - Physical activity environments are “places where people are, or can be, physically active”. They include spaces created or modified by people such as schools, parks, workplaces and transportation systems (Sallis; 2009; pS87). |
| **International best practice examples (benchmarks)** | **Denmark**  **Prevention package on physical activity**  The Prevention package on physical activity includes cross-sectoral policy to improve the physical environment and other measures such as prevention initiatives for citizens. It is used by the 98 Danish municipalities and comprises 26 recommendations, e.g. with regards to city planning, access to sport facilities as well as policies in schools and day care centres. The effect of the package is monitored every second year (WHO/Europe 2018).  **Netherlands**  **New indicator for national monitoring of sport and exercise**  In 2021, a new indicator was defined to monitor the “friendliness” of the living environment for physical activity. This indicator is based on four sub-indicators: the numbers of public sports accommodations, play areas and recreational space (blue and green zones) and the proximity of services (WHO/Europe 2021). |
| **Context e.g. EU action / regulation** | **WHO**  **WHO Global Action Plan on Physical Activity:**  Enhance data systems and capabilities at the national and, where appropriate, subnational level, to support: (…) development of monitoring systems of wider sociocultural and environmental determinants of physical activity (…) (action 4.2) (WHO, 2018). |
| **Evidence of implementation** | **Ireland**  **Childhood Obesity Surveillance Initiative (Education)**  COSI collects information on the physical activity environment in schools every three years. Statistics reported in the latest COSI report state that in Ireland: 100% of reporting schools had outdoor play areas, 77% had an indoor gym 100% had a school curriculum which included PE lessons and 30.4% of schools organised physical activities for children of all grades outside school hours (with a further 34.1% for some grades) (Mitchell et al., 2020 p20-21).  **Lifeskills Survey (Education)**  The Lifeskills Survey seeks to inform policy direction every three years (Department of Education and Skills, 2017b). Recent surveys found differences in the proportion of primary and post primary schools reporting providing the recommended levels of Physical Education (see indicator Education 01).  **Sport Ireland’s National Trials Register (Community)**  Sport Ireland maintains a register of trails in Ireland with over 920 listed trails across Ireland. Trails on the register are assessed for safety issues and assigned a rating for safety on a five-category rating scale. According to the Sport Ireland website ratings are:   1. Accredited: trails which meet the Management Standards for Recreational Trails 2. Partially Accredited: trails that may have some safety issues and /or other work to complete in order to fully meet the standard 3. Non-Accredited Trails: trails that meet basic requirements in relation to access and safety, but otherwise do not meet the requirements of the Management Standards for Recreational Trails 4. Closed: trails that do not meet the basic requirements for access and safety, or have other significant issues 5. Under Development: new trails which are under development, or existing trails which are being extensively re-redeveloped   The register is searchable via the Sport Ireland website. Lower tier trails are not displayed in the search results.  **National Sports Policy 2018 – 2027 (Sport)**  Action 18 of NSP states that period reviews of sport facilities will be conducted and that an up-to-date national database of sports facilities will be maintained and will be made accessible to the public via the web (DTTS, 2018).  **National Sports Policy 2018 – 2027 Sports Action Plan 2021 - 2023**  Action 2.4 of Sports Action Plan states that a publicly accessible database of sport facilities and recreational amenities will be developed. Elsewhere in the action plan it states that work has commenced on developing a database of sport facilities (DTCAGS, 2021). |

| **MI03**  **Physical activity monitoring is systematically linked to the regular monitoring of non-communicable diseases and their related inequalities.** | |
| --- | --- |
| **Definitions and scope** | - ‘Regular’ is considered to be every five years or more frequently |
| **International best practice examples (benchmarks)** | **Germany**  **Gesundheit in Deutschland aktuell (GEDA)**  The GEDA study is part of Germany’s health monitoring that is conducted by the Robert Koch Institute, a federal government agency and research institute. GEDA integrates the questionnaire of the European Health Interview Survey (EHIS) that systematically links physical activity monitoring to the regular monitoring of non-communicable diseases (see “context” below). Physical activity data are analysed by gender, age group and level of education in order to identify socioeconomic inequalities. |
| **Context e.g. EU action / regulation** | **EU**  **The European Health Interview Survey (EHIS)**  The European Health Interview Survey (EHIS) includes questions on physical activity and other health determinants such as height and weight, fruit and vegetable consumption, smoking and alcohol consumption. Besides this module on health determinants, it includes three other modules on background variables on demography and socio-economic status (e.g., sex, age, education, labour status), the health status (e.g., self-perceived health, chronic conditions, limitation in usual activities, disease-specific morbidity, physical and sensory function limitations) and health-care use (e.g., hospitalisation, consultation, unmet needs, use of medicines, preventive actions). EHIS is run every five years and targets the population aged at least 15 and living in private households.  **WHO**  The WHO NOPA database compiles information to monitor progress on physical activity and obesity, as well as nutrition and diet. The Country Information contains surveillance data, policy documents, action to implement policy and examples of good practice in programmes and interventions.  **OECD**  **Health at a Glance**  A collaboration with the European Commission’s Directorate General for Health and Food Safety (DG Sante) the Organisation for Economic Co-operation and Development’s (OECD’s) biennial publication (even years) *Health at a Glance* contains information on a range of indicators including indicators reflecting health status (for example: indicators reflecting life expectancy across EU member states), risk factors (for example: indicators reflecting smoking levels), health expenditure (for example; indicators reflecting expenditure per capita), quality of care (for example: indicators reflecting survival for lung cancer) and accessibility (for example: indicators reflecting Unmet health care need).  Physical activity-related indicators are classified under risk factors. The 2020 Health at a glance report displays national data on “Share of 11- and 15-year-olds meeting WHO recommended daily physical activity 2018” and “Trends in physical activity among 11-, 13- and 15-year-olds 2006 –2018" (OECD and EU, 2018). |
| **Evidence of implementation** | **Ireland**  **Healthy Ireland Survey**  The annual Healthy Ireland Survey contains a physical activity module every 3 to 4 years. The physical activity component monitors the proportion of the Irish population achieving the national physical activity guidelines. The data reported from the survey is stratified by age and sex. The most recent iteration of the Healthy Ireland Survey that included a physical activity component was published in 2019. (Healthy Ireland, 2019).  **Irish Sport Monitor**  The Irish Sports Monitor contains the following item on health: “Do you have any long-term illness, health problem or disability that limits your daily activities or work”. Response options are “yes” or “no” (Ipsos MRBI, 2019). |

| **MI04**  **There is regular research and evaluation of policies and major programmes to assess their effectiveness, process, and impact on achieving the goals of the physical activity and health plans.** | |
| --- | --- |
| **Definitions and scope** | - Includes any policies, guidelines, frameworks or tools that are used to determine the depth and type (method and reporting) of evaluation required - Includes a comprehensive evaluation framework and plan that aligns with the key preventive health or nutrition implementation plans - The definition of major programs and policies is to be defined by the relevant government department - Evaluation should be in addition to routine monitoring of progress against a project plan or program logic (Food EPI Evidence Document – MONIT5). |
| **International best practice examples (benchmarks)** | **USA**  The National Institutes for Health (NIH) provide funding for rapid assessments of natural experiments. The funding establishes an accelerated review/award process to support time-sensitive research to evaluate a new policy or program expected to influence obesity related behaviours (e.g. dietary intake, physical activity, or sedentary behaviour) and/or weight outcomes, in an effort to prevent or reduce obesity (US National Institutes of Health, 2016).  **Ireland**  Under the *‘Healthy Weight for Ireland, Obesity Policy and Action Plan 2016-2025’*, a new Obesity Policy Implementation Oversight Group (OPIOG) was established in October 2017 and a progress report on each recommendation in the *OPAP* is currently being finalised under the aegis of the OPIOG (Oireachtas, 2019). As set out in Healthy Ireland, integrated health and social impact assessments will be conducted on relevant policy areas to support other government departments in contributing towards the prevention of overweight and obesity. |
| **Context e.g. EU action / regulation** | **WHOROE**  **Physical activity strategy in the WHO European Region**  Member States may make supporting research a priority in order to strengthen the evidence base on effective and efficient interventions to promote physical activity and appropriate government policy instruments on physical activity, including intersectoral approaches (objective 5.2) (WHOROE, 2016).  **WHO Global Action Plan on Physical Activity:**  Enhance data systems and capabilities at the national and, where appropriate, subnational level, to support: (…) regular multisectoral monitoring and reporting on policy implementation to ensure accountability and inform policy and practice (action 4.2) (WHO, 2018). |
| **Evidence of implementation** | **Ireland**  **Healthy Weight for Ireland, Obesity Policy and Action Plan 2016-2025**  A progress report on each recommendation in the OPAP is currently being finalised by the OPIOG (Oireachtas, 2019). As set out in Healthy Ireland, integrated health and social impact assessments will be conducted on relevant policy areas to support other government departments in contributing towards the prevention of overweight and obesity.    **Get Ireland Active! National Physical Activity Plan for Ireland** Action 54 of NPAP pledges to "Develop a standardised evaluation framework for publicly funded programmes designed to increase physical activity levels" (Healthy Ireland, 2016 p.32).  The latest NPAP implementation plan notes that I-PARC is working to develop a standardised evaluation framework for physical activity in Ireland. It further states that Sport Ireland has delivered training on evaluation to all LSPs. Sport Ireland has also delivered impact evaluation training sessions to Cycling Ireland, Swim Ireland, Athletics Ireland and Ireland Active (Healthy Ireland et al., 2021).    **National Sport Policy 2018 - 2027**  The National Sport Policy (NSP) proposes that "Sport Ireland will develop standardised evaluation frameworks which will allow for the robust assessment of the impact of publicly funded facilities, programmes and interventions" (Department of Transport, Tourism and Sport, 2018 p104). Sport Ireland is currently evaluating the impact on physical activity of programmes offered by LSPs and of initiatives such as Sport Ireland “Active Cities Project”.  **National Sports Policy 2018 – 2027 Sports Action Plan 2021 - 2023**  Section 7 of the Sport Action Plan is titled ‘Research and Evaluation’ (see Governance 02). Action 7.2 states that Sport Ireland shall use the Irish Sport Monitor to “refine our understanding of the issues around participation / non-participation in sport among  marginalised groups”. Further action 7.2 states that Sport Ireland will “Undertake a Value for Money Review of the Sports Capital and Equipment Programme” (DTCAGSM, 2021; p26).    **Irish Physical Activity Research Collaboration**  The Irish Physical Activity Research Collaboration (I-PARC) is a collaboration between physical activity researchers and knowledge users (policymakers and physical activity practitioners). I-PARC has developed three toolkits for promoting sport and physical activity including an evaluation toolkit (I-PARC, n.d.). The I-PARC website is available at: [https://i-parc.ie](https://i-parc.ie/). |

| **MI05**  **Progress towards reducing health inequalities related to social and economic determinants of physical activity is regularly monitored.** | |
| --- | --- |
| **Definitions and scope** | - Monitoring of overweight and obesity and main diet related NCDs includes stratification or analysis of population groups where there are the greatest health inequalities including Indigenous peoples and socio-economic strata - Includes reporting against targets or key performance indicators related to health inequalities (Food EPI Evidence Document – MONIT6). |
| **International best practice examples (benchmarks)** | **New Zealand**  All annual Ministry of Health Surveys report estimates by sub-populations, in particular by ethnicity (including Maori and Pacific peoples), by age, by gender, and by New Zealand area deprivation. |
| **Context e.g. EU action / regulation** | **Irish Medical Organisation – Position Paper on Health Inequalities**   - The Irish Medical Organisation (IMO) highlights the social, economic and environmental factors affecting the health of their patients, factors recognised by all IMO doctor, whether working in a hospital, general practice, or public or community health setting. - Analysis of The All-Ireland Traveller Health Study as well as the Prevalence of Chronic Conditions Ireland, including the prevalence of chronic conditions in more deprived areas, were outlined in the Paper (Irish Medical Organisation, 2012). |
| **Evidence of implementation** | **Ireland**  **Healthy Ireland Survey**  The results of the 2018 Healthy Ireland Survey contains a chapter on health inequalities in Ireland. It will continue to monitor inequalities annually. The survey found that those in more deprived areas are less likely to rate their health as good or very good, and more likely to have a long-term health problem. People living in more deprived areas are also more likely to smoke and binge drink. Self-rated good health declines earlier for men in deprived areas (around the age of 55-64) than it does for both men in affluent areas and women in deprived areas. Women living in more deprived areas are more likely than those in affluent areas to continue smoking and binge drinking into their 50s (Healthy Ireland, 2018).  **Demographics modules of Surveys**  Surveys mention in Monitoring and Intelligence 01 contain demographics modules with different methods for measuring social determinant of health, CSPPA contains measures of family socioeconomic status. The Family Affluence Scale II which categories respondents into families of low, medium or high affluence was utilised in the most recent version of this survey (Woods et al., 2018). The Irish Sports Monitor contains a section with socio economic questions. This includes items relating to education, household income, sexual orientation and race (Ipsos MRBI, 2019). The GUI survey collects information on parental income, parental social class and parental education (O’Mahoney et al., 2021). |

DOMAIN 12 – Funding and Resources

**Government funding to support PA promotion and research is clearly identified, monitored and sufficient. It is aimed at improving population PA levels, creating active environments, counteracting non-communicable diseases and reducing inequalities.**

| **FR01**  **The budget spent on physical activity promotion across all policy domains is clearly identified and periodically monitored.** | |
| --- | --- |
| **Definitions and scope** | - Includes allocations to higher education - Includes research by government departments |
| **International best practice examples (benchmarks)** | No suitable examples identified. |
| **Context e.g. EU action / regulation** | **EU**  **2013 Council recommendations**  Indicator 5: Finding allocated specifically to HEPA promotion (Total funding / per capita / by gross domestic product at PPP per capita in Euros) (Council of the European Union, 2013). |
| **Evidence of implementation** | **Ireland**  **Health-Enhancing Physical Activity Policy Audit Tool (HEPA PAT)**  The Health-Enhancing Physical Activity Policy Audit Tool (HEPA PAT) is an assessment that describes country-level policy responses on physical inactivity. Policymakers in the Republic of Ireland have reported on the funding allocated on physical activity policy delivery in the republic. According to the HEPA PAT report, €5 million of recurrent funding was ringfenced for physical activity in the health sector €94 million in the sport sector, €0.4 million in the education sector, €2.65 million in the transport sector, €185 million in the environmental sector and €35 million in other sectors. The report notes that funding for physical activity is difficult to calculate for a variety of reasons (Woods et al., 2019).  **Local Government Expenditure**  According to documents on local authority budgets, €38 million was spent on “Leisure Facilities Operations” in 2020. A further €140 million was spent on “Outdoor Leisure Areas Operations” and €68 million was spent on “Community Sport and Recreational Development” (Department of Housing, Planning and Local Government, 2020, see next statement).  **National Sport Policy 2018 - 2027**  Action 43 of NSP states that investment by the state in sport will be in excess of €220 million by 2027. Action 45 states that funding to participation programmes will double in the same period (DTTS 2018). |

| **FR02**  **There is a sufficient proportion of total health spending assigned to population physical activity promotion.** | |
| --- | --- |
| **Definitions and scope** | - The definition excludes all one-on-one and group-based promotion (primary care, nursing services etc.) - Includes estimates for the budget allocated to the unit within the Department of Health that has primary responsibility for physical activity (adopted from Food EPI FUND1) |
| **International best practice examples (benchmarks)** | **Ireland**  **Government Expenditure Report 2018**  In 2018, the funding level available to health reached €16.2 billion. The 2018 allocation marked a record as the highest in the history of the State. From 2016 onwards, the Budget provided increased the annual allocation and on average provided an additional €431m annually to health. Despite this increase, the health sector continued to overspend and the supplementary funding provided ranged from €195m to around €645m (Government of Ireland, 2018).  **Germany**  **The Preventive Health Care Act (Präventionsgesetz)**  The law was adopted in 2015 and aims to improve health promotion and prevention in Germany. New structures and institutions were created to improve the cooperation between important stakeholders, most importantly the National Preventive Conference. In addition, a new mandatory Prevention Guideline for the German statutory health insurances defines physical activity promotion as an important action area for prevention measures. Statutory health insurances are obliged to invest at least 7 Euro annually per insured person in health promotion and prevention, resulting in a total of approximately 500 mio. Euro. Out of this, at least 300 mio. Euro need to be invested in settings such as childcare, schools, communities, workplaces and nursing care. Thanks to the Preventive Health Care Act, total investment for health promotion and prevention has increased by around 40 percent, from 1.27 billion Euro in 2012 to 1.8 billion Euro in 2017. |
| **Context e.g. EU action / regulation** | **WHO**  **Global Action Plan on Physical Activity**  Action 4.5: Strengthen financing mechanisms to secure sustained implementation of national and subnational action and the development of the enabling systems that support the development and implementation of policies aimed at increasing physical activity and reducing sedentary behaviour.  Proposed sub action for member states 4.5.1: Allocate long term budgets for physical activity (including for sustained national communications) by taking into account national targets and priorities set by the national strategy and action plan (WHO, 2018).  **EU Action**  In addition to the Public Health Programme, the EU provides other funds to promote investing in health as a broader means of achieving smart and inclusive growth. These funds are the European and Structural Investment Funds (ESIF) and the European Fund for Strategic Investments (EFSI) (European Commission). The health sector receives ESIF for investments through the European Regional Development Fund and the European Social Fund. From 2014-2020 more than €9 billion was foreseen in all Member States for health-related investments (European Commission, 2020). The EFSI in partnership between the Commission and the European Investment Bank, will provide a financial guarantee to the value of €315 billion over a three year period and with an extension up to €500 billion until 2020 (European Commission).  For the next Multiannual Financial Framework 2021-2027, the European Social Fund Plus Programme will be the main investment fund for investing in Health (European Commission). This programme merges existing funds like the Health Programme, the European Social Fund (ESF), the Youth Employment Initiative (YEI), the Fund for European Aid to the Most Deprived (FEAD) and the Employment and Social Innovation (EaSI) programme. For the Health strand, an amount of €413 billion will be available. |
| **Evidence of implementation** | **Ireland**  **Total Budget**  According to the resources provided online by the Department of Finance total government expenditure was €107.6 billion in 2021, €94.4 billion in 2020 and €77 billion in 2019 (Department of Finance and the Office of the Government Chief Information Officer, n.d.).  **Government Expenditure Reports**  The 2022 allocation to health is €22 billion. The government's latest expenditure report states that this allocation demonstrates the government’s commitment to the phased implementation of Sláintecare, a new universal healthcare model. The expenditure report further states that: "Healthy Ireland is being provided with €10m in additional funding and will implement a healthy weight campaign and an innovative coordinated approach to encouraging physical activity through Sport Ireland and the HSE." (Department of Public Expenditure and Reform, 2022 p111).  **Local Government Expenditure**  According to local authority budget reports, local authorities collectively spent €502 million on recreation and amenities and €5.6 billion in total in 2020. Therefore, recreation and amenities accounted for approximately 9% of local government spending in 2020. Included in the recreation and amenities subtotal there was €38 million spent on “Leisure Facilities Operations”, €140 million was spent on “Outdoor Leisure Areas Operations” and €68 million was spent on “Community Sport and Recreational Development”. The other items in recreation and amenities are “Operation of Library and Archival Service”, “Operation of Arts Programme” and “Agency & Recoupable Services” (Department of Housing, Planning and Local Government, 2020; see previous statement). |

| **FR03**  **A sufficient proportion of total research spending is assigned to population physical activity promotion.** | |
| --- | --- |
| **Definitions and scope** | - Includes allocations to higher education - Includes research by government departments |
| **International best practice examples (benchmarks)** | **United States**  **National Institutes of Health (NIH),**  In 2021, the total NIH budget was $42.9 billion. The spend on projects relating to PA was $1.3 billion. Therefore, PA related projects accounted for 3% of the spending on health research. |
| **Context e.g. EU action / regulation** | **WHO**  GAPPA Action 4.3: Strengthen the national and institutional research and evaluation capacity and stimulate the application of digital technologies and innovation to accelerate the development and implementation of effective policy solutions aimed at increasing physical activity and reducing sedentary behaviour.  Proposed sub action for member states 4.3.1: Strengthen government and nongovernment funding support for research on physical inactivity and sedentary behaviour with a priority to generate evidence to inform and accelerate the scaling up of national and subnational implementation and addressing identified research priorities.  Proposed sub action for member states 4.3.2: Identify and disseminate a set of national research priorities for physical inactivity and sedentary behaviour to strengthen the evidence base and inform national planning and implementation of policy actions.  **EU**  Governments are required to disclose data on Government Budget Allocation on Research and Development (GBARD) by the commission regulation no 995/2012, a regulation for implementing Decision No 1608/2003/EC. |
| **Evidence of implementation** | **Ireland**  **Healthy Ireland, A Framework for Improved Health and Wellbeing 2013- 2025**  The Healthy Ireland document contains six themes or categories of policy activity. Theme five is titled “Research and Evidence”. The “Research and Evidence” theme includes six actions including action 5.1 which promises a Healthy Ireland Research Plan. It also contains an action, 5.2, which promises that Healthy Ireland partners will collaborate with the Health Research Board (HRB) to implement a plan to increase health and wellbeing research capacity. The plan is to include actions addressing knowledge gaps and improving dissemination and implementation in the Republic (Healthy Ireland, 2013).  A second document Healthy Ireland Strategic Action Plan 2021–2025 was later published (Healthy Ireland, 2021). Theme five (“Research and Evidence”) and theme six (“Monitoring, Reporting and Evaluation”) were reconfigured to place greater emphasis on health inequalities. Themes five and six were combined into a single theme in Strategic Action Plan 2021-2025 (“Theme five: Research, Evidence, Monitoring, Reporting and Evaluation”) and a new theme was added (“Theme six: Reducing Health Inequalities”).  The six actions of theme five and the nine actions of theme six have been replaced by eight actions (with sub actions) in the new theme five. These actions no longer include references to a research plan or to a collaboration with the HRB to increase research capacity in the area of health and wellbeing.    **Get Ireland Active! The National Physical Activity Plan for Ireland**  NPAP contains eight “action areas” (similar to themes in the Health Ireland Framework). Action Area seven is entitled “Research, Monitoring and Evaluation”). “Research, Monitoring and Evaluation” includes six actions (Healthy Ireland, 2016).    **Health Research Board**  The HRB is an agency responsible for funding health research in Ireland (IPA, 2021). The HRB provided €49.5 million in research awards in 2018/2019 of which 40% was allocated to health services research (€19.9 million), 26% was allocated to clinical research (€13 million), 23% was allocated to applied biomedical research (€11.5 million) and 10% was allocated to population health sciences (€5 million) (Lynn and Hiney, 2021).    **Sport Ireland Research Funding Scheme**  In 2021, Sport Ireland piloted a research funding scheme. The fund is designated to support research activities that relate to actions in NSP, NPAP and the Sport Ireland Statement of Strategy. A total of €250 000 was distributed between 17 successful applications (Sport Ireland, n.d.). |

| **FR04**  **A secure funding stream is available for at least one statutory health promotion agency with an objective to improve population physical activity.** | |
| --- | --- |
| **Definitions and scope** | - Agency was established through legislation - Includes objective to improve population physical activity levels in relevant legislation, strategic plans or on agency website - Secure funding stream involves the use of a hypothecated tax or secure source (adopted from Food EPI FUND3) |
| **International best practice examples (benchmarks)** | **Australia**  The Victorian Health Promotion Foundation (VicHealth) was the world’s first health promotion, established by the Victorian Parliament as part of the Tobacco Act of 1987 (for the first 10 years through a hypothecated tobacco tax) through which the objectives of VicHealth are stipulated. VicHealth continues to maintain bipartisan support.  **Belgium**  The Flanders Institute for Healthy Living receives funds specifically for developing and supporting policy instruments, strategies and projects for HEPA promotion and reducing sedentary behaviour. Local and regional health organizations receive funding to disseminate the instruments, strategies and projects (WHO/Europe 2021). |
| **Context e.g. EU action / regulation** | **EU**  **Statutory health promotion agency**  At EU level there is a Steering Group on Health Promotion, Disease Prevention and Management of Non-Communicable Diseases (NCDs), which was set up in July 2018 (European Commission, 2018b). This Steering Group assists and advises the Commission on the coordination between Member States in addressing challenges caused by NCD’s, the selection of best practices regarding health promotion, disease prevention and management of NCDs and monitoring progress towards reducing mortality due to NCD’s. |
| **Evidence of implementation** | **Healthy Ireland**  **Health Service Executive – Health and Wellbeing Division**  The Health and Wellbeing division of the HSE is focused on helping people to stay healthy and well, reducing health inequalities and protecting people from threats to their health and wellbeing. It was established based on two fundamental policy shifts within the health service – Future Health, which describes the new structures currently being established for the healthcare system, and Healthy Ireland; the government framework to improve the health and wellbeing of our population.  **Healthy Ireland, A Framework for Improved Health and Wellbeing 2013- 2025** Healthy Ireland is a government-led initiative, which aims to create an Irish society where everyone can enjoy physical and mental health, and where wellbeing is valued and supported at every level of society. |

DOMAIN 13 – Platforms for Interaction

**There are coordination platforms and opportunities for synergies across government departments, levels of government and other sectors (e.g. National Government Organisations, private sector, academia) such that policies and actions in physical activity are coherent, efficient and effective in improving environments, population physical activity, reducing inactivity related non-communicable diseases and their related inequities.**

| **PI01**  **There are robust coordination mechanisms across departments and levels of government to ensure policy coherence, alignment and integration of physical activity, and inactivity related non-communicable disease prevention policies across governments.** | |
| --- | --- |
| **Definitions and scope** | - Includes cross-government or cross-departmental governance structures, committees or working groups (at multiple levels of seniority), agreements, memoranda of understanding, etc. - Includes cross-government or cross-departmental shared priorities, targets or objectives - Includes strategic plans or frameworks that map the integration and alignment of multiple policies or programs across governments and across departments - Includes cross-government or cross-departmental collaborative planning, implementation or reporting processes, consultation processes for the development of new policy or review of existing policy (Food EPI Evidence Document – PLAT1). |
| **International best practice examples (benchmarks)** | **Sweden**  The Swedish Working Group on health-enhancing physical activity (SWHEPA) is the multisectoral coordinating body for promoting physical activity. It is led by the Public Health Agency of Sweden (folkhälsomyndigheten) and involves Government agencies and organizations at national level. (WHO Europe Factsheets verbatim)  **Malta**  Based on the Healthy Lifestyle Promotion and Care of NCDs Act (2016), Malta established an inter-ministerial Advisory Council on Healthy Lifestyles in August 2016 to advise the Minister of Health on any matter related to healthy lifestyles. In particular, the Advisory Council advises on a life course approach to physical activity and nutrition, and on policies, action plans and regulations intended to reduce the occurrence of NCDs. The prime minister appoints the chair and the secretary of the Advisory Council, while the ministers of education, health, finance, social policy, sports, local government, and home affairs appoint one member each (World Cancer Research Fund, 2016).  **Ireland**  The Health and Wellbeing Programme, tasked with planning and coordinating a national framework for improving health, is required to work with policy units within the various different government departments to produce integrated, intersectoral plans (Healthy Ireland Framework 2013, p19).  The Department of Health, through *‘A Healthy weight for Ireland, Obesity Policy and Action Plan 2016 – 2025’,* will provide leadership, engage and co-ordinate multi-sectorial action and implement best practice in the governance of the *OPAP*. The Department of Health and Safefood are taking action to establish a multi-stakeholder partnership to share knowledge and initiative to promote a healthy weight (Department of Health, 2016b). |
| **Context e.g. EU action / regulation** | **WHO**  **Global Action Plan on Physical Activity**  GAPPA Action 4.1: Strengthen policy frameworks, leadership and governance systems, at the national and subnational levels, to support implementation of actions aimed at increasing physical activity and reducing sedentary behaviours, including multisectoral engagement and coordination mechanisms; policy coherence across sectors; guidelines, recommendations and actions plans on physical activity and sedentary behaviour for all ages; and progress monitoring and evaluation to strengthen accountability.  Proposed sub action for member states 4.1.1: Initiate or strengthen, as appropriate, a high-level national multisectoral coordination committee to provide leadership, strategic planning and oversight of implementation and monitoring of national policy actions on physical activity and sedentary behaviour, ensuring appropriate representation from all relevant areas and levels of government, as well as nongovernmental stakeholders and the community.  Proposed sub action for member states 4.1.3: Partner with other sectors to review and, where needed, strengthen the position of physical activity within respective policy frameworks, including but not limited to community and grass roots sports within sports policy, walking and cycling within transport policy, physical education within education policy, and physical activity within integrated NCD and mental health policies (WHO, 2018).  **WHOROE**  **Physical activity strategy for the WHO European Region 2016–2025**  Objective 1.2: Establish coordination mechanisms and promote alliances. Member States should set up coordinating mechanisms among sectors, such as health, sports, education, transport, urban planning, environment and social affairs, and levels of government, for example, regional, national and local, with a view to identifying common objectives and mutual gains […](WHOROE, 2016).  **EU**  **2013 Council recommendations**  Indicator 4: National coordination mechanism on HEPA promotion (yes/no).  There are mechanisms at EU level, which aim to ensure co-operation and coordination at the level of the European Commission, the European Parliament, the Council, between the EU and the Member States, between Member States, and with subnational governments and social partners (Council of the European Union, 2013). |
| **Evidence of implementation** | **Ireland**  **Healthy Ireland, A Framework for Improved Health and Wellbeing 2013- 2025**  The Health and Wellbeing Programme, tasked with planning and coordinating a national framework for improving health, is required to work with policy units within the various different government departments to produce integrated, intersectoral plans (Healthy Ireland, 2013, p19).  **National Sport Policy 2018 – 2027**  Action 20 of NSP calls for inter-departmental collaboration in establishing a national swimming strategy: “We will work closely with relevant Government Departments, Local Authorities, sporting bodies and other stakeholders to agree a coherent national strategy for swimming. As part of this, we will review swimming pool provision to identify where gaps exist and how these can be met” (Department of Transport, Tourism and Sport, 2018; p46).  Action 39 states: “We will establish a Sports Leadership Group within three months of the publication of this policy to agree an Action Plan and to oversee its implementation once approved by Government” (see Leadership 01) (Department of Transport, Tourism and Sport, 2018; p83).  **National Sports Policy 2018 – 2027 Sports Action Plan 2021 – 2023**  In accordance with action 39 of NSP a Sports Action Plan was published. The Sports Action Plan lists the membership of the Sports Leadership Group (SLG) as: representatives from the Departments of Education (DoE); Health (DoH); Children, Equality, Disability, Integration and Youth (DCEDIY) and Tourism, Culture, Arts, Gaeltacht, Sport and Media (DTCAGSM), Ireland Active, CARA, Volunteer Ireland, the Olympic Federation of Ireland, the Olympic Federation of Ireland’s Athletes Commission, Sligo Local Sports Partnership, Paralympics Ireland, South Dublin County Council. the Federation of Irish Sport, Broadcasters and Sport Ireland (Department of Tourism, Culture, Arts, Gaeltacht, Sport and Media, 2021).  **A Healthy Weight for Ireland: Obesity Policy and Action Plan 2016 - 2025**  The Obesity Policy and Action Plan is a cross-sectoral and whole-of-government approach towards tackling the causes of obesity in Ireland. It is overseen by the Obesity Policy Implementation Oversight Group (OPIOG), which is comprised of representatives from a range of Government departments and agencies.    The Obesity Policy and Action Plan was developed under the Healthy Ireland Framework. The WHO selected the Healthy Ireland Framework as an example of good practice at the 69th session of the WHO Regional Committee for Europe, as it is one of the flagship whole-of-government approaches from across the European Region.    **Get Ireland Active! The National Physical Activity Plan for Ireland**  NPAP states, in action area 8 “implementation through partnership”, that an oversight and implementation group will be established to oversee implementation of the plan. The group, it is stated, will include a range of stakeholders led by the Department of Health and the Department of Transport, Tourism and Sport (Healthy Ireland, 2016). |

| **PI02**  **There are structures and mechanisms for regular, meaningful, and inclusive interactions between government and civil society (academia, professional organizations, public-interest, non-governmental organisations, and citizens) on physical activity policies and other strategies to improve population physical activity and health.** | |
| --- | --- |
| **Definitions and scope** | - Civil society includes community groups and consumer representatives, NGOs, academia, professional associations, etc. - Includes established groups, forums or committees active within the last 12 months for the purpose of information sharing, collaboration, seeking advice - Includes platforms for consultation on proposed plans, policy or public inquiries - Excludes policies or procedures that guide consultation in the development of physical activity policy (see G05) (Food EPI Evidence Document – PLAT3). |
| **International best practice examples (benchmarks)** | **Norway**  **The Action Plan on Physical Activity 2005-2009**  Chapter 8 of entitled “Together for physical activity” sets out several mechanisms by which local governments interact with civil society on PA. Actions within this chapter include action 94: which creates “meeting places” in the field of public health between public authorities, professional communities and voluntary organizations (Belander et al., 2011). |
| **Context e.g. EU action / regulation** | **WHOROE**  **Physical activity strategy for the WHO European Region 2016–2025**  Objective 1.2: Establish coordination mechanisms and promote alliances […]. Member States should promote alliances between government, the media, civil society organizations and other stakeholders, including, but not limited to, public health and sports organizations, in order to promote physical activity for health across the life-course […]”(WHOROE, 2016).  **EU**  **2013 Council recommendations**  Indicator 4: National coordination mechanism on HEPA promotion (yes/no) (Council of the European Union, 2013).  **European Health Policy Platform**  The European Health Policy Platform is a collaborative initiative under the 3rd Health Programme to ease communication among health stakeholders and with the European Commission (European Commission). DG SANTE acts as the secretary of the Platform.  **European Economic and Social Committee**  The European Economic and Social Committee (EESC) enables civil society organisations (350 members) from the Member States to express their views at European level (European Economic and Social Committee). Its opinions are addressed to the European Commission, Parliament and Council. Consultation of the EESC is mandatory for public health policies (Article 168 of the TFEU) (Official Journal of the European Union, 2012). The EESC may also adopt opinions on its own initiative (European Sources Online, 2013). |
| **Evidence of implementation** | **Ireland**  **Healthy Ireland Framework**  Under the Healthy Ireland Framework and through the Healthy Ireland Fund, the Healthy Counties and Cities approach is supporting existing cross-partnership groups that have been set up in each local authority.  **Get Ireland Active! National Physical Activity Plan for Ireland (implementation group)**  Implementation of NPAP is overseen by a cross sectoral NPAP Implementation group (action 56). Persons named as members of the group include representatives from the Department of Health, the former Department of Transport, Tourism and Sport, the Department of Children and Youth Affairs, the HSE, Sport Ireland, the Federation of Irish Sport, local government, and academia (Healthy Ireland, 2016).  Action 59 states that a programme of stakeholder communication, including an annual engagement forum will be established to ensure delivery of NPAP.  **National Sport Policy 2018 – 2027**  Action 34 of NSP states that “We will seek to foster and encourage collaboration within the sports sector and between sport and other sectors through training and development initiatives and through the provision of financial and other incentives to stimulate collaborative behaviour at all levels of sport. We will work on an all-island basis in this regard. We will seek to develop a stronger relationship with the education system in particular in our efforts.”  **Irish Physical Activity Research Collaboration**  Established in 2018, the Irish Physical Activity Research Collaboration (I-PARC) is a collaboration between physical activity researchers and knowledge users (policymakers and physical activity practitioners) (Murphy et al., 2021). The I-PARC core team includes representatives from academia, state agencies (from both the Republic and Northern Ireland) and the government departments with responsibility for health, education, transport and sport. I-PARC organises an annual conference bringing together various physical activity stakeholders. |

DOMAIN 14 – Workforce Development

**Governments have set up systems that provide a platform for population physical activity expertise to ensure that the formulation, implementation and evaluation of physical activity policies and programmes meet population needs.**

| **WD01**  **To address the challenge of population physical inactivity, there are sufficient resources and people with necessary skills within the government’s workforce (across all 8 policy domains).** | |
| --- | --- |
| **Definitions and scope** | - Includes professionals specialising in health promotion in the department with responsibility for health. - Includes physical education teachers - Includes physiotherapists |
| **International best practice examples (benchmarks)** | **England**  **Everybody active, every day**  In the development of the policy document *Everybody active, everyday framework for physical activity*, Public Health England recognised a need for professionals to ‘activate’ networks of public facing professionals to promote physical activity. They Identified the areas of ‘Education’, ‘sport and leisure’, ‘Health and social care’ and ‘Planning, design, development and transport’ (Public Health England, 2014).  To achieve their goal of activating professional networks Public Health England has in collaboration with academic partners developed teaching resources for training undergraduate healthcare professionals about physical activity. These have been adopted by over 20 Medical and Nursing schools across the UK  Public Health England has also launched ‘What works in schools and colleges to increase physical activity’ an initiative providing guidance to professionals working in education settings (Varney and Fenton, 2015; WHOROE, 2018). |
| **Context e.g. EU action / regulation** | **WHO**  **Global Action Plan on Physical Activity**  GAPPA Action 1.4: Strengthen pre- and in-service training of professionals, within and outside the health sector, to increase knowledge and skills related to their roles and contributions in creating inclusive, equitable, opportunities for an active society including but not limited to, the transport, urban planning, education, tourism and recreation, sports and fitness sectors as well as in grassroots community groups and civil society organizations.  Proposed sub action for member states 1.4.1: Strengthen the preservice and in-service curricula of all medical and allied health professionals to ensure effective integration of the health benefits of physical activity into the formal training on prevention and management of noncommunicable diseases, mental health, healthy ageing, child health and development, and wider promotion of community health and well-being (WHO, 2018). |
| **Evidence of implementation** | **Ireland**  **Healthy Ireland, A Framework for Improved Health and Wellbeing 2013- 2025** The Healthy Ireland Framework contains the following actions for developing the public health workforce:   - 4.3 Conduct a baseline assessment of strengths and weaknesses of current health and wellbeing workforce capacity. - 4.4 Develop a health, wellbeing human resource, and development plan with a view to building capacity for health and wellbeing activities.   **Physiotherapists (Health) - Physiotherapists Registration Board** The titles of “physiotherapist” and “physical therapist” are protected for the exclusive use of people registered with the Physiotherapists Registration Board - one of several ‘competent authorities’ established to regulate health and social care professions. In 2015, immediately prior to the establishment of the registry, the number of practicing physiotherapists in the country was estimated at 3172 or 6.8 per 10 000 (Eighan et al. 2018). The registry of the board was opened in 2016. The number of physiotherapists registered with the board was reported by the board as being 1782 in 2018, 3563 in 2019 and 4651 in 2020 (Health and Social Care Professionals Council, 2018; 2019; 2020).  **Physical Education Teachers (Education) - Teaching Council of Ireland** There are five higher education institutions that provide initial training for primary school teachers in Ireland: Maynooth University, Mary Immaculate College Limerick, and Dublin City University, Marino Institute of Education, and Hibernia College in Dublin (Teaching Council of Ireland, n.d.). There are 14 training post primary teachers ([Dublin City University,](https://www.dcu.ie/) [Galway–Mayo Institute of Technology](http://www.gmit.ie/), [Hibernia College](http://www.hiberniacollege.com/), [Limerick Institute of Technology (Art and Design)](http://www.lit.ie/), [Maynooth Universit](https://www.maynoothuniversity.ie/)y, Mary Immaculate College, [National College of Art & Design](http://www.ncad.ie/), [National University of Ireland Galway](http://www.nuigalway.ie/), [St Angela’s College](http://www.stangelas.nuigalway.ie/), [Trinity College Dublin](http://www.tcd.ie/), [University](https://www.ucc.ie/en/) College Cork, [University](https://www.ucc.ie/en/) College Dublin and [University of Limerick](http://www.ul.ie/)).  Teachers, including physical education teachers, must be recognised in the register maintained by the Teaching Council of Ireland. Teaching Council of Ireland is the statutory body responsible for regulating the teaching profession in Ireland.  Physical Education is taught by specialist physical education teachers at the post primary level (see Good Practice Statement Education 01). In 2020, A total of 105 933 teachers were registered in 2020. The number of teachers who were registered as post primary level teachers was 45 410. (Teaching Council of Ireland, 2020a). The register can be searched at: <https://my-tc.teachingcouncil.ie/Find-a-Teacher>.  **Inspectorate of the Department of Education (Education)**  The Inspectorate evaluates primary schools, post primary schools and centres for education. This mechanism provides an assurance of quality and accountability, including for physical education teachers (Department of Education, 2021).  **The Professional Development Service for Teachers**  The PDST develops support and professional learning opportunities to teachers, including physical education teachers.  In 2012, The PDST, in collaboration with the Department of Education and Skills and other government departments, published the document “Get Active! Physical Education, Physical Activity and Sport for Children and Young People: A Guiding Framework” as a support for people working to promote children’s physical activity (Department of Education and Skills, 2012).  **National Sport Policy 2018 – 2027**  Chapter seven of NSP is titled ‘Building Capacity’. Action 28 states that a fund to train volunteers across sports will be established. The training will focus on “child welfare, disability awareness, first aid, sports administration and governance, and fundraising” (DTTS, 2018, p67). Action 27 states that Sport Ireland will develop a volunteering strategy that will promote recruitment and retention. Action states that a coaching plan, seeking to increase diversity in the coaching workforce and to making coaching qualifications more adaptable, will be developed. |

| **WD02**  **Opportunities for training and professional development are provided to relevant individuals across multiple sectors (e.g., the 8 'Policy' domains) regarding the fundamentals of physical activity, its role in public health, and effective strategies for physical activity promotion.** | |
| --- | --- |
| **Definitions and scope** | - ‘Relevant individuals’ regards intermediaries who professionally work with the workforce with a focus on getting/keeping them healthy. These include professionals specialised in health promotion in the department with responsibility for health, physical education teachers, physiotherapists, etc. |
| **International best practice examples (benchmarks)** | **England**  “Moving health care professionals” is a multi-component, partnership-based programme to increase the awareness and skills of health professionals and to change their clinical practice in promoting physical activity to patients at high risk of or with health conditions. The programme provided physical activity sessions to over 20 000 health care professionals, and its e-learning modules have been completed over 10 000 times by these professionals.  **Czechia**  The project “Short intervention web” in Czechia is supported by the National Health Programme of the Ministry of Health. It provides for short interventions by professionals who are trained by the National Institute of Health in the “short intervention method”, which teaches communication and motivation. The method is suitable for workers in health care, social services and education who are interested in educating their patients, clients or pupils about risk factors and health-enhancing lifestyle behaviour, including physical activity, diet, smoking and alcohol consumption.  Between 2013 and 2015, multiple training seminars were provided free of charge in a pilot programme. In 2016 and 2017, the project was implemented in 11 health facilities and selected higher education institutions. The project continues in 2018 and now provides materials free of charge on its website. |
| **Context e.g. EU action / regulation** | **Global Action Plan on Physical Activity**  Proposed sub action for member states 1.4.2: Partner with the education sector to strengthen formal preservice and in-service training for preschool, primary and secondary school teaching staff and administrators to strengthen knowledge and teaching skills on the value of active play, physical education, adaptive physical activity, fundamental movement skills and physical literacy, and on how to include people with disabilities and the least active.  Proposed sub action for member states 1.4.3: Partner to secure the inclusion of physical activity in the professional education of relevant sectors outside of health to understand the value of promoting physical activity, including, but not limited to, transport, urban planning, education, social care, tourism, recreation, and sports and fitness.  Proposed sub action for member states 1.4.4: Partner with road safety experts to strengthen stakeholders’ understanding of safe systems approaches to improving road safety for pedestrians, cyclists and public transport users, in alignment with The Decade of Road Safety (WHO, 2018).  **EU**  **2013 Council recommendations**  Indicator 12: Training on physical activity in curriculum for health professionals (number of hours for nurses, doctors / mandatory or optional / clear assessment and accreditation structures to reflect the learning outcomes of the subject)  Indicator 15: HEPA in training of physical education teachers. HEPA being a module in training of PE teachers at bachelor's and/or master's degree level (Yes / no ; Mandatory / optional) (Council of the European Union, 2013). |
| **Evidence of implementation** | **Ireland**  **Get Ireland Active! National Physical Activity Plan for Ireland**  Action 13 of the National Physical Activity Plan (NPAP) states that Healthy Ireland will "Develop and provide a programme of continuous professional development [(CPD)] in physical education" (Healthy Ireland, 2016 p13). Action 22 states that training on physical activity will be incorporated into in-service training of all health professionals (Healthy Ireland, 2016). Action 35 commits to a programme of CPD on physical activity for persons working in "developing the built environment" (Healthy Ireland, 2016 p38).  Implementation reports cite supports provided by the Professional Development Service for Teachers (PDST, see below) as implementation of action 13, and the brief intervention model, Making Every Contact Count (MECC, see Mass Media 01), as evidence of activity on action 22. It states that CPD for planners is already on-going and provided by professional bodies (Healthy Ireland et al., 2021).    **National Sport Policy 2018 - 2027**  The National Sports Policy (NSP) states, in action 33, that "Sport Ireland will lead the development of a sports sector workforce strategy which includes provision of CPD" (Department of Transport, Tourism and Sport, 2018 p102).    **Physiotherapists**  CPD is a professional requirement for physiotherapists (Register of Exercise Professionals, 2020). According to article 10.1 of the code of professional conduct and ethics, physiotherapists must:   - Ensure that their knowledge, skills and performance are of a high standard, up to date and relevant to their practice. - Participate in CPD on an ongoing basis.   Furthermore, physiotherapists are advised to;   - Consider the support and guidance provided by their Registration Board regarding CPD. - Keep a record of the activities they have completed (Physiotherapists Registration Board, 2019).     **Exercise professionals – REPS Ireland**  The Register of Exercise Professionals in Ireland (REPs Ireland) maintains standards in the fitness industry. REPS Ireland maintains a list of recognised CPD providers. A recent requirement is that registered exercise professionals must earn 20 CPD ‘points’ every 2 years. (REPS Ireland, n.d.). |

| **WD03**  **Support and training systems are in place for relevant professionals (e.g., guidelines, toolkits, training workshops/modules/courses). To ensure uptake, accrediting agencies for professional education, and professional licensing entities should include minimum requirements for initial and continuing education in this domain.** | |
| --- | --- |
| **Definitions and scope** | - Monitoring/ensuring education through standardisation of minimum requirements through an accrediting system |
| **International best practice examples (benchmarks)** | **France**  **BP JEPS diploma**  The professional diploma in youth, popular education and sport (Brevet professionnel de la jeunesse, de l'éducation populaire et du sport or BP JEPS) provides holders with the qualifications to supervise physical activities. The diploma is registered in the national directory of professional certifications (Répertoire national des certifications professionnelles or RNCP). |
| **Context e.g. EU action / regulation** | **EU**  **Treaty on the Functioning of the European Union (TFEU)**  The Treaty of Rome was promulgated in 1957. The Treaty was amended by the Nice (2001) and Lisbon (2007) Treaties. On both occasions the name of the Treaty was changed (to “Treaty Establishing the European Community” or TEC and to “Treaty on the Functioning of the European Union” or TFEU respectively).  Article 40 of the treaty states that the relevant European Institutions shall “in accordance with the ordinary legislative procedure [...], issue directives or make regulations setting out the measures required to bring about freedom of movement for workers”. These measures include “ensuring close cooperation between national employment services” and “abolishing those administrative procedures and practices [...], the maintenance of which would form an obstacle to liberalisation of the movement of workers  Article 47 (1) of the treaty states that the institutions shall “issue directives for the mutual recognition of diplomas, certificates and other evidence of formal qualifications”.  Article 47 (2) of the treaty states that the institutions states “In the case of the medical and allied and pharmaceutical professions, the progressive abolition of restrictions shall be dependent upon coordination of the conditions for their exercise in the various Member States”.  Due to the amendments to the Treaty mentioned above, Article 40 of the Treaty appears as Article 46 in the consolidated version of the Treaty and Article 47 of the Treaty appears as Article 53 in the consolidated version of the Treaty.  **Directive 2005/36/EC**  Directive 2005/36/EC “on the recognition of professional qualifications” actions the treaty articles mentioned above. |
| **Evidence of implementation** | **Ireland**  **Physiotherapists Training - Physiotherapist Registration Board**  Through its bye laws, the Physiotherapists Registration Board recognises a short list of third level qualifications as evidence of the standard of proficiency required for registration in the register of physiotherapists (Physiotherapists Registration Board Approved Qualifications Bye-Law, 2020). Professionals with qualifications awarded outside the Republic of Ireland may apply for recognition by the board.    **Physical Education Teacher Training - Teaching Council of Ireland**  There are two main routes whereby a person can become qualified as a teacher in Ireland according to the Teaching Council. The "consecutive" route requires an undergraduate degree which enables the holder to teach at least one approved curricular subject and a Professional Masters of Education (PME). The "concurrent" route requires a degree which combines the study of approved curricular subjects with school placement and other professional studies (Citizens Information, 2018; The Teaching Council of Ireland 2020). Physical Education is a curriculum subject that one can specialise in at various institutions in the Republic. The teaching council specifies content that must be included in the degree to enable the holder to teach physical education. Teachers with qualifications from outside the Republic of Ireland may apply for recognition by the Teaching Council (The Teaching Council of Ireland, 2020b).    **Coaches – Sport Ireland Coaching Development Programme**  Sport Ireland works with the National Governing Bodies (NGBs) of sport and the wider sporting industry to operate the Coaching Development Programme for Ireland. There are two framework outlining coaching competencies: “Competition Coach Framework” and the “Adventure Sport Instructor Framework”. The NGBs develop accreditation for coaches within the parameters set out in these syllabuses (Sport Ireland, n.d.).  **Exercise professionals – REPS Ireland**  The Register of Exercise Professionals in Ireland (REPs Ireland) maintains standards in the fitness industry. REPS Ireland has an auditing process for recognising providers of quality fitness education and graduates of recognised courses are eligible to register with REPS Ireland. A list of approved education providers is available on the REPs Ireland website (available at: https://repsireland.ie/approved-education-providers/). This list includes courses provided by fitness industry academies and third level institutions in Ireland. Governance of REPS Ireland is undertaken by the REPs Ireland council established by Ireland Active, a non-profit trade body (Ireland Active, n.d.). The fitness industry in Ireland is therefore a self-regulating industry. |

DOMAIN 15 – Health-in-All Policies

**There are processes in place to ensure policy coherence and alignment, and that population health impacts are explicitly considered in the development of all relevant government policies.**

| **HIAP01**  **There are processes in place to ensure that population physical activity and related health outcomes are explicitly and transparently considered and prioritised in the development of all government policies.** | |
| --- | --- |
| **Definitions and scope** | - Includes policies, procedures, guidelines, tools and other resources that guide the consideration and assessment of physical activity, health outcomes and reducing health inequalities or health impacts in vulnerable populations prior to, during and following implementation of physical activity-related policies. - Includes the establishment of cross-department governance and coordination structures while developing physical activity-related policies. |
| **International best practice examples (benchmarks)** | No suitable examples identified. |
| **Context e.g. EU action / regulation** | **WHO**  **Global Action Plan on Physical Activity**  GAPPA Action 4.1: Strengthen policy frameworks, leadership and governance systems, at the national and subnational levels, to support implementation of actions aimed at increasing physical activity and reducing sedentary behaviours, including multisectoral engagement and coordination mechanisms; policy coherence across sectors; guidelines, recommendations and actions plans on physical activity and sedentary behaviour for all ages; and progress monitoring and evaluation to strengthen accountability.  Proposed sub action for member states 4.1.1: Initiate or strengthen, as appropriate, a high-level national multisectoral coordination committee to provide leadership, strategic planning and oversight of implementation and monitoring of national policy actions on physical activity and sedentary behaviour, ensuring appropriate representation from all relevant areas and levels of government, as well as nongovernmental stakeholders and the community.  Proposed sub action for member states 4.1.3: Partner with other sectors to review and, where needed, strengthen the position of physical activity within respective policy frameworks, including but not limited to community and grass roots sports within sports policy, walking and cycling within transport policy, physical education within education policy, and physical activity within integrated NCD and mental health policies (WHO, 2018).  **EU**  **2013 Council recommendations**  Indicator 22: National HEPA policies that include a plan for evaluation (Council of the European Union, 2013). |
| **Evidence of implementation** | **Ireland**  **Healthy Ireland, A Framework for Improved Health and Wellbeing 2013- 2025**  The Taoiseach's foreword reflecting the desires of the government of the day for Healthy Ireland. It states: "we want to make sure that all sectors appreciate that they have a role to play and that health and wellbeing is affected by a whole range of factors" (Healthy Ireland, 2013 p.1). The statement makes clear that Healthy Ireland is intended to include "all sectors".    **Health Service Executive - Health Promotion Strategic Framework**  The Health Service Executive (HSE) Health Promotion Strategic Framework (2012) places the emphasis for health promotion activity on addressing the determinants of health and health inequalities, which will primarily be achieved through strong national leadership aimed at putting health on the agenda of all those involved in planning and decision-making. This includes advocating for a Health in All Policies approach (HiAP) as well as building and strengthening cross-sectoral and inter-departmental government partnerships (Health Service Executive, 2012). |

| **HIAP02**  **There are processes (e.g., health impact assessments) to assess and consider health impacts during the development of policies indirectly related to physical activity.** | |
| --- | --- |
| **Definitions and scope** | - Includes a government wide HiAP strategy or plan with clear actions for non-health sectors. - Includes policies, guidelines, tools and other resources that guide the consideration and assessment of health impacts prior to, during and following implementation of non-physical activity related policies (e.g., HIAs or health lens analysis). - Includes the establishment of cross-department or cross-sector governance and coordination structures to implement a HiAP approach - Includes monitoring or reporting requirements related to health impacts for non-health departments. |
| **International best practice examples (benchmarks)** | **South Australia**  Established in 2007, the successful implementation of Health in All Policies (HiAP) in South Australia has been supported by a high-level mandate from central government, an overarching framework which is supportive of a diverse program of work, a commitment to work collaboratively and in partnership across agencies, and a strong evaluation process. The government has established a dedicated HiAP team within South Australia Health to build workforce capacity and support Health Lens Analysis projects. Since 2007, the South Australian HiAP approach has evolved to remain relevant in a changing context. However, the purpose and core principles of the approach remain unchanged. There have been five phases to the work of HiAP in South Australia between 2007 and 2016: 1) Prove concept and practice emerges (2007-2008), 2) Establish and apply methodology (2008-2009), 3) Consolidate and grow (2009-2013), 4) Adapt and review (2014) and 5) Strengthen and systematize (2015-2016).  **Utrecht, Netherlands**  The Dutch city of Utrecht has implemented a policy of performing health impact assessments for all its initiatives and works. |
| **Context e.g. EU action / regulation** | **Global Action Plan on Physical Activity**  GAPPA Action 2.1: Strengthen the integration of urban and transport planning policies that prioritize the principles of compact, mixed land use, at all levels of government as appropriate, to deliver highly connected neighbourhoods that enable and promote walking, cycling, other forms of mobility involving the use of wheels (including wheelchairs, scooters and skates) and the use of public transport, in urban, peri-urban and rural communities.  Proposed sub action for member states 2.1.2: Support implementation of comprehensive health and economic assessments of transport and urban planning policies and interventions to assess their impact on health and physical activity as well their environmental impacts (such as air and noise pollution, carbon emissions) to inform decisions, which are consistent with a health in all policies approach. Use of the WHO HEAT tool (3) is recommended to support economic assessment of investment in walking and cycling networks and new infrastructure (WHO, 2018). |
| **Evidence of implementation** | **Healthy Ireland Framework 2013-2025**  **Healthy Ireland, A Framework for Improved Health and Wellbeing 2013- 2025**  The national framework to improve health and wellbeing 'Healthy Ireland' acknowledges that inter-sectoral working, or Health in All Policies, is a politically challenging strategy that requires deliberate efforts to be promoted. The inter-sectoral approach requires the use of validated tools and support mechanisms to drive this agenda, including for example, Health Impact Assessment (HIA), inter-ministerial and inter-departmental committees, cross-sector action teams, joined-up workforce development, and legislative frameworks.    In Ireland, the government has committed to incorporating poverty impact assessment as part of an integrated social impact assessment. Health impacts will be a core feature of this new tool. The Healthy Ireland Outcomes Framework aims to provide a structured approach to collect and report relevant and appropriate data, which can be used to build awareness of these social determinants of health, to support assessment of the impact of policies on the agreed outcomes, and to monitor progress on the whole-of-government response needed to improve health and wellbeing (Healthy Ireland, 2013).    **Institute of Public Health in Ireland**  The Institute of Public Health (IPH) received funds from the Governments of the Republic of Ireland and Northern Ireland. The IPH conducts training and advocacy on the use of Health Impacts Assessments (HIAs) and documents providing information and guidance on utilising HIAs are available on the Institute’s website (IPH, n.d.). IPH has conducted HIAs of public policies at the behest of the Department of Health (IPH, 2013). |

# **7. References**

Active School Flag (2021) About ASF [online], available: https://activeschoolflag.ie/about-asf/ [accessed 20 Aug 2021].

Active Together (2021) Workplace Challenge - Competition Programme [online], available: https://www.active-together.org/campaigns/workplace-challenge---competition-programme [accessed 16 Aug 2021].

Agbo, S., Gbaguidi, L., Biliyar, C., Sylla, S., Fahnbulleh, M., Dogba, J., Keita, S., Kamara, S., Jambai, A., Harris, A., Nyenswah, T., Seni, M., Bhoye, S., Duale, S., Kitua, A. (2019) ‘Establishing National Multisectoral Coordination and collaboration mechanisms to prevent, detect, and respond to public health threats in Guinea, Liberia, and Sierra Leone 2016–2018’, One Health Outlook, 1(1), 1–13.

Age & Opportunity (2022) CarePALs Physical Activity Leaders in Care Settings [online] available: <https://ageandopportunity.ie/active/carepals/> [Accessed 20 Feb 2022].

Agence Nationale de Sécurité Sanitaire de l’Alimentation (2020) Keep up Physical Activity and Limit Sedentary Behaviour: ANSES Adapts Its Guidelines to the Lockdown Situation [online], available: https://www.anses.fr/en/content/keep-physical-activity-and-limit-sedentary-behaviour-anses-adapts-its-guidelines-lockdown [accessed 1 Jan 2022].

An Taisce (2021) Travel [online], Green-Schools, available: https://greenschoolsireland.org/themes/travel/ [accessed 18 Aug 2021].

Anderson, J.O., Mullins, R.M., Siahpush, M., Spittal, M.J., Wakefield, M. (2009) ‘Mass media campaign improves cervical screening across all socio-economic groups’, Health Education Research, 24(5), 867–875.

Ashworth, G.J. (2001) ‘Planning by referendum: Empowerment or anarchy in Groningen, the Netherlands’, Local Environment, 6(3), 367–372.

Aubert, S., Barnes, J.D., Abdeta, C., Nader, P.A., Adeniyi, A.F., Aguilar-Farias, N., Tenesaca, D.S.A., Bhawra, J., Brazo-Sayavera, J., Cardon, G., Chang, C.K., Delisle Nyström, C., Demetriou, Y., Draper, C.E., Edwards, L., Emeljanovas, A., Gába, A., Galaviz, K.I., González, S.A., Herrera-Cuenca, M., Huang, W.Y., Ibrahim, I.A.E., Jürimäe, J., Kämppi, K., Katapally, T.R., Katewongsa, P., Katzmarzyk, P.T., Khan, A., Korcz, A., Kim, Y.S., Lambert, E., Lee, E.Y., Löf, M., Loney, T., López-Taylor, J., Liu, Y., Makaza, D., Manyanga, T., Mileva, B., Morrison, S.A., Mota, J., Nyawornota, V.K., Ocansey, R., Reilly, J.J., Roman-Viñas, B., Silva, D.A.S., Saonuam, P., Scriven, J., Seghers, J., Schranz, N., Skovgaard, T., Smith, M., Standage, M., Starc, G., Stratton, G., Subedi, N., Takken, T., Tammelin, T., Tanaka, C., Thivel, D., Tladi, D., Tyler, R., Uddin, R., Williams, A., Wong, S.H.S., Wu, C.L., Zembura, P., Tremblay, M.S. (2018) ‘Global Matrix 3.0 physical activity Report Card grades for children and youth: Results and analysis from 49 countries’, Journal of Physical Activity and Health, 15(Suppl 2), S251–S273.

Auckland Council (2018) Auckland Plan 2050 Overview, Auckland, New Zealand.

Australian Public Service Commission (2010) ‘Management Advisory Committee’, available: <http://www.directory.gov.au/directory?ea0_lf99_120.&organizationalUnit&b34767c6-3431-4d12-b56a-480e8660c912>.

Belander, O., Sæthre, E., Sjettne, S., Grendstad, G., Haug, G., Løvås, S.M., Andenæs, S. (2011) Health-Enhancing Physical Activity (HEPA) Policy Audit Tool (PAT) - Norway, available: https://www.panh.ch/hepaeurope/materials/hepa_pat_files/HEPA_PAT_FINAL_Norway.pdf.

Bergsgard, N.A., Borodulin, K., Fahlen, J., Høyer-Kruse, J., Iversen, E.B. (2019) ‘National structures for building and managing sport facilities: a comparative analysis of the Nordic countries’, Sport in Society, 22(4), 525–539, available: <https://doi.org/10.1080/17430437.2017.1389023>.

Belton, S., Britton, Ú., Murtagh, E., Meegan, S., Duff, C. and McGann, J., 2020. Ten Years of ‘Flying the Flag’: An Overview and Retrospective Consideration of the Active School Flag Physical Activity Initiative for Children—Design, Development & Evaluation. *Children*, *7*(12), p.300.

Blom, A., Inkinen, V., Kari, J., Laine, K., Moilanen, N., Paajanen, M., Rajala, K., Tammelin, T. (2016) Towards More Active and Pleasant School Days Interim Report on the Finnish Schools on the Move Programme 2015-2016, Liikkuva koulu, Helsinki, Finland, available: https://liikkuvakoulu.fi/sites/default/files/lk_valiraportti_111017_en.pdf.

Bloomberg, M., Burney, D., Farley, T., Sadik-Khan, J., Burden, A. (2010) Active Design Guidelines: Promoting Physical Activity and Health in Design, New York, USA.

Bloomberg Philanthropies Support LLC (2021) Partnership for Healthy Cities [online], available: https://partnershipforhealthycities.bloomberg.org/cities/ [accessed 11 Aug 2021].

Broderick, J., Moran, J., McCusker, J., Hartnett, B., Dillon, M., Lawless, A., Brosnan, A., Molloy, L., McDonough, C., Mannion, N., Kirwan, C., Sweeney, M., and Griffin, D. (2018) Let's Get Active!... to Improve Health and Wellbeing [online] available: <https://www.hse.ie/eng/about/who/healthwellbeing/our-priority-programmes/heal/heal-docs/lets-get-active-guidelines.pdf> [Accessed 20 Feb 2022].

Bronfenbrenner, U. (1979) The Ecology of Human Development: Experiments by Nature and Design, Harvard university press.

Burstein, P. (1991) ‘Policy Domains: Organization, Culture, and Policy Outcomes’, Annual Review of Sociology, 17, 327–350.

Cambridge English Dictionary (2021) Government Intervention | Meaning in the Cambridge English Dictionary [online], available: https://dictionary.cambridge.org/dictionary/english/government-intervention [accessed 22 Jul 2021].

Central Statistics Office (2016) 8. Travel Patterns and Car Ownership, Cork, Ireland, available: <https://www.cso.ie/en/media/csoie/newsevents/documents/census2016summaryresultspart2/Chapter_8_Travel_patterns_and_car_ownership.pdf>.[Accessed 10 Feb 2022]

Central Statistics Office (2022) Census of Population of Ireland [online] available: <https://www.census.ie/app/uploads/2021/10/Sample-Census-2022-Household-Form-English.pdf> [Accessed 10 Feb 2022]

Centers for Disease Control and Prevention Department for Policy and Strategy, 2013. Policy Evaluation [WWW Document]. URL https://www.cdc.gov/policy/analysis/process/evaluation.html (accessed 10.2.20).

Centers for Disease Control and Prevention (2013) Brief 5: Evaluating Policy Impact, available: http://www.wkkf.org/knowledge- [accessed 12 Jan 2022].

Chastin, S., Abaraogu, U., Bourgois, J., Dall, P., Darnborough, J., Duncan, E., Dumortier, J., Jiménez Pavón, D., McParland, J., Roberts, N. (2020) ‘Physical Activity, Immune Function and Risk of Community Acquired Infectious Disease in the General Population: Systematic Review and Meta-Analysis’, Immune Function and Risk of Community Acquired Infectious Disease in the General Population: Systematic Review and Meta-Analysis.

Christensen, T., Lægreid, P. (2007) ‘The whole-of-government approach to public sector reform’, Public Administration Review, 67(6), 1059–1066.

Citizens Information (2018) Teacher Qualifications [online], available: https://www.citizensinformation.ie/en/education/primary_and_post_primary_education/teachers_and_schools/teacher_qualifications_at_primary_and_post_primary_level.html [accessed 8 Jan 2022].

Citizens Information (2021a) Local government [online] available: <https://www.citizensinformation.ie/en/government_in_ireland/local_and_regional_government/functions_of_local_authorities.html> [Accessed 22 Mar 2022].

Citizens Information (2021b) Bike to Work Scheme [online], available: https://www.citizensinformation.ie/en/travel_and_recreation/cycling/cycle_to_work_scheme.html [accessed 20 Jan 2022].

Coillte (2017) Outdoor Recreation Plan for Public Lands and Waters in Ireland 2017 – 2021 [online], available: <https://www.coillte.ie/media/2017/06/ORP_Screen.pdf> [accessed 10 Feb 2022].

Collins English dictionary (2021a) Action Plan Definition and Meaning | Collins English Dictionary [online], available: https://www.collinsdictionary.com/dictionary/english/action-plan [accessed 21 Jul 2021].

Collins English dictionary (2021b) Disadvantage Definition and Meaning | Collins English Dictionary [online], HarperCollins Publishers, available: https://www.collinsdictionary.com/dictionary/english/disadvantagesch [accessed 16 Jul 2021].

Collins English dictionary (2021c) Effective Definition and Meaning | Collins English Dictionary [online], HarperCollins Publishers, available: https://www.collinsdictionary.com/dictionary/english/effective [accessed 30 Sep 2021].

Collins English dictionary (2021d) NGO Definition and Meaning | Collins English Dictionary [online], HarperCollins Publishers, available: https://www.collinsdictionary.com/dictionary/english/ngo [accessed 30 Sep 2021].

Collins English dictionary (2021e) Schemes Definition and Meaning | Collins English Dictionary [online], HarperCollins Publishers, available: https://www.collinsdictionary.com/dictionary/englishtoo/scheme [accessed 30 Sep 2021].

Collins English dictionary (2021f) Schools Definition and Meaning | Collins English Dictionary [online], HarperCollins Publishers, available: https://www.collinsdictionary.com/dictionary/english/school [accessed 30 Sep 2021].

Collins English dictionary (2021g) Synergies Definition and Meaning | Collins English Dictionary [online], HarperCollins Publishers, available: https://www.collinsdictionary.com/dictionary/english/synergies [accessed 30 Sep 2021].

Collins English dictionary (2021h) Tools Definition and Meaning | Collins English Dictionary [online], HarperCollins Publishers, available: https://www.collinsdictionary.com/dictionary/english/tool [accessed 30 Sep 2021].

Collins English dictionary (2021i) Walkable Definition and Meaning | Collins English Dictionary [online], HarperCollins Publishers, available: https://www.collinsdictionary.com/dictionary/english/walkable [accessed 30 Sep 2021].

Cochran, C.L., Malone, E.F. (2014) Public Policy: Perspectives and Choices, 3rd ed, International Encyclopedia of Human Geography, Lynne Rienner: Boulder, Colorado

Cork Sports Partnership (2018) Sports Inclusion Disability Charter [online], available: https://www.corksports.ie/sports-inclusion-disability-charter/ [accessed 12 Jan 2022].

Coulter, M., Scanlon, D., MacPhail, A., O’Brien, W., Belton, S., Woods, C. (2020) ‘The (mis)alignment between young people’s collective physical activity experience and physical education curriculum development in Ireland’, Curriculum Studies in Health and Physical Education, 11(3), 204–221, available: https://doi.org/10.1080/25742981.2020.1808493.

Council of Europe (1976) ‘European Sport for All Charter’, in Resolution (76) 41 of the Committee of Ministers, Principles for a Policy for Sport for All, Strasburg, 1–3.

Council of the European Union (2013) Council Recommendation of 26 November 2013 on Promoting Health-Enhancing Physical Activity across Sectors (2013/C 354/01) [online], Official Journal of the European Union, available: <https://eur-lex.europa.eu/legal-content/GA/TXT/?uri=celex%3A32013H1204%2801%29>.

Cunningham, C., O’Sullivan, R. (2021) ‘Healthcare Professionals Promotion of Physical Activity with Older Adults: A Survey of Knowledge and Routine Practice’, *International Journal of Environmental Research and Public Health*, 18(11), 6064.

Damschroder, L.J., Aron, D.C., Keith, R.E., Kirsh, S.R., Alexander, J.A., Lowery, J.C. (2009) ‘Fostering implementation of health services research findings into practice: A consolidated framework for advancing implementation science’, Implementation Science, 4(1), 1–15.

De Geest, L. (2017) Gent’s Traffic Circulation Plan (Belgium) [online], Eltis, available: https://www.eltis.org/discover/case-studies/gents-traffic-circulation-plan-belgium [accessed 11 Aug 2021].

Department of An Taoiseach (2020a) Programme for Government: Our Shared Future [online] available: [[[https://www.gov.ie/en/publication/7e05d-programme-for-government-our-shared-future/https://www.gov.ie/en/publication/7e05d-programme-for-government-our-shared-future/](https://www.gov.ie/en/publication/7e05d-programme-for-government-our-shared-future/)](https://www.gov.ie/en/publication/7e05d-programme-for-government-our-shared-future/)](https://www.gov.ie/en/publication/7e05d-programme-for-government-our-shared-future/) [Accessed 22 Mar 2022].

Department of an Taoiseach (2020b) Taoiseach launches #InThisTogether campaign for people to Stay Connected, Stay Active and Stay Mentally Well during COVID-19. [online], available: <https://www.gov.ie/en/press-release/433d0f-an-taoiseach-launches-inthistogether-campaign-for-people-to-stay-con/> [accessed 09 Feb 2022].

Department of an Taoiseach and Department of Health (2020) Resilience and Recovery 2020-2021 Plan for Living with COVID-19. [online], available: <https://www.gov.ie/en/publication/e5175-resilience-and-recovery-2020-2021-plan-for-living-with-covid-19/> [accessed 09 Feb 2022].

Department of Children, Equality, Disability, Integration and Youth (2017) Better Outcomes, Brighter Futures: The National Policy Framework for Children and Young People, 2014-2020 [online], available: <https://www.gov.ie/en/publication/775847-better-outcomes-brighter-futures/> [accessed 10 Feb 2022].

Department of Communications, Climate Action and Environment (2019) Climate Action Plan 2019 To Tackle Climate Breakdown. Dublin, Ireland. Available: <https://assets.gov.ie/25419/c97cdecddf8c49ab976e773d4e11e515.pdf> [Accessed 04 Apr 2022].

Department of Communications, Climate Action and Environment (2021) Climate Action Plan 2021 Securing Our Future. Dublin, Ireland. Available: <https://www.gov.ie/en/publication/6223e-climate-action-plan-2021/> [Accessed 04 Apr 2022].

Department of Children, Equality, Disability, Integration and Youth (2017) Better Outcomes, Brighter Futures: The National Policy Framework for Children and Young People, 2014-2020 [online], available: <https://www.gov.ie/en/publication/775847-better-outcomes-brighter-futures/> [Accessed 10 Feb 2022].

Department of Education and Science (1999) Physical Education Teacher Guidelines, Public Health, Dublin, Ireland. Available: <https://curriculumonline.ie/getmedia/2ca06265-2e75-4cc1-8174-661a877728d4/PE_Guidelines_english.pdf> [Accessed 03 Apr 2022].

Department of Education and Skills (2012) Get Active! Physical Education, Physical Activity and Sport for Children and Young People. A Guiding Framework. Dublin, Ireland. [online], Available: https://www.gov.ie/en/publication/d53e68-get-active-physical-education-physical-activity-and-sport-for-childr/. [Accessed 08 Feb 2022]

Department of Education and Skills (2017a) Physical Education Curriculum Specification. Available: <https://www.curriculumonline.ie/Senior-cycle/Senior-Cycle-Subjects/Physical-Education-Specification/> [Accessed 03 Apr 2022].

Department of Education and Skills (2017b) Lifeskills Survey 2015 Report on Survey Findings 2017 [online] Available: <https://www.gov.ie/en/publication/07a9a3-lifeskills-survey/> [Accessed 03 Apr 2022].

Department of Education and Skills; (2018) Physical Education Framework: Senior Cycle. [online] Available: <https://ncca.ie/media/2687/seniorcyclephysicaleducation_framework.pdf#:~:text=Senior%20cycle%20physical%20education%20is%20designed%20to%20encourage,goals%20that%20are%20meaningful%20and%20relevant%20to%20them>. [Accessed 03 Apr 2022].

Department of Education (2021) Inspectorate [online] available: <https://www.gov.ie/en/organisation-information/818fa1-inspectorate/> [28 Mar 2022].

Department of Finance and Office of the Government Chief Information Officer, (n.d.) The Irish Government's Expenditure Data [online] available: <https://whereyourmoneygoes.gov.ie/en/2019/> [31 Mar 2022].

Department of Health (2013) ‘A framework for improved health and wellbeing 2013 – 2025’, Department of Health., Healthy Ir, 9–14.

Department of Health (2016) A Healthy Weight for Ireland: Obesity Policy and Action Plan 2016 – 2025, [online], available: <https://www.gov.ie/en/publication/c778a9-a-healthy-weight-for-ireland-obesity-policy-and-action-plan-2016-202/>. [accessed 10 Feb 2022].

Department of Health (2018) Healthy Ireland Summer Campaign 2018. [online], available: <https://www.gov.ie/en/press-release/cf3fba-healthy-ireland-summer-campaign-2018/> [accessed 09 Feb 2022].

Department of Health (2019a) ‘Healthy Ireland Fund 2019 - 2021. Expression of Interest Guidelines’, available: https://drive.google.com/file/d/1rqVbr_YILdi12GqeGNgPnILPaEbrCBKB/view.

Department of Health (2019b) Taoiseach and Ministers Launch Healthy Ireland Campaign [online], available: https://www.gov.ie/en/press-release/20cff4-taoiseach-and-health-ministers-to-launch-2019-healthy-ireland-campai/ [accessed 5 Aug 2021].

Department of Health (2019c) National Healthy Cities and Counties of Ireland Network [online], available: https://www.gov.ie/en/publication/d4fa22-healthy-counties-and-cities/#national-healthy-cities-and-counties-of-ireland-network [accessed 5 Aug 2021].

Department of Health (2020a) An Taoiseach, Minister for Health and Minister for Public Health, Wellbeing and the National Drugs Strategy Launch “Keep Well” Campaign. [online], available: <https://www.gov.ie/en/press-release/4db9d-an-taoiseach-minister-for-health-and-minister-for-public-health-well-being-and-the-national-drugs-strategy-launch-keep-well-campaign/> [accessed 09 Feb 2022].

Department of Health (2020b) Sláintecare Integration Projects 2020. Delivering Right Care, Right Place, Right Time [online] Available: <https://www.gov.ie/en/publication/0d2d60-slaintecare-publications/#slaintecare-integration-fund-projects> [Accessed 11 March 2022].

Department of Health and Children and Health Service Executive (2009) The National Guidelines on Physical Activity for Ireland [online], available: https://www.hse.ie/eng/about/who/healthwellbeing/our-priority-programmes/heal/heal-docs/the-national-guidelines-on-physical-activity-for-ireland.pdf [accessed 14 Feb 2022].

Department of Housing, Planning and Local Government (2018) Project Ireland 2040 National Planning Framework, [online] Available: <https://npf.ie/draft-of-ireland-2040/> [Accessed 20 Feb 2022].

Department of Housing, Planning and Local Government (2020). Local Authority Budgets 2020 [online] available: [<https://www.housing.old.gov.ie/sites/default/files/publications/files/final_2020_local_authority_budget_publication.pdf>](https://www.housing.old.gov.ie/sites/default/files/publications/files/final_2020_local_authority_budget_publication.pdf) [31 Mar 2022].

Department of Justice and Equality (2017) The Migrant Integration Strategy A Blueprint for the Future [online] available: <https://www.gov.ie/en/publication/983af-migrant-integration-strategy/> [Accessed 18 Feb 2022].

Department of Public Expenditure and Reform (2022) Budget 2022 Expenditure Report [online] available: <https://www.gov.ie/en/publication/cb7ac-expenditure-reports/> [Accessed 15 Feb 2022]

Department of Rural and Community Development (2017a) Realising our Rural Potential, the Government's Action Plan for Rural Development [online], available: <https://assets.gov.ie/2715/131118093828-a9da81a7a12b4f85aad3260c4f4bd80c.pdf> [accessed 10 Feb 2022].

Department of Rural and Community Development (2017b) Realising our Rural Potential, the Government's Action Plan for Rural Development First Progress Report August 2017[online], available: <https://data.oireachtas.ie/ie/oireachtas/committee/dail/32/joint_committee_on_rural_and_community_development/submissions/2017/2017-11-29_submission-realising-our-rural-potential-action-plan-for-rural-development-first-progress-report-summary-august-2017_en.pdf>

Department of Transport (2009a) Smartertravel – A Sustainable Transport Future. A New Transport Policy for Ireland 2009 - 2020. Dublin, Ireland. Available: <https://assets.gov.ie/19854/37d829c9748446349ff586045bfbcaba.pdf>

Department of Transport (2009b) Ireland’s First National Cycle Policy Framework. Dublin, Ireland. Available: <https://www.hse.ie/eng/about/who/healthwellbeing/our-priority-programmes/heal/healpublications/national-cycle-policy-framework.pdf>.

Department of Tourism, Transport and Sport (2018a) Strategy for the Future Development of National and Regional Greenways [online], available: <https://www.gov.ie/en/publication/ed5d17-strategy-for-the-future-development-of-national-and-regional-greenwa/> [accessed 10 Feb 2022].

Department of Transport Tourism and Sport (2018b) National Sports Policy 2018 – 2027, [online], available: https://www.gov.ie/en/press-release/20ca68-ministers-ross-and-griffin-launch-national-sports-policy-20182027/ [accessed 10 Feb 2022].

Department of Transport Tourism and Sport (2020) Review of actions of Smarter Travel Policy. Dublin, Ireland. Available: https://assets.gov.ie/40268/37bad63714d04a028d752d8e8df40743.pdf.

Department of Transport, Tourism and Sport and Department of Environment, Community and Local Government (2013), Design manual for Urban Roads and Streets, Dublin, Ireland. Available: <https://www.gov.ie/en/publication/3360b1-design-manual-for-urban-roads-and-streets/> [Accessed 04 Apr 2022].

Department of Tourism, Culture, Arts, Gaeltacht, Sport and Media (2019a) Sports Capital Programme [online], available: https://www.gov.ie/en/service/d13385-sports-capital-programme/ [accessed 10 Feb 2022].

Department of Tourism, Culture, Arts, Gaeltacht, Sport and Media (2019b) Large Scale Sport Infrastructure Fund (LSSIF) [online], available: <https://www.gov.ie/en/service/4113b3-large-scale-sport-infrastructure-fund-lssif/> [accessed 10 Feb 2022].

Department of Tourism, Culture, Arts, Gaeltacht, Sport and Media (2021) National Sports Policy 2018 – 2027 Sports Action Plan 2021 - 2023 [online] available: [<https://www.gov.ie/en/publication/93232-sports-action-plan-2021-2023/>](https://www.gov.ie/en/publication/93232-sports-action-plan-2021-2023/) [31 Mar 2022].

Desmond O’Mahony, Eoin McNamara, Rebecca McClintock, Aisling Murray, Emer Smyth and Dorothy Watson (2021) Growing Up in Ireland The Lives of 20-Year-Olds: Making the Transition to Adulthood Cohort ‘98 National Longitudinal Study of Children [online] available: <https://www.growingup.ie/pubs/The-Lives-of-20-Year-Olds.pdf> [31 Mar 2022].

Díaz del Castillo, A., Sarmiento, O.L., Reis, R.S., Brownson, R.C. (2011) ‘Translating evidence to policy: Urban interventions and physical activity promotion in Bogotá, Colombia and Curitiba, Brazil’, Translational Behavioral Medicine, 1(2), 350–360.

Director General of ANSES (2015) Opinion of the French Agency for Food, Environmental and Occupational Health & Safety on the ‘Updating of the PNNS Guidelines: Revision of the Guidelines Relating to Physical Activity and Sedentarity’ [online], available: https://www.anses.fr/en/system/files/NUT2012SA0155EN.pdf [accessed 1 Jan 2022].

Dubuy, V., De Cocker, K.A., De Bourdeaudhuij, I.M., Maes, L., Metsu, P., Van Acker, R., Cardon, G.M. (2013) ‘“10 000 Steps Flanders”: evaluation of the state-wide dissemination of a physical activity intervention in Flanders’, Health education research, 28(3), 546–551.

Dun Laoghaire-Rathdown Local Community Development Committee (2017) Department of Housing, Planning, Community and Local Government Communities Facilities Scheme Capital Scheme 2017 [online], available: <https://www.dlrcoco.ie/sites/default/files/atoms/files/dlr_lcdc_communitites_facilities_scheme_application_guidelines_2017.pdf> [accessed 10 Feb 2022].

Dunphy, L. (2021) Public Appetite Drives Cork City Street Pedestrianisation [online], The Irish Examiner, available: https://www.irishexaminer.com/news/munster/arid-40260710.html [accessed 11 Aug 2021].

Eighan, J., Walsh, B., Smith, S., Wren, M.A., Barron, S. and Morgenroth, E., 2019. A profile of physiotherapy supply in Ireland. *Irish Journal of Medical Science (1971-)*, *188*(1), pp.19-27.

Europa (2012) ‘Consolidated version of the treaty on the functioning of the European Union’, Official Journal of the European Union.

European Commission (2007) White Paper on Sport, European Commission;, Brussels.

European Commission (2013) Physical Education and Sport at School in Europe, Eurydice.  [online] available: <https://eacea.ec.europa.eu/national-policies/eurydice/content/physical-education-and-sport-school-europe_en>. [accessed 14 Feb 2022].

European Commission (2015) ‘Communication from the Commission to the European Parliament, the Council, the European Economic and Social Committee and the Committee of the Regions’.

European Commission (2016) Strategic Plan 2016-2020* DG Health & Food Safety, Brussels, Belgium, available: https://ec.europa.eu/info/sites/default/files/strategic-plan-2016-2020-dg-sante_may2016_en.pdf.

European Commission (2020) ‘Health Investments By European Structural and Investment Funds (Esif) 2014-2020’.

European Council (2000) ‘Conclusions of the Presidency (Annexes)’, Nice.

European Transport Safety Council (2021) Average Speed down 9% in Brussels since Launch of City-Wide 30 Km/h Limit [online], available: <https://etsc.eu/average-speed-down-9-in-brussels-since-launch-of-city-wide-30-km-h-limit/>.

Finance Act (2018) S. I. No. 30 of 2018, Dublin: Stationery Office, available: https://www.irishstatutebook.ie/eli/2018/act/30/enacted/en/html [Accessed 10 Feb 2022].

Fit Sport Austria GmbH (2016) Moving Children Healthily 2.0 [online], available: https://www.fitsportaustria.at/main.asp?VID=1&kat1=115&kat2=745&scsqs=1 [accessed 20 Aug 2021].

Fong, D.Y.T., Ho, J.W.C., Hui, B.P.H., Lee, A.M., Macfarlane, D.J., Leung, S.S.K., Cerin, E., Chan, W.Y.Y., Leung, I.P.F., Lam, S.H.S., Taylor, A.J., Cheng, K.K. (2012) ‘Physical activity for cancer survivors: Meta-analysis of randomised controlled trials’, BMJ (Online), 344(7844), 17.

Foster, C. (2000) Guidelines for Health-Enhancing Physical Activity Promotion Programmes, Citeseer.

Gaelic Athletic Association (2016a) Healthy Club [online], available: https://www.gaa.ie/my-gaa/community-and-health/healthy-club/ [accessed 12 Jan 2022].

Gaelic Athletic Association (2016b) About the GAA [online], available: <https://www.gaa.ie/the-gaa/about-the-gaa/> [accessed 12 Jan 2022].

Gavin, A., Költő, A., Kelly, C., Molcho, M., & Nic Gabhainn, S. (2021). Trends in Health Behaviours, Health Outcomes and Contextual Factors between 1998-2018: findings from the Irish Health Behaviour in School-aged Children Study. Dublin: Department of Health

Government of Australia (1997) Sport and Recreation: A Statistical Overview [online], available: https://www.abs.gov.au/ausstats/abs@.nsf/ProductsbyReleaseDate/987228A865C85060CA256DDD0073A368?OpenDocument [accessed 16 Aug 2021].

Government of the French Republic (2016) ‘Sommaire analytique’, Journal Officiel de la République Française, (304), 155, available: <https://www.legifrance.gouv.fr/jorf/jo/2016/12/31/0304>.

Government of Ireland (2019) Departments [online], available: https://www.gov.ie/en/help/departments/ [accessed 8 Oct 2021].

Government of Ireland (2020) Sláintecare Implementation Strategy & Action Plan 2021 — 2023 [online] available: <https://www.gov.ie/en/publication/6996b-slaintecare-implementation-strategy-and-action-plan-2021-2023/> [Accessed 23 Mar 2022].

Growing Up in Ireland (n.d.) About Growing Up in Ireland [online] available: <https://www.growingup.ie/about-growing-up-in-ireland/> [Accessed 15 Feb 2022]

Grunseit, A., Bellew, B., Goldbaum, E., Gale, J., Bauman, A. (2016) Mass Media Campaigns Addressing Physical Activity, Nutrition and Obesity in Australia 1996-2015, Sydney.

Guthold, R., Stevens, G.A., Riley, L.M., Bull, F.C. (2018) ‘Worldwide trends in insufficient physical activity from 2001 to 2016: a pooled analysis of 358 population-based surveys with 1·9 million participants’, The Lancet Global Health, 6(10), e1077–e1086, available: http://dx.doi.org/10.1016/S2214-109X(18)30357-7.

Hardinghaus, M., Wolf, C., Cyganski, R. (2021) ‘Case studies of new urban planning policy: effects of redesigning and redistributing public space in Europe’.

Hartley, J. (2010) ‘Political Leadership’, in Brookes, S. and Grint, K., eds., The New Public Leadership Challenge, Palgrave Macmillan: New York.

Harrington, D.M., Murphy, M., Carlin, A., Coppinger, T., Donnelly, A., Dowd, K.P., Keating, T., Murphy, N., Murtagh, E., O’Brien, W. and Woods, C., 2016. Results from Ireland north and South’s 2016 report card on physical activity for children and youth. Journal of physical activity and health, 13(s2), pp.S183-S188.

Harrington, J.M., Griffin, C., Vandevijvere, S. (2020) The Healthy Food Environment Policy Index (Food-EPI): Evidence Document for Ireland 2020, Cork, Ireland, available: https://www.jpi-pen.eu/images/reports/2 IE Evidence Document.pdf.

Hawkes C, Jewell J, Allen K. A food policy package for healthy diets and the prevention of obesity and diet-related non-communicable diseases: the NOURISHING framework. Obes Rev. 2013;14(S2):159–68.

Health and Social Care Professionals Council (2018) Annual Report 2018 [online], available: <https://www.coru.ie/files-publications/annual-reports/coru-annual-report-2018.pdf> [accessed 15 Feb 2022].

Health and Social Care Professionals Council (2019) Annual Report 2019 [online], available: <https://www.coru.ie/files-publications/annual-reports/coru-annual-report-2019.pdf> [accessed 15 Feb 2022].

Health and Social Care Professionals Council (2020) Annual Report 2020 [online], available: <https://www.coru.ie/files-publications/annual-reports/coru-annual-report-2020.pdf> [accessed 15 Feb 2022].

Health Service Executive (2012) The Health Promotion Strategic Framework Main Report [online], available: http://hdl.handle.net/10147/228940 [Accessed 10 Feb 2022]

Health Service Executive (2021) Making Every Contact Count [online], available: <https://www.hse.ie/eng/about/who/healthwellbeing/making-every-contact-count/about/about.html> [accessed 1 Jan 2022].

Healthy Ireland (2013) A Framework for Improving Health and Wellbeing 2013 – 2025 [online], available: <https://www.hse.ie/eng/services/publications/corporate/hieng.pdf> [accessed 09 Feb 2022].

Healthy Ireland (2016) Get Ireland Active! National Physical Activity Plan for Ireland, Dublin, Ireland, [online], available: <https://assets.gov.ie/7563/23f51643fd1d4ad7abf529e58c8d8041.pdf> [accessed 10 Feb 2022].

Healthy Ireland (2018) Healthy Ireland Survey 2018 [online], available: <https://assets.gov.ie/41139/80328fcb555d4ed0ac6c4b2ddcd7b677.pdf>. [accessed 10 Feb 2022].

Healthy Ireland, Department of Health (2019) Healthy Workplace Framework [online], available: https://www.gov.ie/en/publication/445a4a-healthy-workplace-framework/ [accessed 16 Aug 2021].

Healthy Ireland (2021) Healthy Ireland Strategic Action Plan 2021 – 2025 Building on the First Seven Years of Implementation. [online], available: https://www.gov.ie/en/press-release/ff2c6-government-launches-healthy-ireland-strategic-action-plan-2021-25/ [accessed 10 Feb 2022].

Healthy Ireland, Department of Health and Department of Tourism, Culture, Arts, Gaeltacht, Sport and Media (2021) [online] available: National Physical Activity Plan Implementation Summary 2019 [<https://www.gov.ie/en/policy-information/b60202-national-physical-activity/>](https://www.gov.ie/en/policy-information/b60202-national-physical-activity/) [Accessed 16 Feb 2022].

Healthy Spaces and Places (2009) ‘Design Principle-Mixed Land Use’, available: www.healthyplaces.org.au [accessed 22 Jul 2021].

Heath, G.W., Bilderback, J. (2019) ‘Grow healthy together: Effects of policy and environmental interventions on physical activity among urban children and youth’, Journal of Physical Activity and Health, 16(2), 172–176.

Hellemeier, C., Soltaniehha, M. (2010) Implementation and Results of the Traffic Circulation Plan in the City of Groningen.

Herman, W.H., Edelstein, S.L., Ratner, R.E., Montez, M.G., Ackermann, R.T., Orchard, T.J., Foulkes, M.A., Zhang, P., Saudek, C.D., Brown, M.B. (2013) ‘Effectiveness and cost-effectiveness of diabetes prevention among adherent participants’, American Journal of Managed Care, 19(3), 194–202.

Hernandez, J. (2008) Car-Free Streets, a Colombian Export, Inspire Debate [online], The New York Times, available: https://www.nytimes.com/2008/06/24/nyregion/24streets.html?_r=1&scp=1&sq=Ciclovía&st=cse [accessed 11 Aug 2021].

Houses of the Oireachtas (2017) Committee on the Future of Healthcare Sláintecare Report May 2017 [online] available: <https://www.oireachtas.ie/en/committees/32/future-of-healthcare/> [Accessed 23 Mar 2022].

Howlett, M., Giest, S., 2015. Policy Cycle, in: International Encyclopedia of the Social & Behavioral Sciences. Elsevier, pp. 288–292. https://doi.org/10.1016/B978-0-08-097086-8.75031-8

IBE-UNESCO (2021) Curriculum [online], available: <http://www.ibe.unesco.org/en/geqaf/core-resources/curriculum>. [Accessed 04 Apr 2022].

Institute of Public Health (2013) Proposed Sugar Sweetened Drinks Tax: Health Impact Assessment Full Report [online], available: <https://publichealth.ie/proposed-sugar-sweetened-drinks-tax-health-impact-assessment-full-report/> [accessed 15 Feb 2022].

Institute of Public Health (2019) Policy [online], available: <https://2019.iph.ie/index.php/policy> [accessed 5 Aug 2021].

Institute of Public Health (n.d.) Health Impact Assessment (HIA) [online], available: <https://publichealth.ie/hia-guidance/> [accessed 15 Feb 2022].

Ireland Active (n.d.) Governance [online] available: <https://irelandactive.ie/about-ireland-active/governance/> [Accessed 17 Feb 2022].

Irish Heart Foundation (n.d.) Y-PATH is hugely beneficial for students [online] available: <https://irishheart.ie/courses/y-path/schools/> [Accessed 20 Feb 2022].

Irish Physical Activity Research Collaboration (n.d.) I-PARC Evaluation Toolkit [online], available: <https://i-parc.ie/?page_id=955> [accessed 15 Feb 2022].

I-PARC team (n.d.) Irish Physical Activity Research Collaboration [online], available: I-PARC <https://i-parc.ie/?page_id=779> [accessed 15 Feb 2022].

Institute of Public Health (2019) Policy [online], available: https://2019.iph.ie/index.php/policy [accessed 5 Aug 2021].

International Society for Physical Activity and Health (2020) ISPAH’s Eight Investments That Work for Physical Activity [online], available: https://www.ispah.org/resources/key-resources/8-investments/ [accessed 14 Apr 2021].

Interreg Europe (2021) Good Practice: BICIPOLITANA Pesaro [online], Interreg Europe, available: https://www.interregeurope.eu/policylearning/good-practices/item/1239/bicipolitana-pesaro/ [accessed 11 Aug 2021].

Ipsos MRBI (2019) Irish Sport Monitor 2019 Core Questionnaire

<https://www.ucd.ie/issda/t4media/0050-05_irish_sports_monitor_2019_questionnaire.pdf> [31 Mar 2022].

Irish Medical Organisation (2012) IMO Position Paper on Health Inequalities [online], available: https://www.imo.ie/policy-international-affair/documents/imo-position-papers/imo-position-paper-on-hea/index.xml [accessed 16 Aug 2021].

Irish Wheelchair Association (n.d.) [online] available: <https://www.iwa.ie/sport/> [31 Mar 2022].

Irish Wheelchair Association, Sport Ireland and the Dormant Accounts Fund (2018) Great Outdoors. A guide for accessibility [online] available: <https://www.sportireland.ie/sites/default/files/2019-10/great-outdoors-a-guide-for-accessibility.pdf> [31 Mar 2022].

Jackson, N.W., Howes, F.S., Gupta, S., Doyle, J.L., Waters, E. (2005) ‘Interventions implemented through sporting organisations for increasing participation in sport’, Cochrane Database Syst Rev, (2), Cd004812.

Jerstad, S.J., Boutelle, K.N., Stice, E. (2011) ‘Depression in Adolescent Females’, 78(2), 268–272.

Kahlmeier, S., Wijnhoven, T.M.A., Alpiger, P., Schweizer, C., Breda, J., Martin, B.W. (2015) ‘National physical activity recommendations: Systematic overview and analysis of the situation in European countries’, BMC Public Health, 15(1)

KildareStreet (2019) *Written answers Tuesday, 16 April 2019* [online], available: <https://www.kildarestreet.com/wrans/?id=2019-04-16a.988> [accessed 09 Feb 2022].

Kilraine, J. (2021) ‘Plan for 30km/h speed limit in Dublin city set to be dropped.’, *Raidió Teilifís Éireann*, available <https://www.rte.ie/news/dublin/2021/0519/1222707-dublin-speed-limit/> [Accessed 08 Feb 2022].

KING Software (2021) The Work-Related Expenses Scheme (WKR): What Exactly Are the Rules? [online], Accountancy Van Morgen, available: https://www.accountancyvanmorgen.nl/2021/01/15/werkkostenregeling-wkr-precies-regels/ [accessed 16 Aug 2021].

Kokko, S., Kannas, L., Villberg, J. (2006) ‘The health promoting sports club in Finland - A challenge for the settings-based approach’, Health Promotion International, 21(3), 219–229.

Koski, P., Matarma, T., Pedisic, Z., Lane, A., Hartmann, H., Geidne, S., Hämäläinen, T., Nykänen, U., Rakovac, M., Livson, M., Savola, J. (2017) Sports Club for Health (SCforH) - Updated Guidelines for Health-Enhancing Sports Activities in a Club Setting, Helsinki, Finland.

Lakerveld, J., Woods, C., Hebestreit, A., Brenner, H., Flechtner-Mors, M., Harrington, J.M., Kamphuis, C.B.M., Laxy, M., Luszczynska, A., Mazzocchi, M., Murrin, C., Poelman, M., Steenhuis, I., Roos, G., Steinacker, J.M., Stock, C.C., van Lenthe, F., Zeeb, H., Zukowska, J., Ahrens, W. (2020) ‘Advancing the evidence base for public policies impacting on dietary behaviour, physical activity and sedentary behaviour in Europe: The Policy Evaluation Network promoting a multidisciplinary approach’, Food Policy.

Lassance, A. (2020) ‘What is a policy and what is a government program? A simple question with no clear answer, until now.’, SSRN.

Le Gouvernement du Grand-Duché de Luxembourg (2017) Gesond Iessen, Méi Bewegen Plan Cadre National 2018-2025, Luxembourg.

Lei Ravelo, J. (2018) Cities and NCDs: In Santo Domingo, Cars Cause More than Congestion [online], Devex, available: https://www.devex.com/news/cities-and-ncds-in-santo-domingo-cars-cause-more-than-congestion-92895 [accessed 11 Aug 2021].

Leeman, J., Sommers, J., Vu, M., Jernigan, J., Payne, G., Thompson, D., Heiser, C., Farris, R., Ammerman, A. (2012) ‘An evaluation framework for obesity prevention policy interventions’, Preventing Chronic Disease, 9(6), 1–9.

Leppo, K., Ollila, E., Pena, S., Wismar, M., Cook, S. (2013) Health in All Policies Seizing Opportunities, Implementing Policies, Finnish Ministry of Social Affairs and Health;, Helsinki, Finland.

Let’s Move! (2021) America’s Move to Raise A Healthier Generation of Kids [online], available: https://letsmove.obamawhitehouse.archives.gov/about [accessed 16 Aug 2021].

Liikkuva Aikuinen (2021) Porraspäivät [online], available: https://porraspaivat.fi/ [accessed 18 Aug 2021].

Lynn, T., Hiney, M. (2021) Outputs, Outcomes, and Emerging Impacts Results from HRB-Funded Awards That Completed in 2018–19 [online], Health Research Board: Dublin, Ireland, available: https://www.hrb.ie/fileadmin/2._Plugin_related_files/Publications/2019_Publication_files/Outputs_and_outcomes_2016_2017_report.pdf.

Management Advisory Committee (2010) ‘Connecting Government Whole of Government Responses to Australia’ s Priority Challenges’, available: http://www.directory.gov.au/directory?ea0_lf99_120.&organizationalUnit&b34767c6-3431-4d12-b56a-480e8660c912.

Massachusetts Department of Public Health (2021) Wellness Tax Credit Summary [online], available: https://www.mass.gov/service-details/wellness-tax-credit-summary [accessed 16 Aug 2021].

McDowell, C.P., Dishman, R.K., Gordon, B.R., Herring, M.P. (2019) ‘Physical activity and anxiety: a systematic review and meta-analysis of prospective cohort studies’, American Journal Of Preventive Medicine, 57(4), 545–556.

McDowell, C.P., Dishman, R.K., Vancampfort, D., Hallgren, M., Stubbs, B., MacDonncha, C., Herring, M.P. (2018) ‘Physical activity and generalized anxiety disorder: results from The Irish Longitudinal Study on Ageing (TILDA)’, International Journal of Epidemiology, 47(5), 1443–1453.

Meadows, D. (1999) ‘Leverage Points: Places to Intervene in a Systems’, The Sustainability Institute, 1–19.

Mitchell L, Bel-Serrat S, Stanley I, Hegarty T, McCann L, Mehegan J, Murrin C, Heinen M, Kelleher C (2020). The Childhood Obesity Surveillance Initiative (COSI) in the Republic of Ireland – Findings from 2018 and 2019.

Mori, N., Armada, F. and Willcox, D.C., 2012. Walking to school in Japan and childhood obesity prevention: new lessons from an old policy. *American journal of public health*, *102*(11), pp.2068-2073.

Morris, J.N., Crawford, M.D. (1958) ‘Coronary Heart Disease and Physical Activity of Work Evidence of a National Necropsy Survey’, British Medical Journal, 262(5111), 1486–1496.

Morris, J.N., Heady, J.A., Raffle, P.A.B., Roberts, C.G., Parks, J.W. (1953a) ‘Coronary Heart-Disease and Physical Activity of Work’, The Lancet, 262(6796), 1053–1057.

Morris, J.N., Heady, J.A., Raffle, P.A.B., Roberts, C.G., Parks, J.W. (1953b) ‘Coronary Heart-Disease and Physical Activity of Work’, The Lancet, 262, 1111–1120.

Mullaney, C., Gleeson., M., Quinn, G., Leane, G., Humphreys, M., McKeon, M., Kennedy, B., Doherty, L., Jordan, K., (2017) Living Well with a Chronic Condition: Framework for Self-management Support [online] Available: <https://www.hse.ie/eng/health/hl/selfmanagement/hse-self-management-support-final-document1.pdf#:~:text=The%20National%20Self-management%20Support%20Framework%20for%20Chronic%20Conditions%3A,their%20conditions%2C%20through%20collaborative%20relationships%20and%20supportive%20interventions>. [Accessed 11 March 2022].

Murphy, J.J., Mansergh, F., Murphy, M.H., Murphy, N., Cullen, B., O’Brien, S., Finn, S., O’Donoghue, G., Barry, N., O’Shea, S. and Leyden, K.M., 2021. “Getting Ireland Active”—Application of a Systems Approach to Increase Physical Activity in Ireland Using the GAPPA Framework. *Journal of Physical Activity and Health*, *18*(11), pp.1427-1436.

National Children’s Office (2004) Ready, Steady, Play! A National Play Policy [online] Available: <https://assets.gov.ie/24440/03bb09b94dec4bf4b6b43d617ff8cb58.pdf#:~:text=The%20NCS%20is%20a%20ten-year%20strategy%20to%20guide,cultural%20activities%20to%20enrich%20their%20experience%20of%20childhood%E2%80%99>. [Accessed 17 Feb 2022].

National Council for Curriculum and Assessment (2017a) Guidelines for Wellbeing in Junior Cycle 2017, available: <https://www.ncca.ie/media/2487/wellbeingguidelines_forjunior_cycle.pdf>.

National Council for Curriculum and Assessment (2017b) Physical Education Curriculum Specification [online] available: <https://www.peai.org/wp-content/uploads/2017/12/SCPE_Framework-en.pdf>

National Institute for Health and Care Excellence (2020) Behaviour Change: Digital and Mobile Health Interventions, available: <https://www.nice.org.uk/guidance/gid-ng10101/documents/final-scope>.

National Transport Authority (2011) National Cycle Manual. [online] Available: <https://www.cyclemanual.ie/> [Accessed 08 Feb 2022].

National Transport Authority (2015) Permeability Best Practice Guide. Dublin, Ireland. Available: [[https://www.nationaltransport.ie/permeability-in-existing-urban-areas-best-practice-guide/https://www.nationaltransport.ie/permeability-in-existing-urban-areas-best-practice-guide/](https://www.nationaltransport.ie/permeability-in-existing-urban-areas-best-practice-guide/)](https://www.nationaltransport.ie/permeability-in-existing-urban-areas-best-practice-guide/) [Accessed 09 Feb 2022]

National Transport Authority (2022) National Bike Week 14th - 22nd May 2022 [online], available: https://www.nationaltransport.ie/tfi-smarter-travel/cycle-challenge-ie/bikeweek/ [accessed 20 Jan 2022].

New South Wales Government (2020) Policy in Practice [online], available: https://education.nsw.gov.au/teaching-and-learning/curriculum/key-learning-areas/pdhpe/physical-education/policy-in-practice#Curriculum0 [accessed 27 Aug 2021].

North Carolina Division of Public Health (2014) ‘Shared Use of School Property in North Carolina: The Role of Unorganized Recreation’.

Norwegian Ministry of Local Government and Modernization (2019) ‘Fourth Norwegian action plan open government partnership (OGP) 2019-2021’, 2019–2021.

Norwood, P., Eberth, B., Farrar, S., Anable, J., Ludbrook, A. (2014) ‘Active travel intervention and physical activity behaviour: An evaluation’, Social Science and Medicine, 113, 50–58, available: http://dx.doi.org/10.1016/j.socscimed.2014.05.003.

Nutbeam, D., Kickbusch, I. (1998) ‘Health promotion glossary’, Health Promotion International, 13(4), 349–364.

Office for the Promotion of Migrant Integration, (2019) The Migrant Integration Strategy 2017-2020 [online] available: <https://www.gov.ie/en/publication/983af-migrant-integration-strategy/> [Accessed 18 Feb 2022]

Oireachtas.ie (2020) Health Promotion Dáil Éireann Debate, Tuesday - 28 July 2020 [online], available: <https://www.oireachtas.ie/en/debates/question/2020-07-28/852/> [accessed 09 Feb 2022].

O’Mahoney, C. (2021) Official Launch of European Week of Sport 2021 [online] Available: <https://www.sportireland.ie/participation/news/official-launch-of-european-week-of-sport-2021#:~:text=A%20European%20Commission%20led%20initiative,healthier%20and%20more%20active%20lifestyle>. [Accessed 15 Feb 2022]

Organisation for Economic Co-operation and Development, European Union (2020) Health at a Glance: Europe 2020: State of Health in the EU Cycle, Paris, France.

Paffenbarger, R.S., Hyde, R.T., Wing, A.L., Hsieh, C.-C. (1986) ‘Physical Activity, All-cause Mortality, and Longevity of College Alumni’, The New England Journal of Medicine, 314(10), 605–613.

Paffenbarger, R.S., Laughlin, M.E., Gima, A.S., Black, R.A. (1970) ‘Work Activity of Longshoremen as Related to Death from Coronary Heart Disease and Stroke’, The New English Journal of medicine, 282(20), 1109–1113.

Panter, J.R., Jones, A.P., van Sluijs, E.M.F. (2008) ‘Environmental determinants of active travel in youth: A review and framework for future research’, International Journal of Behavioral Nutrition and Physical Activity, 5, 1–14.

ParticipACTION (2022) The ParticipACTION Archive Project [online], available: http://digital.scaa.sk.ca/gallery/participaction/english/motivate/index.html [accessed 8 Jan 2022].

Peters, D., Harting, J., Van Oers, H., Schuit, J., De Vries, N., Stronks, K. (2016) ‘Manifestations of integrated public health policy in Dutch municipalities’, Health Promotion International, 31(2), 290–302.

Physiotherapists Registration Board (2019) ‘Physiotherapists Registration Board Code of Professional Conduct and Ethics’, available: https://coru.ie/files-codes-of-conduct/prb-code-of-professional-conduct-and-ethics-for-physiotherapists.pdf [accessed 20 Jan 2022].

Physiotherapists Registration Board Approved Qualifications Bye-Law (2020) S. I. 187 of 2020 Dublin: Stationery Office, available: <https://www.irishstatutebook.ie/eli/2020/si/187/made/en/print> [accessed 15 Feb 2022].

Pobal (2016) ‘The 2016 Pobal HP Deprivation Index for Small Areas (SA) ’, available: <https://maps.pobal.ie/WebApps/DeprivationIndices/index.html>.

Public Health (Alcohol) Act (2018) S. I. No. 24 of 2018, Dublin: Stationery Office, available: <https://www.irishstatutebook.ie/eli/isbc/2018_24.html> [Access 24 Mar 2022].

Public Heath England (2014) ‘Everybody Active, Every Day’, Public Health England, (October), 1–23, available: www.gov.uk/phe%0Awww.gov.uk/phe%5Cnwww.facebook.com/PublicHealthEngland%0Ahttps://assets.publishing.service.gov.uk/government/uploads/system/uploads/attachment_data/file/601298/Everybody_active_every_day_2_years_on.pdf.

Public Health Interventions Advisory Committee, NICE project team (2013) ‘Physical activity : brief advice for adults in primary care’, NICE Guidelines, (May), 63.

Pucher, J., Buelher, R. (2007) ‘At the Frontiers of Cycling: Policy innovations in the Netherlands, Denmark and Germany’, World Transport Policy and Practice, 13(3), 8–57.

Register of Exercise Professionals Ireland (2020) About CPD [online], available: https://repsireland.ie/about-cpd/ [accessed 20 Jan 2022].

Regulation of Lobbying Act (2015) S. I. No. 5 of 2015, Dublin: Stationery Office, available: <https://www.irishstatutebook.ie/eli/2015/act/5/enacted/en/html> [Access 10 Feb 2022]

Reid, C. (2020) How a Belgian Port City Inspired Birmingham’s Car-Free Ambitions [online], The Guardian, available: https://www.theguardian.com/environment/2020/jan/20/how-a-belgian-port-city-inspired-birminghams-car-free-ambitions [accessed 11 Aug 2021].

Revenue Commissioners (2021) Cycle to Work Scheme [online], available: https://www.revenue.ie/en/jobs-and-pensions/taxation-of-employer-benefits/cycle-to-work-scheme.aspx [accessed 16 Aug 2021].

Rhode Island Office of Library and Information Services (2021) EDI Basics - Equity, Diversity and Inclusion - LibGuides at Rhode Island Office of Library and Information Services [online], available: https://olis-ri.libguides.com/edi [accessed 22 Jul 2021].

Road Traffic Act (2004) S. I. No. 44 of 2004, Dublin: Stationery Office, available: <https://www.irishstatutebook.ie/eli/2004/act/44/enacted/en/html> [Accessed 08 Feb 2022].

Robert Woods Johnson Foundation (2021) What Is a Culture of Health? | Evidence for Action [online], available: https://www.evidenceforaction.org/about-us/what-culture-health [accessed 21 Jul 2021].

Ruetten, A., Schow, D., Breda, J., Galea, G., Kahlmeier, S., Oppert, J.M., van der Ploeg, H., van Mechelen, W. (2016) ‘Three types of scientific evidence to inform physical activity policy: results from a comparative scoping review’, Int J Public Health, 61(5), 553–563, available: <https://www.ncbi.nlm.nih.gov/pubmed/27113707>.

Safefood (2022) Being More Active [online], available: https://www.safefood.net/start/being-more-active [accessed 31 Jan 2022].

Sallis, J.F., Cervero, R.B., Ascher, W., Henderson, K.A., Kraft, M.K., Kerr, J. (2006) ‘An ecological approach to creating active living communities’, Annual Review of Public Health, 27, 297–322.Schöpfel, J. (2010) ‘Towards a Prague definition of Grey Literature’, GL-Conference Series: Conference Proceedings, 11–24.

Sallis, J.F., 2009. Measuring physical activity environments: a brief history. American journal of preventive medicine, 36(4), pp.S86-S92.

Sallis, RE, Matuszak, JM, Baggish, AL, et al. Call to action on making physical activity assessment and prescription a medical standard of care. Curr Sports Med Rep. 2016;15:207-214.

Schöpfel, J. (2011) ‘Towards a Prague definition of Grey Literature’, GL-Conference Series: Conference Proceedings, 11–24.

Shi, L., Halik, Ü., Abliz, A., Mamat, Z., Welp, M. (2020) ‘Urban green space accessibility and distribution equity in an arid oasis city: Urumqi, China’, Forests, 11(6).

Spacey, J. (2015) 2021 Health And Sports Day [online], Japan Talk, available: https://www.japan-talk.com/jt/new/health-and-sports-day [accessed 16 Aug 2021].

Sport-ordonnance.fr (2017) Le Sport Sur Ordonnance Pour Se Soigner [online], sport-ordonnance.fr, available: https://sport-ordonnance.fr/ [accessed 11 Aug 2021].

Sport England (2021) Active Lives [online], available: https://www.sportengland.org/know-your-audience/data/active-lives?section=methodology [accessed 16 Aug 2021].

Sport Ireland (2018a) Sport Ireland Policy on Participation in Sport by People with Disabilities [online], available: <https://www.sportireland.ie/sites/default/files/2019-12/sport-ireland-policy-on-participation-in-sport-by-people-with-disabilities.pdf> [accessed 10 Feb 2022].

Sport Ireland (2018b) Women in Sport Policy [online], available: <https://www.sportireland.ie/sites/default/files/2019-11/wis_policy.pdf> [accessed 10 Feb 2022].

Sport Ireland (2019) ‘Community Sports and Physical Activity Hubs Evaluation’, 72, available: <https://www.sportireland.ie/sites/default/files/2019-10/csh_evaluation.pdf>.

Sport Ireland and Ipsos MRBI (2019) Irish Sport Monitor Annual Report 2019 [online], available: https://www.sportireland.ie/sites/default/files/media/document/2020-09/irish-sports-monitor-2019-report-lower-res.pdf [Accessed 10 Feb 2022].

Sport Ireland (2020) Dormant Accounts Funding September 2020. [online] available: <https://www.sportireland.ie/sites/default/files/media/document/2020-09/dormant-accounts-funding-allocations-2020.pdf#:~:text=Sport%20Ireland%20Active%20Cities%20The%20Active%20Cities%20fund,to%20create%20social%20norms%20about%20the%20benefits%20of> [Accessed 18 Feb 2022].

Sport Ireland (2021a) Participation Plan 2021-2024 Increasing Participation in a Changing Ireland [online] available: [<https://www.sportireland.ie/sites/default/files/media/document/2021-05/plan_final_eng-000001.pdf>](https://www.sportireland.ie/sites/default/files/media/document/2021-05/plan_final_eng-000001.pdf) [Accessed 03 Apr 2022].

Sport Ireland (2021b) Let's Get Back [online], available: <https://www.sportireland.ie/LetsGetBack> [accessed 09 Feb 2022].

Sport Ireland (2021c) Sport Ireland seeking input from the public to inform the new Diversity & Inclusion Policy [online] available: <https://www.sportireland.ie/ethics/news/sport-ireland-seeking-input-from-the-public-to-inform-the-new-diversity-inclusion#:~:text=The%20Sport%20Ireland%20Diversity%20%26%20Inclusion%20policy%20will,relation%20to%20participation%20in%20sport%20and%20physical%20activity%E2%80%9D> [31 Mar 2022].

Sport Ireland (2021d) Sport Ireland’s Research Strategy 2021 – 2027 [online] available: <https://www.sportireland.ie/sites/default/files/media/document/2021-12/strategy_0.pdf> [Access 24 Mar 2022].

Sport Ireland (n.d.) Find Your Trails [online] available: <https://www.sportireland.ie/outdoors/find-your-trails> [29 Mar 2022].

Sport Ireland (n.d.) Research Funding Scheme [online], available: <https://www.sportireland.ie/research/research-funding-scheme> [accessed 15 Feb 2022].

Sport Ireland (n.d.) Coaching Development Programme for Ireland [online] available: <https://www.sportireland.ie/coaching/coaching-development-programme-for-ireland/coaching-development-programme-for-ireland> [Accessed 17 Feb 2022].

Swinburn, B., Sacks, G., Vandevijvere, S., Kumanyika, S., Lobstein, T., Neal, B., Barquera, S., Friel, S., Hawkes, C., Kelly, B., L’Abbé, M., Lee, A., Ma, J., Macmullan, J., Mohan, S., Monteiro, C., Rayner, M., Sanders, D., Snowdon, W., Walker, C. (2013) ‘INFORMAS (International Network for Food and Obesity/non-communicable diseases Research, Monitoring and Action Support): Overview and key principles’, Obesity Reviews, 14(S1), 1–12.

Tanaka, C., Tanaka, S., Inoue, S., Miyachi, M., Suzuki, K. and Reilly, J.J., 2016. Results from Japan’s 2016 report card on physical activity for children and youth. *Journal of physical activity and health*, *13*(s2), pp.S189-S194.

The Joint Programming Initiative ‘A Healthy Diet for a Healthy Life, 2017. Joint Funding Action “Effectiveness of existing policies for lifestyle interventions – Policy Evaluation Network (PEN).”Tremblay, M.S., Aubert, S., Barnes, J.D., Saunders, T.J., Carson, V., Latimer-Cheung, A.E., Chastin, S.F.M., Altenburg, T.M., Chinapaw, M.J.M., Altenburg, T.M., Aminian, S., Arundell, L., Atkin, A.J., Aubert, S., Barnes, J., Barone Gibbs, B., Bassett-Gunter, R., Belanger, K., Biddle, S., Biswas, A., Carson, V., Chaput, J.-P., Chastin, S., Chau, J., ChinAPaw, M., Colley, R., Coppinger, T., Craven, C., Cristi-Montero, C., de Assis Teles Santos, D., del Pozo Cruz, B., del Pozo-Cruz, J., Dempsey, P., do Carmo Santos Gonçalves, R.F., Ekelund, U., Ellingson, L., Ezeugwu, V., Fitzsimons, C., Florez-Pregonero, A., Friel, C.P., Fröberg, A., Giangregorio, L., Godin, L., Gunnell, K., Halloway, S., Hinkley, T., Hnatiuk, J., Husu, P., Kadir, M., Karagounis, L.G., Koster, A., Lakerveld, J., Lamb, M., Larouche, R., Latimer-Cheung, A., LeBlanc, A.G., Lee, E.-Y., Lee, P., Lopes, L., Manns, T., Manyanga, T., Martin Ginis, K., McVeigh, J., Meneguci, J., Moreira, C., Murtagh, E., Patterson, F., Rodrigues Pereira da Silva, D., Pesola, A.J., Peterson, N., Pettitt, C., Pilutti, L., Pinto Pereira, S., Poitras, V., Prince, S., Rathod, A., Rivière, F., Rosenkranz, S., Routhier, F., Santos, R., Saunders, T., Smith, B., Theou, O., Tomasone, J., Tremblay, M., Tucker, P., Umstattd Meyer, R., van der Ploeg, H., Villalobos, T., Viren, T., Wallmann-Sperlich, B., Wijndaele, K., Wondergem, R., on behalf of SBRN Terminology Consensus Project Participants, 2017. Sedentary Behavior Research Network (SBRN) – Terminology Consensus Project process and outcome. International Journal of Behavioral Nutrition and Physical Activity 14, 75. https://doi.org/10.1186/s12966-017-0525-8UNESCO (2015) Quality Physical Education. Guidelines For Policymakers, Paris, France.

The Teaching Council (2020a) Annual Report 2019/2020 [online], available: <https://www.teachingcouncil.ie/en/publications/annual-reports/annual-report-2019-2020.pdf> [accessed 15 Feb 2022].

The Teaching Council of Ireland (2020b) Teaching Council Registration Curricular Subject Requirements (Post-Primary) For persons applying for registration on or after 1 January 2023 [online], available: <https://www.teachingcouncil.ie/en/publications/registration/curricular-subject-requirements-after-january-2023.pdf> [accessed 15 Feb 2022].

Thiry, C. (2020) Summary of Good Move Regional Mobility Plan 2020-2030 of the Brussels-Capital Region, Brussels, Belgium.

Tsubohara, S. (2007) The Effect and Modification of the Traffic Circulation Plan (VCP) - Traffic Plannign in Groningen in the 1980s, Groningen.

UNESCO (2015) Quality Physical Education. Guidelines For Policy-Makers, Paris, France.

Valdivia, A. (2018) Mayor David Collado Travels Santo Domingo by Bicycle [online], Municipiosaldia.com, available: https://do.municipiosaldia.com/sureste/ozama/distrito-nacional/item/29693-alcalde-david-collado-recorre-santo-domingo-en-bicicleta [accessed 11 Aug 2021].

Van Der Zee, R. (2015) How Groningen Invented a Cycling Template for Cities All over the World | Cities | The Guardian [online], available: https://www.theguardian.com/cities/2015/jul/29/how-groningen-invented-a-cycling-template-for-cities-all-over-the-world [accessed 11 Aug 2021].

Vandevijvere, S., Swinburn, B. (2015) ‘Getting serious about protecting New Zealand children against unhealthy food marketing’, New Zealand Medical Journal, 128(1422), 85–87.

Vandevijvere, S., Swinburn, B. (2017) ‘INFORMAS Protocol Public Sector Module: Healthy Food Environment Policy Index Extent of implementation of food environment policies by governments compared to international best practice & priority recommendations’, Informas, (June), 1–35.

Varney, J., Fenton, K. (2015) Moving Healthcare Professionals at Every Level [online], Uk Health Security Agency, available: https://ukhsa.blog.gov.uk/2015/10/19/moving-healthcare-professionals-at-every-level-2/ [accessed 20 Jan 2022].

Vedung, E., Rist, R.C. (2007) ‘Policy Instruments: Typologies and Theories’, in Bemelmans-Videc, M.-L., Rist, R.C. and Vedung, E., eds., Carrots, Sticks & Sermons: Policy Instruments & Their Evaluation, Transaction Publishers: New Brunswick, NJ, 21–58.

VicHealth (2005) The Story of VicHealth: A World First in Health Promotion [online], available: https://www.vichealth.vic.gov.au/media-and-resources/publications/the-story-of-vichealth [accessed 16 Aug 2021].

VicHealth (2012) 521 Sports Clubs in Victoria Receive Active Clubs Grants [online], available: https://www.vichealth.vic.gov.au/search/521-sports-clubs-in-victoria-receive-active-clubs-grants [accessed 26 Mar 2021].

VicHealth (2019) Active Club Grants – Sport Grants and Funding [online], available: https://www.vichealth.vic.gov.au/funding/active-club-grants [accessed 26 Mar 2021].

Weichselbaum, E., Hooper, B., Ballam, R., Buttriss, J., Strigler, F., Oberritter, H., Bagus, T., Cullen, M., Palacios, N., Valero, T., Ruiz, E., del Pozo, S., Ávila, J.M., Varela-Moreiras, G. (2012) ‘Physical activity in schools across europe’, Nutrition Bulletin, 37(3), 262–269.

White, L., Luciani, A., Berry, T.R., Deshpande, S., Latimer-Cheung, A., O’Reilly, N., Rhodes, R.E., Spence, J.C., Tremblay, M.S., Faulkner, G. (2016) ‘Sports day in Canada: a longitudinal evaluation’, International Journal of Health Promotion and Education, 54(1), 12–23.

World Cancer Research Fund (2021) Moving Database [online], available: https://policydatabase.wcrf.org/level_one?page=moving-level-one#step2=0#step3=398 [accessed 20 Aug 2021].

World Health Organisation Regional Office for Europe (2018) ‘Physical Activity Factsheets For The 28 European Union Member States’, 148, available: http://www.euro.who.int/en/health-topics/disease-prevention/physical-activity/data-and-statistics/physical-activity-fact-sheets.

Woods C., McCaffrey N., Furlong B., Fitzsimons-D’Arcy L., Murphy M., Harrison M., Glynn L., O’Riordan J., O’Neill B., Jennings S. and Peppard C. (2016) The National Exercise Referral Framework. Health and Wellbeing Division, Health Service Executive. Dublin. Ireland.

Woods CB, Powell C, Saunders JA, O’Brien W, Murphy MH, Duff C, Farmer O, Johnston A., Connolly S. and Belton S. (2018). The Children’s Sport Participation and Physical Activity Study 2018 (CSPPA 2018). Department of Physical Education and Sport Sciences, University of Limerick, Limerick, Ireland, Sport Ireland, and Healthy Ireland, Dublin, Ireland and Sport Northern Ireland, Belfast, Northern Ireland.

Woods, C.B., Volf, K., Kellly, L., Casey, B., Gelius, P., Messing, S., Forberger, S., Lakerveld, J., Zukowska, J., Bengoechea, E.G. (2020) ‘The evidence for the impact of policy on physical activity outcomes within the school-setting: A Systematic Review.’, Journal of Sport and Health Sciences.

World Cancer Research Fund (2021) Moving Database [online], available: https://policydatabase.wcrf.org/level_one?page=moving-level-one#step2=0#step3=398 [accessed 20 Aug 2021].

World Health Organization (2007) Promoting Physical Activity in Schools: An Important Element of a Health-Promoting School, WHO information series on school health, Geneva, Switzerland, available: http://whqlibdoc.who.int/publications/2007/9789241595995_eng.pdf.

World Health Organization (2008) ‘MPower A Policy Package to Reverse the Tobacco Epidemic’, WHO report on the global tobacco epidemic, (MPOWER package), available: <http://www.who.int/blindness/AP2014_19_Russian.pdf?ua=1>.

World Health Organisation (1978) Alma Ata Declaration, Alma Ata, Kazakhstan.

World Health Organization (2021) Noncommunicable Diseases [online], available: https://www.who.int/news-room/fact-sheets/detail/noncommunicable-diseases [accessed 16 Jul 2021].

World Health Organisation (2018) Global Action Plan on Physical Activity 2018-2030: More Active People for a Healthier World, World Health Organization: Geneva.

World Health Organisation Regional Office for Europe (2016) ‘Physical Activity Strategy for the WHO European Region 2016 - 2025’, available: http://www.euro.who.int/__data/assets/pdf_file/0010/282961/65wd09e_PhysicalActivityStrategy_150474.pdf.

World Health Organisation Regional Office for Europe (2018) Promoting physical activity in the health sector, available: <https://www.euro.who.int/en/health-topics/disease-prevention/physical-activity/data-and-statistics/physical-activity-fact-sheets/physical-activity-factsheets-2018/promoting-physical-activity-in-the-health-sector-2018>https://www.euro.who.int/en/health-topics/disease-prevention/physical-activity/data-and-statistics/physical-activity-fact-sheets/physical-activity-factsheets-2018/promoting-physical-activity-in-the-health-sector-2018

World Health Organisation Regional Office for Europe, European Commission (2021) 2021 Physical Activity Factsheets for the European Union Member States in the WHO European Region, Copenhagen, Denmark.

World Health Organisation Regional Office for Europe (2021) Evidence-Informed Policy-Making [online], available: https://www.euro.who.int/en/data-and-evidence/evidence-informed-policy-making/evidence-informed-policy-making [accessed 29 Jun 2021].

Zając, A. (2019) ‘Ghent – Innovative Strategic Urban Development: New Transitions in the City and along the Old Dockyards’, Real Corp 2019, 4(April), 895–900, available: <https://conference.corp.at/archive/CORP2019_32.pdf>.

Zarpalas, D. (2016) ‘PATHway dissemination plan’, (March).

Zähringer, J., Schwingshackl, L., Movsisyan, A., Stratil, J.M., Capacci, S., Steinacker, J.M., Forberger, S., Ahrens, W., Küllenberg De Gaudry, D., Schünemann, H.J., Meerpohl, J.J. (2020) ‘Use of the GRADE approach in health policymaking and evaluation: A scoping review of nutrition and physical activity policies’, Implementation Science, 15(1), 1–18.
