# Supplementary material for: Assessing the implementation of physical activity-promoting public policies in the Republic of Ireland: a study using the Physical Activity Environment Policy Index (PA-EPI)
Source: Health Res Policy Syst. 2023 Jun 26;21:63. doi: 10.1186/s12961-023-01013-6 (PMC10291784; doi:10.1186/s12961-023-01013-6)
Supplement: Supplementary file 3 — Additional file 3. Criteria for Prioritisation. [file 12961_2023_1013_MOESM3_ESM.docx]

**Additional file 3 Criteria for prioritisation**

**Importance criteria**

| **Need** | **Impact** | **Other positive effects** | **Other negative effects** |
| --- | --- | --- | --- |
| The size of the implementation gap. | The effectiveness of the action on improving physical activity environments (including reach and effect size). | For example, on protecting rights of children | For example: regressive effects on household income, infringement of personal liberties |

**Achievability criteria**

| **Feasibility** | **Acceptability** | **Affordability** | **Efficiency** |
| --- | --- | --- | --- |
| How easy or hard the action is to implement | The level of support from key stakeholders including government, the public, public health and industry | The cost of implementing the action | The cost-effectiveness of the action |

**Equity criteria**

| **Socio-economic effect** | **Structures vs. Individuals** |
| --- | --- |
| Progressive/regressive effects on reducing physical activity-related inequalities | The extent to which a given policy requires environmental change rather than individual choices |
